# Conjugates of 3,5-Bis(arylidene)-4-piperidone and Sesquiterpene Lactones Have an Antitumor Effect via Resetting the Metabolic Phenotype of Cancer Cells

**DOI:** 10.3390/molecules29122765

**Published:** 2024-06-11

**Authors:** M. E. Neganova, Yu. R. Aleksandrova, E. V. Sharova, E. V. Smirnova, O. I. Artyushin, N. S. Nikolaeva, A. V. Semakov, I. A. Schagina, N. Akylbekov, R. Kurmanbayev, D. Orynbekov, V. K. Brel

**Affiliations:** 1 Institute of Physiologically Active Compounds at Federal Research Center of Problems of Chemical Physics and Medicinal Chemistry, Russian Academy of Sciences, 142432 Chernogolovka, Russia; neganovam@ipac.ac.ru (M.E.N.); aleksandrova@ipac.ac.ru (Y.R.A.); nikolaevans@ipac.ac.ru (N.S.N.); l_vok@list.ru (A.V.S.); schagina.in@yandex.ru (I.A.S.); 2 Nesmeyanov Institute of Organoelement Compounds, Russian Academy of Sciences, 119991 Moscow, Russia; sharova@mail.ru (E.V.S.); matveeva@gmail.com (E.V.S.); oleg.artyushin@gmail.com (O.I.A.); 3 Laboratory of Engineering Profile “Physical and Chemical Methods of Analysis”, Korkyt Ata Kyzylorda University, Aiteke bi Str. 29A, 120014 Kyzylorda, Kazakhstan; nurgali_089@mail.ru (N.A.); rakhat@mail.ru (R.K.)

**Keywords:** 3,5-bis(arylidene)-4-piperidone, sesquiterpene lactones, dehydrocostuslactone, alantolactone, azides, acetylenes, click chemistry, cytotoxicity, glycolysis

## Abstract

In recent years, researchers have often encountered the significance of the aberrant metabolism of tumor cells in the pathogenesis of malignant neoplasms. This phenomenon, known as the Warburg effect, provides a number of advantages in the survival of neoplastic cells, and its application is considered a potential strategy in the search for antitumor agents. With the aim of developing a promising platform for designing antitumor therapeutics, we synthesized a library of conjugates of 3,5-bis(arylidene)-4-piperidone and sesquiterpene lactones. To gain insight into the determinants of the biological activity of the prepared compounds, we showed that the conjugates of 3,5-bis(arylidene)-4-piperidone and sesquiterpene lactones, which are cytotoxic agents, demonstrate selective activity toward a number of tumor cell lines with glycolysis-inhibiting ability. Moreover, the results of molecular and in silico screening allowed us to identify these compounds as potential inhibitors of the pyruvate kinase M2 oncoprotein, which is the rate-determining enzyme of glycolysis. Thus, the results of our work indicate that the synthesized conjugates of 3,5-bis(arylidene)-4-piperidone and sesquiterpene lactones can be considered a promising platform for designing selective cytotoxic agents against the glycolysis process, which opens new possibilities for researchers involved in the search for antitumor therapeutics among compounds containing piperidone platforms.

## 1. Introduction

Cancer is the second leading cause of death worldwide and the leading cause of death in humans before the age of 85 years [1,2,3]. In spite of continuous efforts in biomedical chemistry and tangible accomplishments in cancer treatment, morbidity and mortality rates are still very high, mainly due to numerous side effects resulting from chemotherapeutic medication [4,5,6]. Therefore, the development of less toxic for healthy microenvironments and more efficient strategies for malignant neoplasm treatment is still a frontier of contemporary research programs in the search for promising antitumor agents.

One of the promising medicinal agents is curcumin, a highly active component of turmeric extract with an endless range of therapeutic properties, such as antioxidant, antitumor, antimicrobial, neuroprotective, and other types of activity [7,8,9]. However, the achievement of the therapeutic effect of curcumin is confined by a number of considerable drawbacks due to its low solubility in water, fast metabolic degradation, instability, and low bioavailability [10,11,12,13], which considerably limits its further pharmaceutical application and provides no possibility to convert this compound into a successful clinical drug. Therefore, significant attention has been paid to designing curcumin analogs by modifying their chemical structures to overcome the considerable drawbacks of curcumin and improve their pharmacological properties [14]. To date, a large number of studies have shed light on the high potential of bioactive piperidone molecules structurally related to curcumin as antitumor agents [15,16,17,18]. Piperidones are easily amenable to various chemical modifications, which makes this class of compounds promising as basic platforms for creating hybrid structures. A promising direction for creating such hybrids is the introduction of natural products into their composition [19,20,21].

Natural compounds have been the main sources of efficient drugs to design therapeutic strategies for the treatment of malignant neoplasms for many centuries. In particular, sesquiterpene lactones, which are a group of secondary metabolites obtained from the *Asteráceae* family [22], exhibit antitumor potential according to numerous reports [23,24,25]. Thus, a wide spectrum of biological activities has been convincingly demonstrated for costunolide [26,27,28], a sesquiterpene lactone isolated from the roots of *Saussureacostus*, dehydrocostuslactone [29,30] from the roots of *Saussurealappa*, alantolactone [31,32,33] from the roots of Inula helenium, and many others [34,35]. Owing to their participation in the modulation of different intracellular signaling pathways responsible for tumor growth and progression, these molecules can amplify the therapeutic potential and thus open new possibilities to improve chemotherapeutic treatment in the nearest future. 

**Figure 1:**
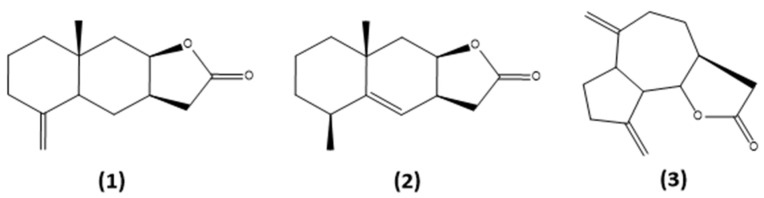
Molecular structures of natural sesquiterpene lactones: isoalantolactone (**1**), alantolactone (**2**), and dehydrocostus lactone (**3**).

Thus, considering the factors mentioned above to search for compounds with higher antitumor activity and specificity toward tumor cells, we performed the purposeful synthesis of new curcumin analogs, including conjugates of 3,5-bisarylidene-4-piperidone and modified sesquiterpene lactones, namely isoalantolactone (**1**, Figure 1), alantolactone (**2**, Figure 1), and dehydrocostus lactone (**3**, Figure 1). We also studied the possible mechanisms of their action using in vitro and in silico molecular screening methods. 

Thus, to date, the therapeutic potential of isoalantolactone has been convincingly demonstrated in the treatment of bladder [36] and endometrial cancer [37], colorectal carcinoma [38], liver cancer [39], breast cancer [40], etc. [41,42], due to its influence on a wide range of signaling pathways and pathological cascades that play a critical role in the progression of malignant neoplasms. Similarly well-known is the fact that alantolactone can exert an antitumor effect by targeting the pathways of apoptosis via Wnt/β-Catenin signaling [43], Nrf2 signaling pathway [44], and by regulating p38MAPK, NF-kB [45], and MAPK-JNK/c-Jun [46] pathways. The ability of dehydrocoslactone to suppress cancer progression by inhibiting migration [47], proliferation, epithelial–mesynchemic transition [48], sub-G1 cell cycle arrest, DNA damage, and loss of mitochondrial membrane potential [49] has been widely described.

## 2. Results and Discussion

### 2.1. Chemistry

**Scheme 1:**
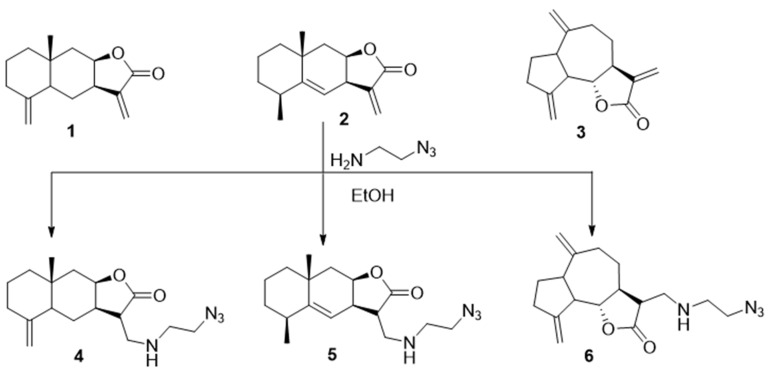
By introducing an azide group into sesquiterpene lactones **1**–**3**, compounds **4**–**6** were synthesized.

To solve the task of designing hybrid molecular systems via the conjugation of 3,5-bis(arylidene)-4-piperidones and sesquiterpene lactones (**1**–**3**), we used the click-chemistry methodology proposed by Professor Sharpless, which is widely used for preparing biologically active conjugates [50,51] and was previously used by us to construct hybrid molecules [52,53,54]. This approach was used because it is simple and efficient, and it is based on the [2+3] cycloaddition reaction, leading to the formation of 1,2,3-triazole hydrophilic linker that combines 3,5-bis(arylidene)-4-piperidones and sesquiterpene lactones into a single molecular system and serves as an important pharmacophore involved in binding to the target [55]. To implement the click-chemistry methodology, one should first introduce terminal acetylene bonds and N_3_ groups into the molecules selected for conjugation. To introduce N_3_ groups into sesquiterpene lactones **1**–**3**, we performed a nucleophilic addition reaction of 2-azidoethylamine at the terminal double bond located in the lactone ring. The reaction was performed in EtOH for 48 h. The yields of the azide blocks (**4**–**6**) were about 80% (Scheme 1).

**Scheme 2:**
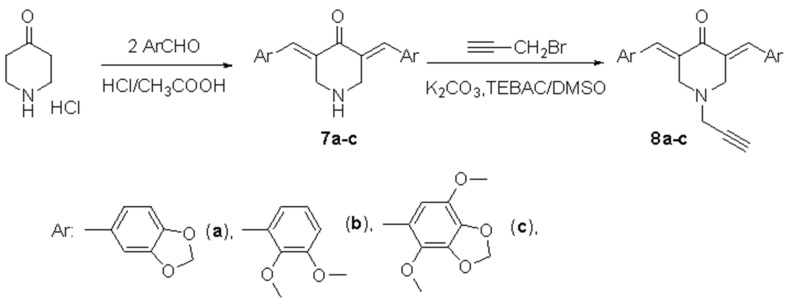
Synthesis of N-propargyl-3,5-bis(arylidene)piperidin-4-ones (**8a**–**c**).

To synthesize the acetylene fragment, we used 3,5-bis(benzylidene)-4-piperidones **7a**–**c**, which were obtained by the previously described procedure [56] (Scheme 2). Using propargyl bromide as an alkylating agent in the presence of K_2_CO_3_ and using DMSO as a solvent, we performed the N-alkylation of 3,5-bis(benzylidene)-4-piperidones **7a**–**c**. The yields of compounds **8a**–**c** were 82–95%. Azide (**4**–**6**) and acetylene (**8a**–**c**) compounds were isolated in individual states using column chromatography and were characterized using spectral methods (NMR and IR, detail in Supplementary Materials).

**Scheme 3:**
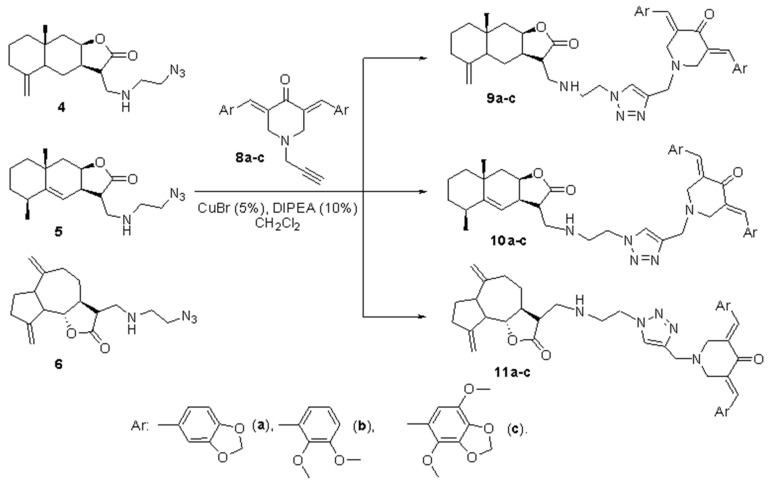
Conjugation of azides **4**–**6** with N-propargyl-3,5-bis(arylidene)piperidin-4-ones (**8a**–**c**).

The final stage of designing the molecular systems consisted of the conjugation of acetylene blocks **8a**–**c** with azide blocks **4**–**6** using CuBr as a catalyst. We used similar conditions previously for designing biologically hybrid active compounds based on natural substrates [52,53,54]. Target hybrid compounds (**9a**–**c**–**11a**–**c**) were isolated in 57–65% yields using column chromatography and characterized by spectral methods (Scheme 3).

### 2.2. Biological Evaluation

#### 2.2.1. Conjugates of Sesquiterpene Lactones and 3,5-Bis(arylidene)piperidin-4-ones Decrease Vitality of Tumor Cells

Initially, all studied compounds were tested for cytotoxicity against the human cells of tumor origin, including epithelial cells of breast adenocarcinoma MCF-7, neuroblastomes SH-SY5Y and IMR-32, human cervical adenocarcinoma HeLa, and conditionally normal culture of fibroblasts WI-38 obtained from fetal lung tissue.

The structural analogs considered as reference ligands were Arglabine, a sesquiterpene lactone isolated from *Artemisia glabella* and used in clinical practice as an antitumor and radiosensitizing agent [57], and curcumin, whose therapeutic potential as a modulator of carcinogenesis is widely described in the literature [58].

As shown in Table 1, the initial sesquiterpene lactones **1**–**3** and piperidones **7a**–**c** exhibit moderate cytotoxic activity toward the cell lines used, which agrees well with the known literature data [59,60,61] and confirms the reliability of the obtained results.

In a study of the cytotoxic profile of conjugates **9a**–**11c** containing the above piperidones and sesquiterpene lactones in their structure for a series of tumor cells, we revealed that these compounds retained high cytotoxicity toward cell lines obtained from different human solid malignancies, including two morphologically and biochemically different cell types of neuroblastomes SH-SY5Y and IMR-32, human cervical adenocarcinoma HeLa, and mammary duct adenocarcinoma MCF-7. So, the IC_50_ values of cytotoxic effect for the most toxic compound **11b** containing fragments of **3** and **7b** ranged from 6.07 ± 0.06 µM to 8.07 ± 0.02 µM, which is higher than the toxicity of the initial dehydrocostus lactone **3** (in IMR-32) and 3,5-bis(arylidene)piperidin-4-one **7b** (in HeLa and IMR-32). It should be noted that the cytotoxicity of the synthesized compounds in most cases exceeded that of the reference ligands. 

The results obtained for the normal cell line, WI-38, are of special interest. In contrast to the initial piperidones, the cytotoxicity of the synthesized conjugates toward WI-38 was considerably lower compared with that of the tumor cell lines. We calculated the selectivity indexes of each molecule, and Table 1 shows values equal to or greater than 3. Thus, the calculation of the selectivity index showed that it reached 7 for compound **11b**, whereas the IC_50_ value of the cytotoxic effect in WI-38 for **7b**—one of the pharmacophoric fragments of this conjugate—was in the submicromolar range. Selective action was reached because of the chemical modification of the initial piperidones by sesquiterpene lactones.

Thus, these results indicate the useful therapeutic window of the obtained conjugates and allow one to make intriguing assumptions regarding the design of agents specific to tumor cells. They also attract interest in further studying the mechanism of their action.

#### 2.2.2. Conjugates of Sesquiterpene Lactones and 3,5-Bis(arylidene)piperidin-4-ones Behave as Negative Regulators of Aerobic Glycolysis in Cells of Human Cervical Carcinoma

The metabolic profile of tumor cells differs from that of normal cells due to intense aerobic glycolysis, known as the Warburg effect [62,63]. This modification of metabolic patterns causes invasive behavior in tumors, including proliferation, metastasis, immunosuppression, drug resistance, and recurrence [64,65]. Thus, Liu and coauthors revealed a direct correlation between the Warburg effect and the progression of triple-negative breast cancer [66]. Glycolysis is also the dominant energy metabolism in epithelial ovarian carcinoma [67], lung cancer [68,69], colorectal carcinoma [70,71], brain tumors [72], and others. To date, convincing data have been accumulated, proving that targeting this reprogrammed metabolism in neoplastic cells can have a wide spectrum of positive results for the treatment of malignant neoplasms. In particular, a recent study by Jin et al. showed that dihydroartemisinin, which is an inhibitor of the allosteric glycolytic enzyme pyruvate kinase M2, amplifies the antitumor effects of photodynamic therapy in esophageal cancer cells [73]. In spite of the fact that glycolysis inhibitors are not allowed in clinical practice till now, changes in the energetic metabolism of tumor cells are the subject of intense studies by many research teams, which enables one to consider it as a promising and efficient approach to cancer treatment. 

In our study, to assess metabolic changes under the action of the prepared compounds at a concentration of 100 µM, we measured the acidification rate of the extracellular medium by human cervical adenocarcinoma HeLa cells using a glycolysis stress test. As shown in Table 2, the treatment of cells with the studied compounds considerably decreased the rate of extracellular acidification of the medium by cells, thereby reducing the glycolytic activity of tumor cells.

The most promising glycolysis inhibition profile was demonstrated for conjugate **11b**, containing **7b** and **3** fragments. Thus, compound **11b** reliably decreased the extracellular acidification rate in terms of glycolysis (by 34.30%), glycolytic capacity (by 56.40%), and glycolytic reserve (91.79%), which indicates the ability of this compound to drastically disturb the glycolytic metabolism of tumor cells, thus initiating a series of fatal events in neoplastic cells.

It should be noted that the parameters of glycolytic function calculated for cells treated with sesquiterpene lactones **1**–**3** (Table 2) showed no considerable difference from those for control samples, whereas glycolytic capacity and glycolytic reserve considerably decreased under the action of the initial piperidones **7a**–**c**.

**Figure 2:**
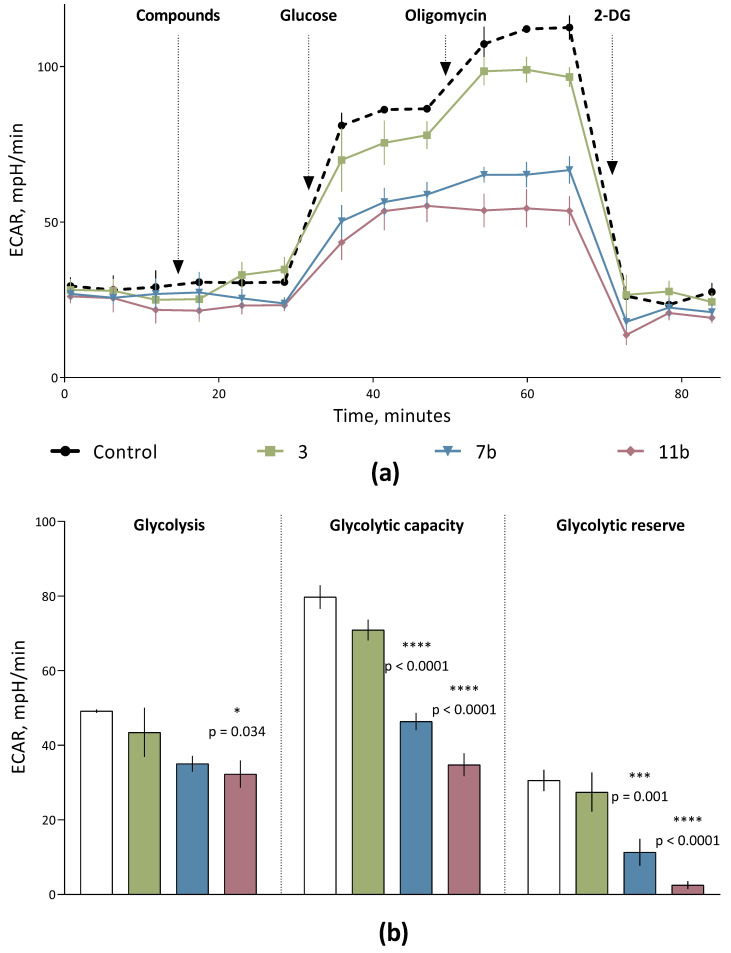
External cell acidification rate of the medium (ECAR) by HeLa cells measured in glycolysis stress test using a Seahorse XF-96 cell metabolism analyzer. (**a**) Kinetic curves for changes in external cell acidification rate of the medium by HeLa cells after sequential injection of the studied compounds (100 µM), glucose (10 mM), oligomycin (1 µM), and 2-deoxyglucose (2-DG) (50 µM). (**b**) Glycolytic function parameters: glycolysis represents the difference between the highest ECAR value before oligomycin injection and the last ECAR measurement after injection of the studied compounds; glycolytic capacity is calculated as the largest ECAR value after oligomycin injection minus the last ECAR measurement after injection of the studied compounds; glycolytic reserve is the difference between glycolytic capacity and glycolysis. The data are presented as Mean ± SEM, n = 3. *, ***, and ****—*p* < 0.05, *p* < 0.001, and *p* < 0.0001, respectively, in comparison with control (by 1-way ANOVA with Dunnett’s test).

Figure 2 provides representative images of the kinetic curves showing the change in the external cell acidification rate of the medium by HeLa cells, illustrating the actions of compounds **3** and **7b**, as well as their conjugate **11b**. Thus, based on the noted features, the obtained results allow us to suppose the key contribution of the piperidone platform to the emergence of glycolysis-inhibiting properties; however, modification with lactones seems to amplify this effect. 

#### 2.2.3. Determination of Allosteric Glycolytic Enzymes Binding Affinities of Compounds by Molecular Docking Analysis

To elucidate the molecular background of the glycolysis-inhibiting action of the studied compounds, we performed a molecular docking analysis for modeling the affinity of compounds binding to key enzymes of this process: hexokinase, phosphofructokinase, and pyruvate kinase M2.

The obtained docking scores for each compound in the binding sites of the rate-limiting enzymes of the glycolytic pathway are presented in Table 3. Thus, the study of molecular docking showed a larger binding affinity between the synthesized conjugates and pyruvate kinase M2, an enzyme that catalyzes the reaction in the final stage of the glycolytic pathway, transforming phosphoenolpyruvate into pyruvate. This is evidenced by the lowest values of binding affinity ranging from −8.4 to −9.9, thus indicating the formation of strong interactions with the target. It is interesting that the binding affinities of these potential inhibitors surpassed those of the positive control of phenylalanine.

It should also be noted that the initial piperidones showed the values of the estimated free energy of binding to hexokinase 2 and 6-phosphofructo-2-kinase similar to those of reference agents and, therefore, lower than for the synthesized conjugates (Table 3). This fact enables us to assume that the modification of the piperidone platform by sesquiterpene lactones hampers the entrance of the compounds into the binding sites of these enzymes.

**Figure 3:**
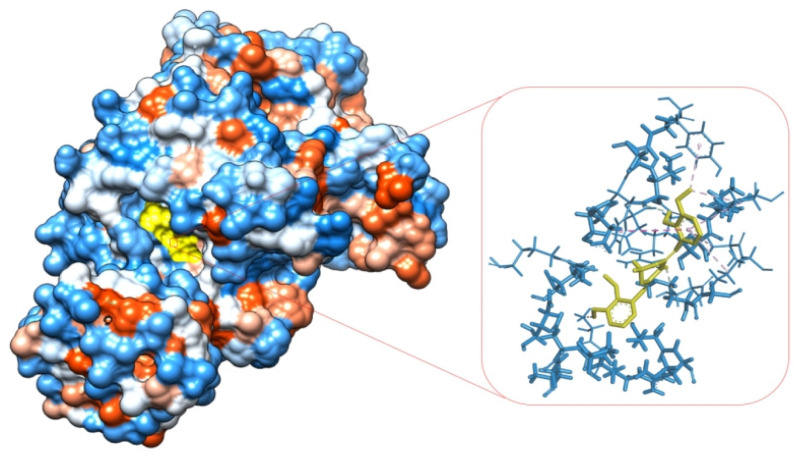
Representative image of the PKM2 protein–ligand complexes based on the example of **7b**, reflecting the binding site of the ligand in the crystal structure of the enzyme. The yellow sticks represent compound **7b**. The blue sticks represent the PKM2 structure.

In a detailed study of the mechanism of interaction of the studied compounds with PKM2, the docking of compounds showed that all synthesized conjugates exhibited good theoretical binding affinity to the target protein through the phosphoenolpyruvate (PEP)-binding site of this enzyme (Figure 3) via hydrogen bonding (Table 4) and hydrophobic (Table 5) and electrostatic (Table 6) interactions.

Table 4, Table 5 and Table 6 display the details of the interaction between the compounds and amino acid residues of the PEP-binding site of PKM2. Thus, it was revealed that all studied compounds produced hydrogen bonds of the conventional type (Table 4), mainly with the amino acids Arg73 and Lys270. It is of interest that, except for the initial lactones, both piperidones and their conjugates produce additional hydrogen bonds of the carbon type, while the structural features of **11a**, **11b**, and **11c** enable these compounds to interact with amino acids Asn75 and Asp296 via π-donor hydrogen bonds and salt bridges, thus improving the inhibition profile of the compound.

**Table 4:** Assessment of hydrogen bonding of the studied compounds in the PEP-binding site of PKM2.

	Hydrogen Bond							
	Conventional		Carbon		Pi-Donor Hydrogen Bond		Salt Bridge	
	Res.	Dis.	Res.	Dis.	Res.	Dis.	Res.	Dis.
**1**	ARG73	2.43						
**2**	LYS270	2.92						
**3**	ARG73	2.89						
		2.27						
	LYS270	2.79						
		2.80						
**7a**	ARG73	2.43	HIS84	2.83				
			SER362	2.31				
**7b**	ARG73 GLN329	2.53 2.36	SER362	2.34				
			THR328	3.75				
			ILE51	3.50				
**7c**	ARG73	2.53	SER362	2.57				
			ASN75	3.57				
**9a**	ARG73	2.48	SER362	2.83				
	ARG120	3.02						
	GLN329	2.08						
**9b**	ARG73	2.45	SER362	2.72				
			ALA293	3.76				
		2.04	ASN75	3.26				
			HIS78	3.44				
**9c**	ARG73	2.83	ASN75	3.46				
	LYS270	2.41	GLU118	3.76				
		2.57	ASP296	3.62				
**10a**	ARG120	2.98	GLY79	2.38				
**10b**	ARG294	2.82	LEU180	2.89				
		2.98	GLY298	3.62				
		2.45	GLN329	3.16				
	ALA303	2.21	ASP177	3.65				
**10c**	LYS270	2.51	GLY79	2.58				
		2.29	HIS84	2.95				
		2.93	HIS78	3.78				
	SER362	2.76	GLU118	3.66				
			SER243	3.55				
**11a**	ARG73	2.32	ASP296	3.27	ASN75	2.85		
	ASN75	2.44		2.95				
**11b**	ARG73	2.72	GLU118	3.27			ASP296	2.44
								2.27
**11c**	SER77	2.47	GLY79	2.32	ASN75	3.11		
	LYS207	2.21	HIS78	3.73				
	LYS270	2.20	GLU118	3.62				
			SER243	3.48				

The analysis of hydrogen bonding of the synthesized compounds with the PEP-binding site of PKM2 revealed that both the initial piperidones and their conjugates mainly formed π-alkyl and alkyl types of bonds (Table 5); Pro53, Ala366, Ala293, and Lys367 are typical amino acids that produce this kind of interaction with the majority of the docked ligands. Furthermore, compounds **9a**,**b**, **10a**,**b**, and **11b** showed amide-π stacked, π–π stacked, or π-sigma interactions with His78, Tyr83, Tyr175, and Gly298 amino acid residues, which amplified the PKM2-inhibiting action of these compounds.

**Table 5:** Assessment of the hydrophobic interactions of the studied compounds in the PEP-binding site of PKM2.

	Hydrophobic Interactions									
	Pi-Pi T-Shaped		Pi-Alkyl/Alkyl		Amide-Pi Stacked		Pi-Pi Stacked		Pi-Sigma	
	Res.	Dis.	Res.	Dis.	Res.	Dis.	Res.	Dis.	Res.	Dis.
**1**	No electrostatic interactions									
**2**										
**3**										
**7a**	HIS78	5.70	TYR83	4.32						
			HIS84	4.65						
			ALA366	4.73						
**7b**			TYR83	4.73						
			HIS84	5.31						
			PRO53	4.96						
			ALA36	4.71						
			LYS367	5.37						
			ALA293	4.47						
			PRO53	4.52						
			LYS367	4.06						
**7c**	HIS78	5.80	TYR83	4.42						
			PRO53	4.91						
			ALA366	4.70						
			LYS367	4.98						
			ALA293	3.60						
			PRO53	4.29						
**9a**			TYR83	4.57	HIS78	5.25				
			ALA366	4.25						
**9b**			HIS78	4.45			HIS78	4.64		
			TYR83	4.75						
			PRO53	5.09						
				4.31						
			ALA366	4.56						
			LYS367	4.64						
			ALA293	3.17						
**9c**			HIS78	4.96						
			LYS367	5.33						
			PRO53	4.61						
			ALA293	3.67						
			ALA366	5.48						
				4.86						
			MET291	4.98						
**10a**			ALA366	4.23			HIS78	3.97		
			LYS367	5.35			TYR83	3.91		
**10b**			ALA303	5.47			TYR175	4.89	GLY298	2.79
				4.91						
			ILE299	5.16						
			LEU180	4.13						
			ALA343	4.26						
			PRO302	4.67						
**10c**			ALA366	4.62						
			LYS36	5.48						
			PRO53	5.31						
				4.54						
				3.79						
			LYS367	3.87						
**11a**			HIS78	4.80						
			ALA366	4.11						
			LEU180	4.18						
			ILE299	5.35						
**11b**			HIS78	4.94			HIS78	5.20	ASP178	3.88
			TYR83	4.71						
			HIS84	4.58						
			ALA366	4.64						
**11c**			HIS78	5.05						
				4.40						
			ALA366	4.32						
			PRO53	5.34						
			ALA293	4.43						
			LYS367	4.86						

The assessment of electrostatic interactions for the synthesized compounds with the PEP-binding site of PKM2 showed the presence of the maximum number of bonds of this kind for compound **11b** (Table 6), which contributed considerably to the most preferable bond energy equal to −9.9 kcal/mol. In addition to hydrogen bonding and hydrophobic interactions, **11b** actively forms π-cation and attractive charge bonds, thus producing the most stable ligand–target complex in terms of orientation and conformation.

**Table 6:** Assessment of electrostatic interactions of the synthesized compounds in the PEP-binding site of PKM2.

	Electrostatic Interactions					
	Pi-Anion		Pi-Cation		Attractive Charge	
	Res.	Dis.	Res.	Dis.	Res.	Dis.
**1**	No electrostatic interactions					
**2**						
**3**						
**7a**						
**7b**						
**7c**						
**9a**	GLU332	4.04				
**9b**			ARG120	4.27		
**9c**	ASP296	3.64				
	GLU332					
**10a**	No electrostatic interactions					
**10b**	No electrostatic interactions					
**10c**			LYS207	3.59		
**11a**	GLU118	4.38	ARG120	3.20		
**11b**			ARG120	3.97	GLU118	5.34
					GLU272	4.13
**11c**	ASP296	4.47				

**Figure 4:**
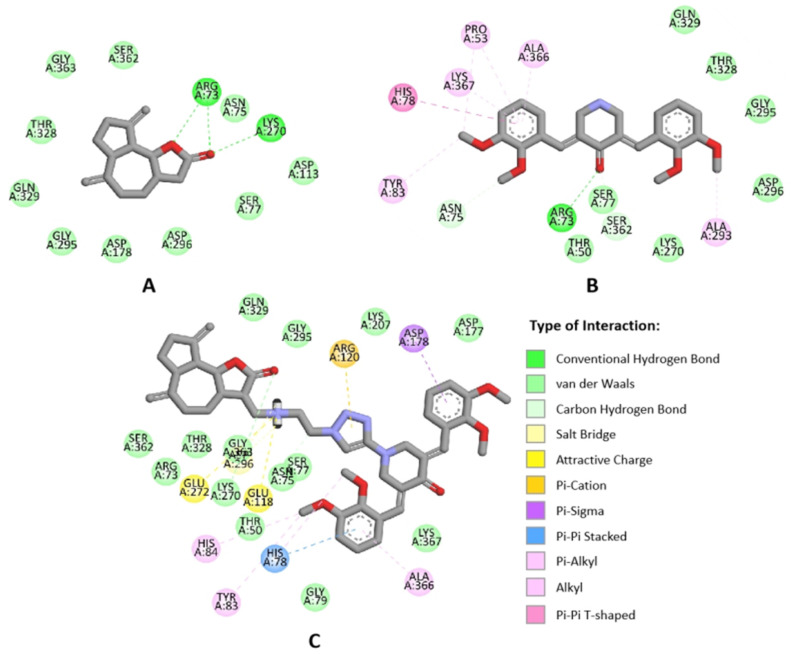
(**A**–**C**) Docking poses of **3**, **7b**, and **11b** with target protein by pyruvate kinase M2 (PDB ID: 6V74). Nitrogen and oxygen atoms are marked blue and red, respectively.

Two-dimensional representations of the favorable binding modes of conjugates **7b** and **3**–**11b** and their initial fragments are demonstrated in Figure 4. It is seen that **11b** displays a much wider spectrum of docking interaction patterns against the PEP-binding site of PKM2, which causes a much lower binding affinity of enzyme −9.9 at docking scores of −6.1 and −7.9 for dehydrocostus lactone **3** and 3,5-bis(arylidene)piperidin-4-one **7b**, respectively.

As a whole, the above results allow us to assume that modulation of glycolytic function by the studied derivatives may be caused by the presence of unique sites in their structures for binding to pyruvate kinase M2, which directly affects the activity of this enzyme and, therefore, the glycolysis process at large.

Thus, the obtained data on the selective interaction of the synthesized conjugates (but not the initial 3,5-bis(arylidene)piperidin-4-ones) with pyruvate kinase M2 led to the formulation of a hypothesis on the possible contribution of this phenomenon to the selectivity of the cytotoxic action of compounds, which was revealed in the study of their effect on the survival of cells of both tumor and normal origin. To date, it is well established that the second isoform of pyruvate kinase, which is a highly specific tumor protein, participates in the development of malignant neoplasms and, therefore, this enzyme refers to the class of metabolic oncomarkers [74]. It is of interest that there is no PKM2 in healthy adult organisms, but instead, three others are in operation (mainly PKM1). So, the blood serum samples of patients with primary prostate cancer exhibit a direct correlation between the increased expression of subtype 2 of pyruvate kinase and bone metastasis [75,76]. Analysis of tissues obtained from patients with stomach cancer and glioblastoma revealed an increase in the content of this enzyme associated with low survivability [77,78], which emphasizes the clinical significance of PKM2—an enzyme that supports the divergent biosynthetic and energetic needs of tumor cells—in the pathogenesis of different types of malignant neoplasms. Moreover, it was convincingly proven that the inhibition of PKM2 in the therapy of triple-negative breast cancer has no negative effect on normal tissues, which supports colossal interest in revealing the inhibitors of this enzyme [79]. Therefore, the decrease in cytotoxic activity of the synthesized derivatives toward normal cells can be caused by their structural features, allowing a direct influence on pyruvate kinase M2 without affecting other key enzymes of glycolysis universally expressed in normal tissues.

## 3. Materials and Methods

### 3.1. Reagents and Materials

All commercial reagents were used as purchased without further purification; all solvents used in the reactions were freshly distilled from appropriate drying agents before use. Analytical TLC was performed on Merck silica gel 60 F_254_ plates (Darmstadt, Germany), visualized under UV light (λ_max_ = 254 nm) or by staining with iodine vapor. Column chromatography was carried out using Merck silica gel (Kieselgel 60, 0.063–0.200 mm, Darmstadt, Germany). The ^1^H, and ^13^C spectra were recorded on a Bruker Avance 400 spectrometer operating at 400.1 and 100.6, respectively. The chemical shifts (δ) are reported in ppm using residual (^1^H) or deuterated (^13^C) solvent signals as an internal standard rel. to TMS. The ^13^C NMR spectra were registered using the JMODECHO mode; the signals for the C-atom bearing odd and even numbers of H-atoms have opposite polarities. IR spectra were recorded in film or KBr pellets on a Fourier-spectrometer “Magna-IR750” (Nicolet, Glendale, WI, USA), with a resolution of 2 cm^−1^ and 128 scans. Analytical data (C, H, and N content) were obtained using a Carlo Erba model 1106 microanalyzer. High-resolution mass spectra (HRMS) were recorded on a Bruker micro TOF II instrument using electrospray ionization (ESI). The measurements were performed in a positive ion mode (interface capillary voltage: 4500 V); the mass ranged from *m*/*z* 50 to 3000; external or internal calibration was carried out using ESI Tuning Mix, Agilent (Waldbronn, Germany). A syringe injection was used for solutions in MeCN (flow rate 4 μL/min). N_2_ was applied as a dry gas; the interface temperature was set at 180 or 200 °C. HRMS were recorded at the Department of Structural Studies of Zelinsky Institute of Organic Chemistry, Moscow.

Isoalantolactone **1**, alantolactone **2** [80], and dehydrocostus lactone **3 [81]** were isolated from plant substrates according to previously reported procedures.

### 3.2. General Procedure for the Synthesis of Azides ***4***–***6*** [52]

To a solution of appropriate lactone (1 mmol, 1.0 eq) in EtOH (8 mL), 1-amino-2-azidoethane (2 mmol, 2.0 eq) was added. The mixture was stirred for 48 h at room temperature. After EtOH evaporation, the residue was dissolved in CH_2_Cl_2_ (10 mL). The solvent was removed in vacuo, affording azide **4** as a pale-yellow powder. In the case of azides **5**, **6**, the solvent was evaporated to afford azides (quant) as a viscous yellow oil, which were used for the next step without further purification.

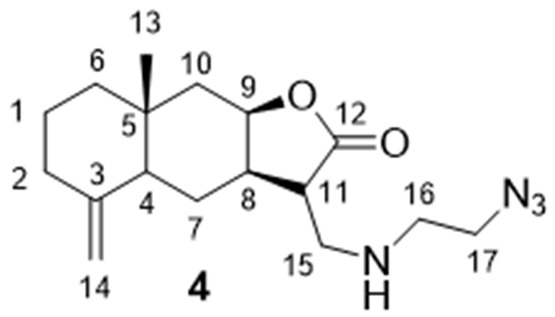

*(3S*,*3aR*,*8aR*,*9aR)-3-(((2-azidoethyl)amino)methyl)-8a-methyl-5-methylenedecahydronaphtho[2*,*3-b]furan-2(3H)-one* (**4**), Pale-yellow powder (83%), m.p. 101–102 °C. ^1^H NMR (400 MHz, CDCl_3_) δ 4.79 (1H, br.s, H-14a), 4.51 (1H, br.s, H-9), 4.47 (1H, br.s, H-14b), 3.43 (2H, t, *J* = 5.6 Hz, H_2_-17), 3.38 (1H, br.s, NH), 3.06 (1H, dd, *J* = 11.6 Hz, *J* = 6.8 Hz, H-15a), 2.95–2.75 (4H, m, H-15b, H_2_-16, H-8), 2.52 (1H, ddd, *J* = 12.7 Hz, *J* = 6.0 Hz, *J* = 2.0 Hz, H-11), 2.33 (1H, d, *J* = 12.4 Hz, H-2a), 2.18 (1H, d, *J* = 15.4 Hz, H-10a), 2.04–1.96 (1H, m, H-2b), 1.80 (1H, d, *J* = 12.1 Hz, H-6a), 1.70–1.45 (6H, m, H-1, H-4, H-7a, H-10b), 1.28–1.22 (2H, m, H-6b, H-7b), and 0.82 (3H, s, H_3_-13). ^13^C NMR (100 MHz, CDCl_3_) δ 177.85 (C-12), 149.13 (C-3), 106.33 (C-14), 78.20 (C-9), 51.06 (C-17), 48.63 (C-15), 47.42 (C-11), 46.33 (C-4), 44.92 (C-16), 42.07 (C-6), 41.23 (C-10), 38.90 (C-4), 36.59 (C-2), 34.65 (C-5), 22.52 (C-7), 20.91 (C-1), and 17.66 (C-13). IR (KBr) *v*_max_ 2932, 2866, 2096 (N_3_), 1747 (C=O), 1643, 1447, 1297, 1158, 963, and 894 cm^−1^. HRMS (ESI): *m*/*z* calcd. for C_17_H_27_N_4_O_2_ [M + H]^+^ 319.2129, found 319.2135. Anal. Calc. for C_17_H_26_N_4_O_2_ × 0.1 CH_2_Cl_2_: C, 62.83; H, 8.08; N, 17.14%. Found: C, 62.85; H, 8.02; N, 17.20%.

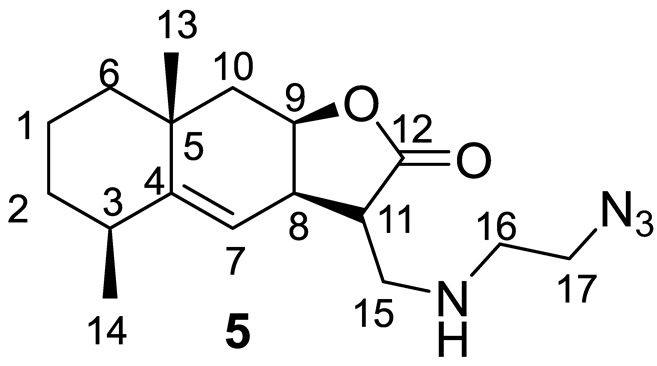

*(3S*,*3aR*,*5S*,*8aR*,*9aR)-3-(((2-Azidoethyl)amino)methyl)-5*,*8a-dimethyl-3*,*3a*,*6*,*7*,*8*,*8a*,*9*,*9aoctahydronaphtho[2*,*3-b]furan-2(5H)-one* (**5**), Yellow oil (85%). ^1^H NMR (400 MHz, CDCl_3_) δ 5.15 (1H, d, *J* = 2.8 Hz, H-7), 4.76 (1H, m, H-9), 3.44 (2H, d, *J* = 5.6 Hz, H_2_-17), 3.16–3.13 (1H, m, H-8), 3.02–2.78 (5H, m, H-11, H_2_-15, H_2_-16), 2.50–2.47 (1H, m, H-3), 2.11 (1H, dd, *J* = 14.6 Hz, *J* = 3.1 Hz, H-10a), 1.87–1.76 (1H, m, H-2a), 1.61–1.42 (6H, m, H-1, H-2b, H-6, H-10b), 1.23 (3H, s, H_3_-13), and 1.12 (3H, d, *J* = 7.6 Hz, H_3_-14). ^13^C NMR (100 MHz, CDCl_3_) δ 177.12 (C-12), 150.10 (C-4), 114.58 (C-7), 76.69 (C-9), 50.44 (C-17), 47.93 (C-15), 45.54 (C-16), 45.02 (C-11), 42.04 (C-10), 41.50 (C-6), 37.77 (C-3), 36.92 (C-8), 32.31 (C-5), 32.15 (C-2), 27.99 (C-13), 22.32 (C-14), and 16.18 (C-1). IR (KBr) *ν*_max_ 2929, 2101, 1762, 1457, 1340, 1180, 1151, 1039, and 733 cm^−1^. Anal. Calc. for C_17_H_26_N_4_O_2_·0.15 CH_2_Cl_2_: C, 62.20; H, 8.00; N, 16.92%. Found: C, 62.68; H, 8.03; N, 16.80%.

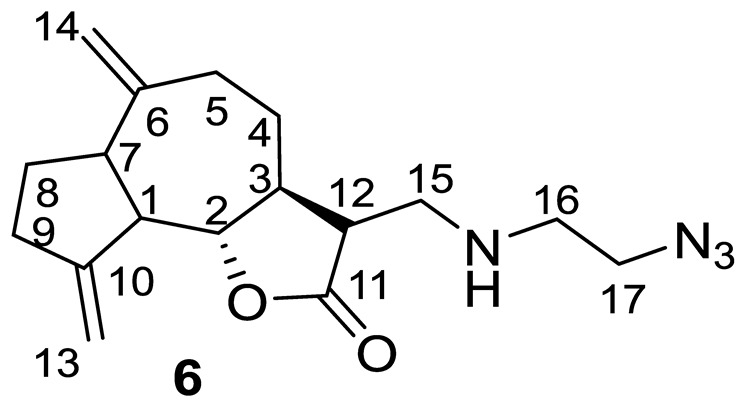

*(3R*,*3aS*,*9bS)-3-(((2-Azidoethyl)amino)methyl)-6*,*9-dimethylenedecahydroazuleno[4*,*5-b]furan-2(9bH)-one* (**6**), Yellow oil (82%). ^1^H NMR (400 MHz, CDCl_3_) δ 5.30 (1H, s, H-13a), 5.17 (1H, s, H-13b), 4.87 (1H, s, H-14a), 4.76 (1H, s, H-14b), 3.96 (1H, t, *J* = 9.1 Hz, H-2), 3.37 (2H, t, *J* = 5.6 Hz, H_2_-17), 2.97 (1H, dd, *J* = 12.1 Hz, *J* = 3.8 Hz, H-15a), 2.89–2.81 (4H, m, H-1, H-7, H-12, H-15b), 2.51–2.45 (3H, m, H-5a, H-9), 2.39–2.34 (2H, m, H_2_-16), 2.15–1.85 (5H, m, H-3, H-4a, H-5b, H-8), and 1.39–1.24 (1H, m, H-4b). ^13^C NMR (100 MHz, CDCl_3_) δ 177.51 (C-11), 151.56 (C-10), 149.65 (C-6), 111.64 (C-13), 108.85 (C-14), 85.62 (C-2), 51.64 (C-12), 50.96 (C-17), 48.82 (C-15), 47.30 (C-1), 47.11 (C-16), 46.74 (C-7), 44.57 (C-3), 37.46 (C-9), 32.40 (C-5), 32.33 (C-4), and 29.95 (C-8). IR (KBr) *ν*_max_ 2929, 2100, 1767, 1456, 1340, 1176, 1004, and 894 cm^−1^. Anal. Calc. for C_17_H_24_N_4_O_2_·0.1 CH_2_Cl_2_: C, 63.22; H, 7.51; N, 17.24%. Found: C, 63.15; H, 7.42; N, 17.09%.

### 3.3. General Procedure for the Synthesis of 3,5-Bis(arylidene)piperidin-4-ones (***7a***–***c***)

Piperidin-4-one hydrochloride (3.7 mmol, 1.0 eq) and appropriate aryl aldehyde (7.4 mmol, 2 eq) were mixed in glacial acetic acid (10 mL). Dry hydrogen chloride gas bubbled through the solution for 20 min. The reaction mixture was stirred for 2 h and was allowed to stay at room temperature overnight. After that, the acetic acid was evaporated, and the residue was treated with a saturated solution of sodium bicarbonate (7 g) in water (20 mL). The precipitate obtained was filtered off, washed with water (3 × 20 mL), and dried under vacuum. The resulting compounds **7a**–**c** were used for further interactions without additional purification.

Spectral data and melting points of the known compounds **7a** [82] and 7**b [83]** fit well the literature data.

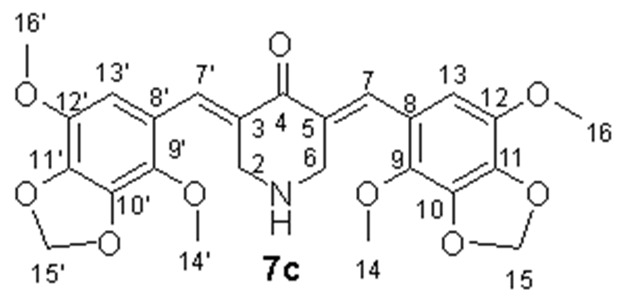

*(3E*,*5E)-3*,*5-Bis((4*,*7-dimethoxybenzo[d][1,3]dioxol-5-yl)methylene)piperidin-4-one* (**7c**), Yellow-green crystals (95%), m.p. 163–164 °C. ^1^H NMR (400 MHz, DMSO-d_6_) δ 7.68 (2H, s, H-7, H-7′), 6.52 (2H, s, H-13, H-13′), 6.09 (4H, s, H-15, H-15′), 3.92 (4H, s, H-2, H-6), and 3.88 and 3.83 (12H, both s, H_3_-14, H_3_-14′, H_3_-16, H_3_-16′). ^13^C NMR (100 MHz, DMSO-d_6_) δ 187.77 (C-4), 138.89 (C-11, C-11′), 138.81 (C-10, C-10′), 138.32 (C-12, C-12′), 137.71 (C-9, C-9′), 135.48 (C-7, C-7′), 129.18 (C-3, C-5), 121.25 (C-8, C-8′), 109.87 (C-13, C-13′), 102.58 (C-15, C-15′), 60.64 (C-14, C-14′), 57.07 (C-16, C-16′), and 48.08 (C-2, C-6). IR (KBr) *v*_max_ 2945, 2840, 1631, 1589, 1492, 1456, 1358, 1237, 1141, 1068, 958, and 521 cm^−1^. HRMS (ESI): *m*/*z* calcd. for C_25_H_26_NO_9_ [M + H]^+^ 484.1602, found 484.1614. Anal. Calc. for C_25_H_25_NO_9_ × 0.35 H_2_O: C, 61.31; H, 5.29; N, 2.86%. Found: C, 61.23; H, 5.48; N, 2.88%.

### 3.4. General Procedure for the Synthesis of N-Propargyl-3,5-bis(arylidene)piperidin-4-ones (***8a***–***c***)

To a stirred mixture of 3,5-bis(arylidene)piperidin-4-one (0.01 mol, 1.0 eq) and potassium carbonate (0.04 mol, 4.0 eq) in DMSO (15 mL), propargyl bromide (0.012 mol, 1.2 eq) was added. The solution obtained was stirred at room temperature for 5 min and then benzyl triethyl ammonium chloride (10 mg) was added. The resulting mixture was stirred for 2 h (TLC monitoring), and then water (100 mL) was added. The precipitate obtained was filtered off, washed on the filter with water, dried in air, and stored in vacuo over P_2_O_5_. Analytical grade samples **8a**–**c** were obtained via column chromatography with SiO_2_ using gradient systems of CH_2_Cl_2_–MeOH solvents (50:0→50:1).

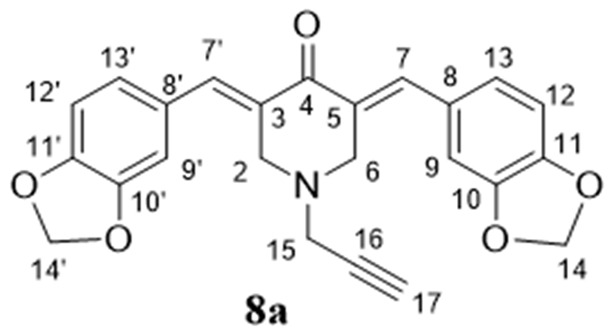

*(3E*,*5E)-3*,*5-Bis(benzo[d][1,3]dioxol-5-ylmethylene)-1-(prop-2-yn-1-yl)piperidin-4-one* (**8a**), Yellow crystals (82%), m.p. > 192 °C (decomp.). ^1^H NMR (400 MHz, CDCl_3_) δ 7.76 (2H, s, H-7, H-7′), 6.97 (2H, d, *J* = 8.0 Hz, H-12, H-12′), 6.91 (2H, s, H-9, H-9′), 6.90 (2H, d, *J* = 8.0 Hz, H-13, H-13′), 6.05 (4H, s H-14, H-14′), 3.93 (4H, s, H-2, H-6), 3.58 (2H, d, *J* = 1.8 Hz, H_2_-15), and 2.41 (1H, t, *J* = 1.8 Hz, H-17). ^13^C NMR (100 MHz, CDCl_3_) δ 186.16 (C-4), 148.42 (C-11, C-11′), 147.83 (C-10, C-10′), 136.70 (C-7, C-7′), 130.93 (C-8, C-8′), 129.19 (C-3, C-5), 125.95 (C-13, C-13′), 109.92 (C-12, C-12′), 108.55 (C-9, C-9′), 101.41 (C-14, C-14′), 77.12 (C-16), 74.89 (C-17), 53.22 (C-2, C-6), and 46.33 (C-15). HRMS (ESI): *m*/*z* calcd. for C_24_H_20_NO_5_ [M + H]^+^ 402.1336, found 402.1346. Anal. Calc. for C_24_H_19_NO_5_× 0.5 H_2_O: C, 70.23; H, 4.91; N, 3.41%. Found: C, 70.06; H, 4.81; N, 3.55%.

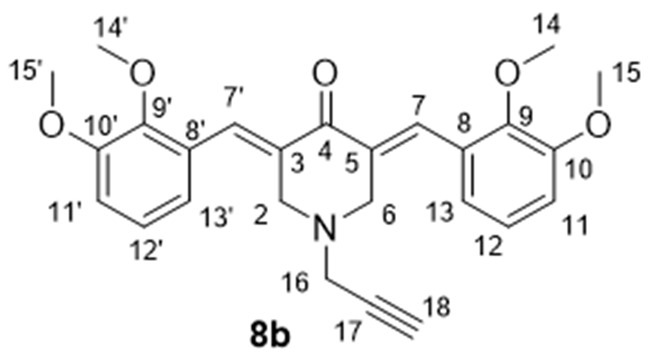

*3*,*5-Bis((E)-2*,*3-dimethoxybenzylidene)-1-(prop-2-yn-1-yl)piperidin-4-one* (**8b**), Yellow crystals (87%), m.p. 60–61 °C. ^1^H NMR (400 MHz, CDCl_3_) δ 8.07 (2H, s, H-7, H-7′), 7.11 (2H, t, *J* = 8.0 Hz, H-12, H-12′), 6.97 (2H, d, *J* = 8.0 Hz, H-11, H-11′), 6.87 (2H, d, *J* = 8.0 Hz, H-13, H-13′), 3.90 (6H, s, H_3_-15, H_3_-15′), 3.83 (10H, br.s, H_3_-14, H_3_-14′, H-2, H-6), 3.47 (2H, d, *J* = 1.6 Hz, H_2_-16), and 2.31 (1H, t, *J* = 1.6 Hz, H-18). ^13^C NMR (100 MHz, CDCl_3_) δ 186.26 (C-4), 152.79 (C-9, C-9′), 148.39 (C-10, C-10′), 133.43 (C-8, C-8′), 132.58 (C-7, C-7′), 129.41 (C-3, C-5), 123.61 (C-13, C-13′), 121.85 (C-12, C-12′), 113.12 (C-11, C-11′), 77.41 (C-17), 74.43 (C-18), 61.27 (C-16, C-16′), 55.72 (C-15, C-15′), 53.41 (C-2, C-6), and 46.13 (C-16). HRMS (ESI): *m*/*z* calcd. for C_26_H_28_NO_5_ [M + H]^+^ 434.1962, found 434.1971. Anal. Calc. for C_26_H_27_NO_5_ × 1.5 H_2_O: C, 68.54; H, 6.68; N, 3.04%. Found: C, 68.59; H, 6.31; N, 3.10%.

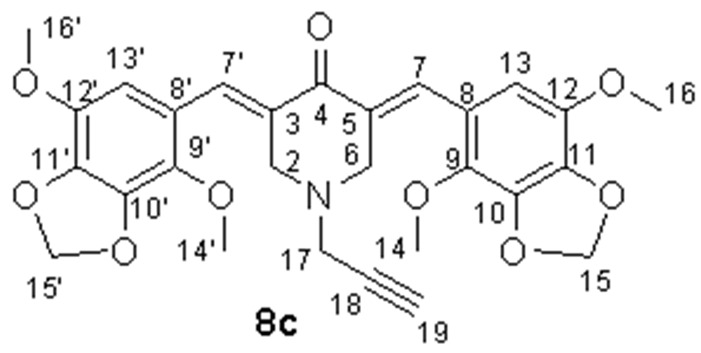

*(3E*,*5E)-3*,*5-Bis((4*,*7-dimethoxybenzo[d][1,3]dioxol-5-yl)methylene)-1-(prop-2-yn-1-yl)piperidin-4-one* (**8c**), Yellow crystals (95%), m.p. 167–168 °C. ^1^H NMR (400 MHz, CDCl_3_) δ 7.96 (2H, s, H-7, H-7′), 6.43 (2H, s, H-13, H-13′), 6.04 (4H, s, H-15, H-15′), 3.93 and 3.87 (12H, both s, H_3_-14, H_3_-14′, H_3_-16, H_3_-16′), 3.82 (4H, s, H-2, H-6), 3.49 (2H, d, *J* = 2.0 Hz, H_2_-17), and 2.31 (1H, t, *J* = 2.0 Hz, H-19). ^13^C NMR (100 MHz, CDCl_3_) δ 186.20 (C-4), 138.60 (C-11, C-11′), 138.57 (C-10, C-10′), 138.04 (C-12, C-12′), 137.89 (C-9, C-9′), 132.40 (C-3, C-5), 132.24 (C-7, C-7′), 121.44 (C-8, C-8′), 109.41 (C-13, C-13′), 101.92 (C-15, C-15′), 77.52 (C-18), 74.14 (C-19), 60.40 (C-14, C-14′), 56.92 (C-16, C-16′), 53.65 (C-2, C-6), and 46.25 (C-17). HRMS (ESI): *m*/*z* calcd. for C_28_H_28_NO_9_ [M + H]^+^ 522.1759, found 522.1765. Anal. Calc. for C_28_H_27_NO_9_ × 0.25 H_2_O: C, 63.93; H, 5.27; N, 2.66%. Found: C, 63.90; H, 5.29; N, 2.57%.

### 3.5. General Procedure for the “Click”-Reactions

To a stirred mixture of azide (0.2 mmol, 1.0 eq) and the corresponding alkyne (0.21 mmol, 1.05 eq.) in CH_2_Cl_2_ (5 mL), copper(I) bromide (0.01 mmol, 5 mol.%) and DIPEA (0.02 mmol, 10 mol.%) were added. The solution obtained was stirred at room temperature for 24 h (TLC monitoring). The solvent was removed in vacuo, and the remaining crude product was purified via column chromatography (dichloromethane/ethanol, 100:0.2 to 100:5) to afford the corresponding product as a viscous yellow oil.

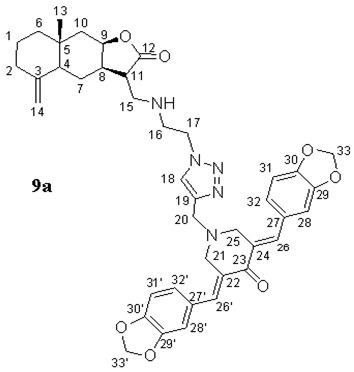

*(3E*,*5E)-3*,*5-bis(benzo[d][1,3]dioxol-5-ylmethylene)-1-((1-(2-((((3S*,*3aR*,*8aR*,*9aR)-8a-methyl-5-methylene-2-oxododecahydronaphtho[2*,*3-b]furan-3-yl)methyl)amino)ethyl)-1H-1*,*2*,*3-triazol-4-yl)methyl)piperidin-4-one* (**9a**), Yellow oil (68%). ^1^H NMR (400 MHz, CDCl_3_) δ 7.63 (2H, s, H-26, H-26′), 7.52 (1H, s, H-18), 6.88 (2H, d, *J* = 8.0 Hz, H-32, H-32′), 6.82–6.77 (4H, m, H-28, H-28′, H-31, H-31′), 5.97 (4H, s, H_2_-33, H_2_-33′), 4.72 (1H, s, H-14a), 4.38–4.35 (4H, m, H-9, H-14b, H_2_-17), 3.86–3.82 (6H, m, H_2_-20, H_2_-21, H_2_-25), 3.10–3.06 (1H, m, H-8), 2.99–2.95 (2H, m, H_2_-16), 2.78–2.73 and 2.70–2.63 (2H, both m, H_2_-15), 2.41–2.35 (1H, m, H-11), 2.28–2.25 (1H, m, H-2a), 2.10 (1H, d, *J* = 15.4 Hz, H-10a), 1.96–1.92 (1H, m, H-2b), 1.71 (1H, d, *J* = 12.0 Hz, H-6a), 1.55–1.42 (5H, m, H-1, H-4, H-7a, H-10b), 1.22–1.05 (2H, m, H-6b, H-7b), and 0.74 (3H, s, H_3_-13). ^13^C NMR (100 MHz, CDCl_3_) δ 186.72 (C-23), 177.69 (C-12), 148.93 (C-3), 148.21 (C-30, C-30′), 147.66 (C-29, C-29′), 143.86 (C-19), 135.84 (C-26, C-26′), 131.35 (C-27, C-27′), 129.02 (C-22, C-24), 125.67 (C-32, C-32′), 123.14 (C-18), 109.77 (C-31, C-31′), 108.34 (C-28, C-28′), 106.22 (C-14), 101.27 (C-33, C-33′), 78.05 (C-9), 54.33 (C-21, C-25), 52.25 (C-20), 49.76 (C-17), 48.92 (C-15), 47.02 (C-11), 46.10 (C-4), 44.63 (C-16), 41.86 (C-6), 41.08 (C-10), 38.70 (C-8), 36.41 (C-2), 34.47 (C-5), 22.37 (C-7), 20.75 (C-1), and 17.51 (C-13). IR (film) *v*_max_ 2930, 1761 (C=O), 1596, 1504, 1489, 1446, 1263, 1235, 1039, 931, and 756 cm^−1^. HRMS (ESI): *m*/*z* calcd. for C_41_H_46_N_5_O_7_ [M + H]^+^: 720.3397, found 720.3395.

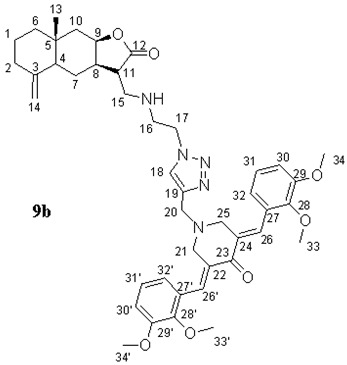

*(3E*,*5E)-3*,*5-bis(2*,*3-dimethoxybenzylidene)-1-((1-(2-((((3S*,*3aR*,*8aR*,*9aR)-8a-methyl-5-methylene-2-oxododecahydronaphtho[2*,*3-b]furan-3-yl)methyl)amino)ethyl)-1H-1*,*2*,*3-triazol-4-yl)methyl)piperidin-4-one* (**9b**), Yellow oil (62%). ^1^H NMR (400 MHz, CDCl_3_) δ 8.05 (2H, s, H-26, H-26′), 7.47 (1H, s, H-18), 7.08 (2H, t, *J* = 7.6 Hz, H-31, H-31′), 6.96 (2H, d, *J* = 8.0 Hz, H-30, H-30′), 6.81 (2H, d, *J* = 7.6 Hz, H-32, H-32′), 4.76 (1H, s, H-14a), 4.48–4.37 (4H, m, H-9, H-14b, H_2_-17), 3.90–3.82 (18H, m, H_3_-33, H_3_-33′, H_3_-34, H_3_-34′, H_2_-20, H_2_-21, H_2_-25), 3.10–3.05 (1H, m, H-8), 3.05–2.99 (2H, m, H_2_-16), 2.88–2.83 and 2.79–2.69 (2H, both m, H_2_-15), 2.50–2.40 (1H, m, H-2a), 2.33–2.30 (1H, m, H-11), 2.16 (1H, d, *J* = 15.6 Hz, H-10a), 2.00–1.95 (1H, m, H-2b), 1.75 (1H, d, *J* = 12.0 Hz, H-6a), 1.65–1.46 (5H, m, H-1, H-4, H-7a, H-10b), 1.30–1.15 (2H, m, H-6b, H-7b), and 0.80 (3H, s, H_3_-13). ^13^C NMR (100 MHz, CDCl_3_) δ 187.17 (C-23), 177.87 (C-12), 152.74 (C-3), 149.08 (C-28, C-28′), 148.24 (C-29, C-29′), 144.02 (C-19), 133.61 (C-22, C-24), 132.88 (C-26, C-26′), 129.44 (C-27, C-27′), 123.82 (C-30, C-30′), 123.30 (C-18), 122.01 (C-30, C-30′), 113.37 (C-32, C-32′), 106.49 (C-14), 78.47 (C-9), 61.28 (C-33, C-33′), 55.89 (C-34, C-34′), 54.37 (C-21, C-25), 51.61 (C-20), 49.60 (C-17), 49.09 (C-15), 46.38 (C-11), 46.24 (C-4), 44.91 (C-16), 42.17 (C-6), 41.35 (C-10), 38.98 (C-8), 36.70 (C-2), 34.47 (C-5), 22.67 (C-7), 21.06 (C-1), and 17.81 (C-13). IR (film) *v*_max_ 2934, 1762 (C=O), 1576, 1477, 1278, 1223, 1076, 1004, and 753 cm^−1^. HRMS (ESI): *m*/*z* calcd. for C_43_H_54_N_5_O_7_ [M + H]^+^: 752.4023, found 752.4015.

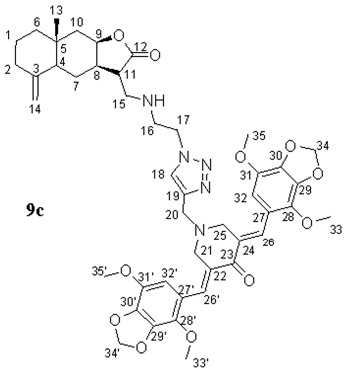

*(3E*,*5E)-3*,*5-bis((4*,*7-dimethoxybenzo[d][1,3]dioxol-5-yl)methylene)-1-((1-(2-((((3S*,*3aR*,*8aR*,*9aR)-8a-methyl-5-methylene-2-oxododecahydronaphtho[2*,*3-b]furan-3-yl)methyl)amino)ethyl)-1H-1*,*2*,*3-triazol-4-yl)methyl)piperidin-4-one* (**9c**), Yellow oil (64%). ^1^H NMR (400 MHz, CDCl_3_) δ 7.96 (2H, s, H-26, H-26′), 7.50 (1H, s, H-18), 6.38 (2H, s, H-32, H-32′), 6.03 (4H, s, H-34, H-34′), 4.76 (1H, s, H-14a), 4.48–4.38 (4H, m, H-9, H-14b, H_2_-17), 3.94 (6H, s, H_3_-33, H_3_-33′), 3.90 (6H, s, H_3_-35, H_3_-35′), 3.83 (2H, s H_2_-20), 3.80 (4H, s, H_2_-21, H_2_-25), 3.15-3.12 (1H, m, H-8), 3.02-3.00 (2H, m, H_2_-16), 2.84–2.82 and 2.75–2.70 (2H, both m, H_2_-15), 2.45–2.43 (1H, m, H-2a), 2.33–2.30 (1H, m, H-11), 2.15 (1H, d, *J* = 15.2 Hz, H-10a), 2.01–1.93 (1H, m, H-2b), 1.75 (1H, d, *J* = 12.0 Hz, H-6a), 1.60–1.45 (5H, m, H-1, H-4, H-7a, H-10b), 1.30–1.15 (2H, m, H-6b, H-7b), and 0.79 (3H, s, H_3_-13). ^13^C NMR (100 MHz, CDCl_3_) δ 186.65 (C-23), 177.86 (C-12), 149.08 (C-3), 143.79 (C-19), 138.62 (C-30, C-30′), 138.47 (C-29, C-29′), 138.05 (C-31, C-31′), 137.79 (C-28, C-28′), 132.15 (C-26, C-26′), 131.97 (C-22, C-24), 123.45 (C-18), 121.28 (C-27, C-27′), 109.29 (C-32, C-32′), 106.32 (C-14), 101.90 (C-34, C-34′), 78.26 (C-9), 60.33 (C-33, C-33′), 56.94 (C-35, C-35′), 54.41 (C-21, C-25), 51.65 (C-20), 49.75 (C-17), 49.01 (C-15), 47.06 (C-11), 46.24 (C-4), 44.80 (C-16), 42.02 (C-6), 41.21 (C-10), 38.86 (C-8), 36.55 (C-2), 34.61 (C-5), 22.51 (C-7), 20.91 (C-1), and 17.64 (C-13). IR (film) *v*_max_ 2931, 1762 (C=O), 1599, 1493, 1456, 1242, 1143, 1067, 963, and 755 cm^−1^. HRMS (ESI): *m*/*z* calcd. for C_45_H_54_N_5_O_11_ [M + H]^+^: 840.3820, found 840.3807.

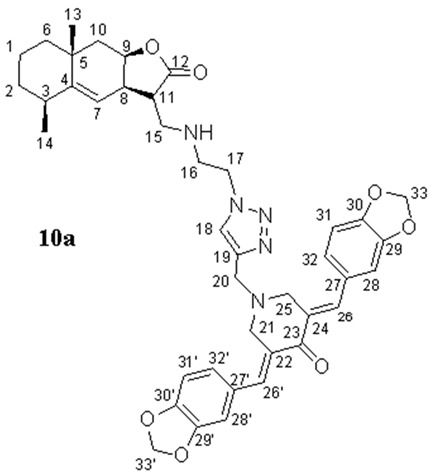

*(3E*,*5E)-3*,*5-bis(benzo[d][1,3]dioxol-5-ylmethylene)-1-((1-(2-((((3S*,*3aR*,*5S*,*8aR*,*9aR)-5*,*8a-dimethyl-2-oxo-2*,*3*,*3a*,*5*,*6*,*7*,*8*,*8a*,*9*,*9a-decahydronaphtho[2*,*3-b]furan-3-yl)methyl)amino)ethyl)-1H-1*,*2*,*3-triazol-4-yl)methyl)piperidin-4-one* (**10a**), Yellow oil (62%). ^1^H NMR (400 MHz, CDCl_3_) δ 7.69 (2H, s, H-26, H-26′), 7.55 (1H, s, H-18), 6.93–6.85 (6H, m, H-28, H-28′, H-31, H-31′, H-32, H-32′), 6.01 (4H, s, H-33, H-33′), 5.05 (1H, br.s, H-7), 4.72 (1H, br.s, H-9), 4.43–4.39 (2H, m, H_2_-17), 3.91 (2H, s, H_2_-20), 3.87 (4H, s, H_2_-21, H_2_-25), 3.13–2.88 (5H, m, H_2_-16, H_2_-15, H-8), 2.76–2.70 (1H, m, H-11), 2.45–2.44 (1H, m, H-3), 2.09 (1H, dd, *J* = 14.8 Hz, *J* = 2.8 Hz, H-10a), 1.81–1.75 (1H, m, H-2a), 1.60–1.43 (6H, m, H-1, H-2b, H-6, H-10b), 1.21 (3H, s, H_3_-13), and 1.10 (3H, d, *J* = 7.4 Hz, H_3_-14). ^13^C NMR (100 MHz, CDCl_3_) δ 186.91 (C-23), 177.66 (C-12), 151.16 (C-4), 148.33 (C-30, C-30′), 147.78 (C-29, C-29′), 144.04 (C-19), 136.05 (C-26, C-26′), 131.43 (C-27, C-27′), 129.17 (C-22, C-24), 125.72 (C-32, C-32′), 123.25 (C-18), 114.58 (C-7), 109.91 (C-31, C-31′), 108.47 (C-28, C-28′), 101.36 (C-33, C-33′), 77.29 (C-9), 54.48 (C-21, C-25), 52.40 (C-20), 49.85 (C-17), 48.98 (C-15), 46.04 (C-16), 45.43 (C-11), 42.48 (C-10), 42.00 (C-6), 38.30 (C-3), 37.46 (C-8), 32.86 (C-5), 32.64 (C-2), 28.49 (C-13), 22.80 (C-14), and 16.65 (C-1). IR (film) *v*_max_ 2927, 1758 (C=O), 1596, 1503, 1489, 1446, 1234, 1038, 929, and 734 cm^−1^. HRMS (ESI): *m*/*z* calcd. for C_41_H_46_N_5_O_7_ [M + H]^+^: 720.3397, found 720.3380.

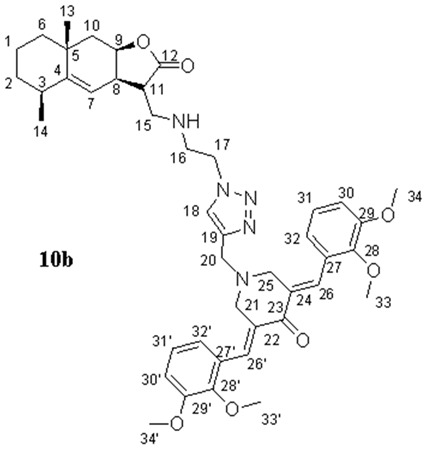

*(3E*,*5E)-3*,*5-bis(2*,*3-dimethoxybenzylidene)-1-((1-(2-((((3S*,*3aR*,*5S*,*8aR*,*9aR)-5*,*8a-dimethyl-2-oxo-2*,*3*,*3a*,*5*,*6*,*7*,*8*,*8a*,*9*,*9a-decahydronaphtho[2*,*3-b]furan-3-yl)methyl)amino)ethyl)-1H-1*,*2*,*3-triazol-4-yl)methyl)piperidin-4-one* (**10b**), Yellow oil (59%). ^1^H NMR (400 MHz, CDCl_3_) δ 8.03 (2H, s, H-26, H-26′), 7.44 (1H, s, H-18), 7.06 (2H, t, *J* = 7.9 Hz, H-31, H-31′), 6.95 (2H, d, *J* = 7.9 Hz, H-30, H-30′), 6.80 (2H, d, *J* = 7.6 Hz, H-32, H-32′), 5.06 (1H, s, H-7), 4.73 (1H, s, H-9), 4.35–4.33 (2H, m, H_2_-17), 3.89–3.80 (18H, m, H_3_-33, H_3_-33′, H_3_-34, H_3_-34′, H_2_-20, H_2_-21, H_2_-25), 3.07 (2H, br.s, H_2_-16), 3.02–2.91 (3H, m, H_2_-15, H-8), 2.74 (1H, br.s, H-11), 2.48–2.43 (1H, m, H-3), 2.09 (1H, dd, *J* = 14.7 Hz, *J* = 2.9 Hz, H-10a), 1.86–1.76 (1H, m, H-2a), 1.60–1.48 (6H, m, H-1, H-2b, H-6, H-10b), 1.21 (3H, s, H_3_-13), and 1.10 (3H, d, *J* = 7.4 Hz, H_3_-14). ^13^C NMR (100 MHz, CDCl_3_) δ 187.06 (C-23), 177.66 (C-12), 152.70 (C-28, C-28′), 151.09 (C-4), 148.22 (C-29, C-29′), 144.09 (C-19), 133.67 (C-27, C-27′), 132.43 (C-26, C-26′), 129.33 (C-22, C-24), 123.70 (C-18), 123.62 (C-32, C-32′), 121.83 (C-31, C-31′), 114.63 (C-7), 113.13 (C-30, C-30′), 77.31 (C-9), 61.10 (C-33, C-33′), 55.69 (C-34, C-34′), 54.31 (C-21, C-25), 51.58 (C-20), 49.70 (C-17), 49.01 (C-15), 45.98 (C-16), 45.49 (C-11), 42.50 (C-10), 42.01 (C-6), 38.29 (C-3), 37.44 (C-8), 32.86 (C-5), 32.64 (C-2), 28.50 (C-13), 22.81 (C-14), and 16.65 (C-1). IR (film) *v*_max_ 2930, 1759 (C=O), 1576, 1477, 1263, 1224, 1076, 1006, and 753 cm^−1^. HRMS (ESI): *m*/*z* calcd. for C_43_H_54_N_5_O_7_ [M + H]^+^: 752.4023, found 752.4018.

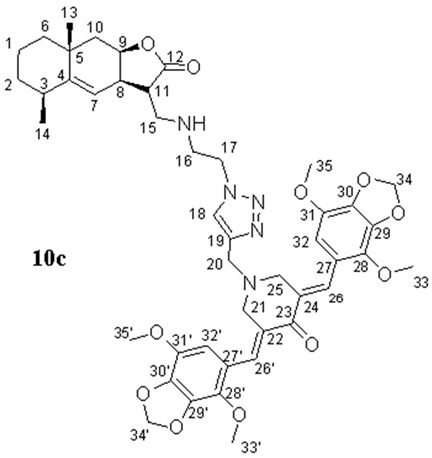

*(3E*,*5E)-3*,*5-bis((4*,*7-dimethoxybenzo[d][1,3]dioxol-5-yl)methylene)-1-((1-(2-((((3S*,*3aR*,*5S*,*8aR*,*9aR)-5*,*8a-dimethyl-2-oxo-2*,*3*,*3a*,*5*,*6*,*7*,*8*,*8a*,*9*,*9a-decahydronaphtho[2*,*3-b]furan-3-yl)methyl)amino)ethyl)-1H-1*,*2*,*3-triazol-4-yl)methyl)piperidin-4-one* (**10c**), Yellow oil (65%). ^1^H NMR (400 MHz, CDCl_3_) δ 7.93 (2H, s, H-26, H-26′), 7.50 (1H, s, H-18), 6.36 (2H, s, H-32, H-32′), 6.00 (4H, s, H-34, H-34′), 5.04 (1H, br.s, H-7), 4.72 (1H, br.s, H-9), 4.40–4.31 (2H, m, H_2_-17), 3.91–3.77 (18H, m, H_3_-33, H_3_-33′, H_3_-35, H_3_-35′, H_2_-20, H_2_-21, H_2_-25), 3.10–3.00 (3H, m, H_2_-16, H-8), 2.98–2.90 (2H, m, H_2_-15), 2.73–2.71 (1H, m, H-11), 2.43–2.41 (1H, m, H-3), 2.09–1.99 (1H, m, H-10a), 1.84–1.75 (1H, m, H-2a), 1.59–1.42 (6H, m, H-1, H-2b, H-6, H-10b), 1.19 (3H, s, H_3_-13), and 1.08 (3H, d, J = 7.4 Hz, H_3_-14). ^13^C NMR (100 MHz, CDCl_3_) δ 186.66 (C-23), 177.72 (C-12), 151.09 (C-4), 143.71 (C-19), 138.56 (C-30, C-30′), 138.42 (C-29, C-29′), 138.00 (C-28, C-28′), 132.05 (C-26, C-26′), 131.96 (C-22, C-24), 123.39 (C-18), 121.22 (C-27, C-27′), 114.58 (C-7), 109.24 (C-32, C-32′), 101.85 (C-34, C-34′), 101.36 (C-33, C-33′), 77.29 (C-9), 60.27 (C-33, C-33′), 56.87 (C-35, C-35′), 54.39 (C-21, C-25), 51.66 (C-20), 49.79 (C-17), 48.93 (C-15), 45.98 (C-16), 45.39 (C-11), 42.44 (C-10), 41.96 (C-6), 38.26 (C-3), 37.42 (C-8), 32.81 (C-5), 32.60 (C-2), 28.44 (C-13), 18.19 (C-14), and 16.61 (C-1). IR (film) v_max_ 2931, 1760 (C=O), 1600, 1495, 1456, 1245, 1143, 1067, and 736 cm^−1^. HRMS (ESI): *m*/*z* calcd. for C_45_H_54_N_5_O_11_ [M + H]^+^: 840.3820, found 840.3822.

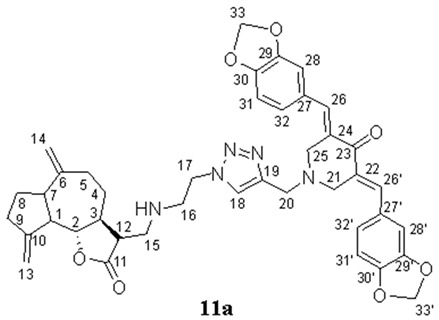

*(3E*,*5E)-3*,*5-bis(benzo[d][1,3]dioxol-5-ylmethylene)-1-((1-(2-((((3R*,*3aS)-6*,*9-dimethylene-2-oxododecahydroazuleno[4*,*5-b]furan-3-yl)methyl)amino)ethyl)-1H-1*,*2*,*3-triazol-4-yl)methyl)piperidin-4-one* (**11a**), Yellow oil (62%). ^1^H NMR (400 MHz, CDCl_3_) δ 7.72 (2H, s, H-26, H-26′), 7.57 (1H, s, H-18), 6.95–6.87 (6H, m, H-28, H-28′, H-31, H-31′, H-32, H-32′), 6.03 (4H, s, H-33, H-33′), 5.15 (1H, s, H-13a), 5.04 (1H, s, H-13b), 4.87 (1H, s, H-14a), 4.77 (1H, s, H-14b), 4.44–4.42 (2H, m, H_2_-17), 3.96–3.90 (7H, m, H-2, H_2_-20, H_2_-21, H_2_-25), 3.12–3.11 (2H, m, H_2_-16), 2.90–2.78 (4H, m, H_2_-15, H-12, H-7), 2.51–2.43 (3H, m, H-1, H-9), 2.37–2.35 (1H, m, H-5a,), 2.23–2.15 (1H, m, H-4a), 2.04–1.83 (4H, m, H-3, H-5b, H-8), and 1.34–1.24 (1H, m, H-4b). ^13^C NMR (100 MHz, CDCl_3_) δ 186.96 (C-23), 177.44 (C-11), 151.51 (C-10), 149.54 (C-6), 148.41 (C-30, C-30′), 147.83 (C-29, C-29′), 143.87 (C-19), 136.43 (C-26, C-26′), 131.10 (C-27, C-27′), 129.15 (C-22, C-24), 125.87 (C-32, C-32′), 123.47 (C-18), 111.88 (C-13), 109.97 (C-31, C-31′), 109.06 (C-14), 108.54 (C-28, C-28′), 101.49 (C-33, C-33′), 85.73 (C-2), 54.37 (C-21, C-25), 52.24 (C-20), 51.67 (C-12), 49.60 (C-17), 48.96 (C-15), 46.97 (C-1), 46.86 (C-7), 46.80 (C-3), 44.82 (C-16), 37.39 (C-9), 32.39 (C-5), 32.34 (C-4), and 30.03 (C-8). IR (film) *v*_max_ 2934, 1762 (C=O), 1576, 1477, 1263, 1223, 1076, 1004, and 753 cm^−1^. HRMS (ESI): *m*/*z* calcd. for C_41_H_44_N_5_O_7_ [M + H]^+^: 718.3240, found 718.3227.

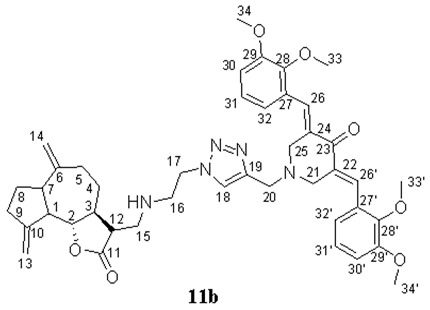

*(3E*,*5E)-3*,*5-bis(2*,*3-dimethoxybenzylidene)-1-((1-(2-((((3R*,*3aS)-6*,*9-dimethylene-2-oxododecahydroazuleno[4*,*5-b]furan-3-yl)methyl)amino)ethyl)-1H-1*,*2*,*3-triazol-4-yl)methyl)piperidin-4-one* (**11b**), Yellow oil (59%). ^1^H NMR (400 MHz, CDCl_3_) δ 8.06 (2H, s, H-26, H-26′), 7.46 (1H, s, H-18), 7.09–7.07 (2H, m, H-31, H-31′), 6.96 (2H, d, *J* = 7.6 Hz, H-30, H-30′), 6.82–6.81 (2H, m, H-32, H-32′), 5.17 (1H, s, H-13a), 5.05 (1H, s, H-13b), 4.87 (1H, s, H-14a), 4.77 (1H, s, H-14b), 4.34 (2H, br.s, H_2_-17), 3.91–3.85 (19H, m, H_3_-33, H_3_-33′, H_3_-34, H_3_-34′, H-2, H_2_-20, H_2_-21, H_2_-25), 3.03 (2H, br.s, H_2_-16), 2.87–2.77 (4H, m, H_2_-15, H-12, H-7), 2.52–2.44 (3H, m, H-1, H-9), 2.35 (1H, br.s, H-5a), 2.19 (1H, br.s, H-4a), 2.02–1.86 (4H, m, H-3, H-5b, H-8), and 1.29–1.27 (1H, m, H-4b). ^13^C NMR (100 MHz, CDCl_3_) δ 187.17 (C-23), 177.66 (C-11), 152.83 (C-28, C-28′), 151.76 (C-10), 149.76 (C-6), 148.35 (C-29, C-29′), 144.15 (C-19), 133.78 (C-27, C-27′), 132.59 (C-26, C-26′), 129.45 (C-22, C-24), 123.81 (C-32, C-32′), 123.33 (C-18), 121.99 (C-31, C-31′), 113.28 (C-30, C-30′), 111.87 (C-13), 109.05 (C-14), 85.77 (C-2), 61.27 (C-33, C-33′), 55.84 (C-34, C-34′), 54.45 (C-21, C-25), 51.81 (C-12), 51.67 (C-20), 49.93 (C-17), 49.17 (C-15), 47.39 (C-1), 46.94 (C-7), 46.81 (C-16), 44.85 (C-3), 37.55 (C-9), 32.54 (C-5), 32.50 (C-4), and 30.14 (C-8). IR (film) *v*_max_ 2933, 1764 (C=O), 1575, 1476, 1455, 1263, 1224, 1075, 1004, and 753 cm^−1^. HRMS (ESI): *m*/*z* calcd. for C_43_H_52_N_5_O_7_ [M + H]^+^: 750.3866, found 750.3855.

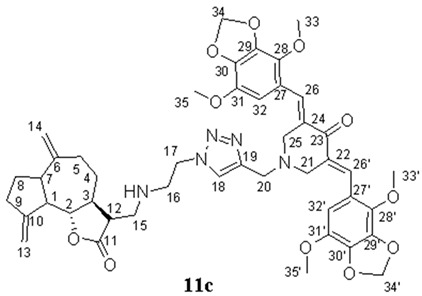

*(3E*,*5E)-3*,*5-bis((4*,*7-dimethoxybenzo[d][1,3]dioxol-5-yl)methylene)-1-((1-(2-((((3R*,*3aS)-6*,*9-dimethylene-2-oxododecahydroazuleno[4*,*5-b]furan-3-yl)methyl)amino)ethyl)-1H-1*,*2*,*3-triazol-4-yl)methyl)piperidin-4-one* (**11c**), Yellow oil (57%). ^1^H NMR (400 MHz, CDCl_3_) δ 7.92 (2H, s, H-26, H-26′), 7.46 (1H, s, H-18), 6.35 (2H, s, H-32, H-32′), 5.99 (4H, s, H-34, H-34′), 5.12 (1H, s, H-13a), 4.99 (1H, s, H-13b), 4.83 (1H, s, H-14a), 4.73 (1H, s, H-14b), 4.32 (2H, br.s, H_2_-17), 3.90–3.77 (19H, m, H-33, H-33′, H-35, H-35′, H-2, H_2_-20, H_2_-21, H_2_-25), 2.99–2.82 (6H, m, H_2_-16, H_2_-15, H-12, H-7), 2.48–2.40 (3H, m, H-1, H-9), 2.27–1.80 (6H, m, H-5a, H-4a, H-3, H-5b, H-8), and 1.27–1.22 (1H, m, H-4b). ^13^C NMR (100 MHz, CDCl_3_) δ 186.56 (C-23), 177.42 (C-11), 151.51 (C-10), 149.54 (C-6), 143.67 (C-19), 138.49 (C-30, C-30′), 138.35 (C-29, C-29′), 137.91 (C-31, C-31′), 137.69 (C-28, C-28′), 131.96 (C-22, C-24), 131.90 (C-26, C-26′), 123.26 (C-18), 121.18 (C-27, C-27′), 111.63 (C-13), 109.14 (C-32, C-32′), 108.88 (C-14), 101.82 (C-34, C-34′), 85.52 (C-2), 60.25 (C-33, C-33′), 56.80 (C-35, C-35′), 54.34 (C-21, C-25), 51.60 (C-20), 51.56 (C-12), 49.78 (C-17), 49.00 (C-15), 47.15 (C-1), 47.01(C-16), 46.71 (C-7), 44.63 (C-3), 37.33 (C-9), 32.29 (C-5), 32.24 (C-4), and 29.90 (C-8). IR (film) *v*_max_ 2937, 1763 (C=O), 1599, 1495, 1456, 1193, 1066, and 755 cm^−1^. HRMS (ESI): *m*/*z* calcd. for C_45_H_52_N_5_O_11_ [M + H]^+^: 838.3663, found 838.3661.

### 3.6. Cell Culture

Cells of tumor origin—epithelial cells of breast adenocarcinoma MCF-7, neuroblastomes SH-SY5Y and IMR-32, human cervical adenocarcinoma HeLa, and conditionally normal fibroblast culture WI-38 obtained from fetal lung tissue—were cultivated in a wet chamber using DMEM supplemented with 10% fetal calf serum and penicillin as an antibiotic at 37 °C and 5% CO_2_. After 75% of confluence was reached, the cells were trypsinized using a physiological buffer salt solution containing 0.25% trypsin. Next, the cells were transferred to 96-well culture plates at a density of 10,000/well and incubated for 24 h to attach to the plate surface.

### 3.7. Assessment of Cells Vitality

Cell vitality was assessed by the MTT test. The cells were treated with solutions of the studied compounds at different concentrations (ranging from 0.1 to 100 µM) for 24 h. After incubation, 3-(4,5-dimethylthiazol-2-yl)-2,5-diphenyl-2*H*-tetrazolium bromide (MTT) was added to all wells in an amount equal to 10% of the nutrition medium volume and incubated for 2 h at 37 °C. Next, the nutrition medium was carefully abstracted, and the resultant formazane crystals indicating cell vitality were dissolved by the addition of dimethyl sulfoxide. Finally, the optical density was measured using a Cytation^TM^3 plate analyzer (BioTek Instruments Inc., Winooski, VT, USA) at a wavelength of 555 nm.

### 3.8. Measuring Glycolytic Function in Cells

Glycolytic function in HeLa cells of tumor origin (human cervical adenocarcinoma) was assessed by glycolysis stress test using a Seahorse XFe96 (Agilent Technologies, Santa Clara, CA, USA) cell metabolism analyzer according to the protocol of Agilent Technologies (Santa Clara, CA, USA).

Briefly, HeLa cells that amounted to 30,000/well were seeded in a 96-well plate and cultured in Dulbecco’s Modified Eagle’s Medium supplemented with 10% fetal calf serum and penicillin at 37 °C in a humidified atmosphere with 5% CO_2_ for 24 h. After incubation, the cells were washed twice with Agilent Seahorse XF assay media containing DMEM and incubated for 1 h at 37 °C. Next, the plate with cells was placed in the cell metabolism analyzer and the external cell acidification rate of medium (ECAR) was measured during prescribed time intervals at the initial level and after automatic sequential injections of the following studied compounds/solvent (port A): 10 mM glucose (port B), 1 µM oligomycin (port C), and 25 mM 2-deoxyglucose (port D). The ECAR values for each group were a combination of three independent assays.

### 3.9. Molecular Docking

Structures of hexokinase 2,6-phosphofructo-2-kinase, and human pyruvate kinase M2 were obtained from a protein data bank (PDB) (www.rcsb.org (on 1 February 2024)), with PDB ID 2NZT [84,85], 1K6M [86], and 6V74 [87], respectively. The structure of target proteins was prepared for molecular docking procedure using the AutoDockVinapackage software (version 1.1.2, Molecular Graphics Laboratory, The Scripps Research Institute, LaJolla, CA, USA) and UCSF Chimera 1.17.3 (Resource for Biocomputing, Visualization, and Informatics from the University of California, San Francisco, CA, USA) by removing various ligands, non-key waters, and other non-key small molecules, as well as by adding H atoms and charge sand missing side chains.

The 3D structures of the ligand molecules were obtained by the ChemDrawUltra 12.0 software. Structure minimization for all ligands was performed using the UCSF Chimera software.

To validate the reference, molecules (2-Deoxy-glucose-6-phosphate for hexokinase 2 and L-phenylalanine for pyruvate kinase M2) were redocked in accordance with the parameters used in the specific experiment. The compounds were docked to the selected cavities of the enzymes with ten iterations for each specific docking. The docking procedure was considered to be successful when the obtained results had an RMSD parameter below 1.

Subsequent treatment and analysis of results and image production were performed using the Biovia Discovery Studio Viewer 2021 software (Biovia, SanDiego, CA, USA).

## 4. Conclusions

In conclusion, in this work, we have synthesized previously unknown hybrid molecular systems composed of 3,5-bis(arylidene)-4-piperidone and sesquiterpene lactones using click-chemistry methodology. We have shown that, as cytotoxic agents show selective action toward tumor cells, the conjugates of 3,5-bis(arylidene)piperidin-4-ones and sesquiterpene lactones display a modulating action on glucose metabolism, which is caused by their glycolysis-inhibiting ability. Using molecular in silico screening, the obtained compounds were identified as potential inhibitors of the allosteric glycolytic enzyme, pyruvate kinase M2 oncoprotein.

Our study is one of the first to demonstrate the mechanism of action of compounds based on the piperidone platform associated with the reprogramming of the metabolic state of tumor cells by the inhibition of PKM2. At the same time, this study expands the understanding of the antitumor potential of compounds with such a structure and provides the foundation for the further analysis of compounds containing piperidone fragments in their structure in terms of efficiency against the key enzymes of the glycolysis process.

Thus, the obtained results indicate that conjugates of 3,5-bis(arylidene)piperidin-4-ones and sesquiterpene lactones can be considered promising platforms for designing selective antitumor agents targeted against the glycolysis process.

## Figures and Tables

**Table 1:** Cytotoxicity profiles of the prepared compounds.

	IC_50_ of Cytotoxic Effect, µM				
	MCF-7	SH-SY5Y	HeLa	IMR-32	WI-38
**1**	27.51 ± 0.20	19.22 ± 0.1	13.41 ± 0.12	26.83 ± 0.27	74.03 ± 0.11
**2**	13.15 ± 0.13	15.31 ± 0.09	10.05 ± 0.03	30.26 ± 1.10	48.45 ± 0.14
**3**	10.56 ± 0.07	9.15 ± 0.11	7.24 ± 0.06	22.00 ± 0.98	38.55 ± 0.86
**7a**	23.93 ± 0.08	4.75 ± 0.03	9.21 ± 0.03	25.66 ± 0.63	5.26 ± 0.07
**7b**	6.73 ± 0.05	4.83 ± 0.02	43.99 ± 0.37	9.05 ± 0.09	2.63 ± 0.08
**7c**	6.95 ± 0.02	5.55 ± 0.05	28.09 ± 0.35	24.14 ± 0.08	0.66 ± 0.05
**9a**	8.90 ± 0.18	8.84 ± 0.01	57.29 ± 1.69	8.73 ± 0.02	39.47 ± 0.07
	SI = 4	SI = 4		SI = 5	
**9b**	9.44 ± 0.13	8.30 ± 0.01	8.77 ± 0.02	6.72 ± 0.07	28.08 ± 0.03
	SI = 3	SI = 3	SI = 3	SI = 4	
**9c**	24.88 ± 0.21	21.95 ± 0.23	19.81 ± 0.17	21.95 ± 0.21	35.65 ± 0.08
**10a**	8.60 ± 0.16	10.01 ± 0.09	9.97 ± 0.13	16.41 ± 0.07	18.48 ± 0.12
**10b**	8.17 ± 0.05	7.93 ± 0.05	22.68 ± 0.08	5.76 ± 0.03	29.53 ± 0.03
	SI = 4	SI = 4		SI = 5	
**10c**	18.60 ± 0.11	19.01 ± 0.08	21.31 ± 0.17	17.38 ± 0.07	28.44 ± 0.13
**11a**	8.91 ± 0.08	22.75 ± 0.34	21.06 ± 0.04	8.64 ± 0.07	32.75 ± 0.05
	SI = 4			SI = 4	
**11b**	8.07 ± 0.02	7.41 ± 0.09	6.58 ± 0.04	6.07 ± 0.06	40.36 ± 0.09
	SI = 5	SI = 5	SI = 6	SI = 7	
**11c**	11.46 ± 0.21	14.38 ± 0.11	22.73 ± 0.12	17.12 ± 0.17	22.38 ± 0.08
Arglabin	21.82 ± 0.34	15.06 ± 0.22	25.09 ± 0.45	30.23 ± 1.35	9.32 ± 0.01
Curcumin	15.24 ± 0.11	11.78 ± 0.34	12.76 ± 0.52	21.82 ± 0.91	26.12 ± 0.24

**Table 2:** Parameters of the glycolytic function of the HeLa cell line under the action of the studied compounds.

	Glycolysis	Glycolytic Capacity	Glycolytic Reserve
**Control**	49.18 ± 0.43	79.74 ± 3.14	30.56 ± 2.85
**1**	54.30 ± 2.46	77.18 ± 1.56	22.88 ± 0.90
**2**	53.51 ± 2.81	79.38 ± 2.50	25.87 ± 2.60
**3**	43.46 ± 6.58	77.18 ± 1.56	27.44 ± 5.27
**7a**	46.29 ± 2.53	62.68 ± 3.95 **	16.39 ± 1.75 *
**7b**	37.44 ± 4.10	54.29 ± 0.72 ****	16.85 ± 4.01 *
**7c**	35.03 ± 2.11	46.35 ± 2.99 ****	11.32 ± 3.63 ***
**9a**	41.37 ± 1.10	51.82 ± 3.82 ****	10.45 ± 0.52 ***
**9b**	35.79 ± 1.71	40.02 ± 3.88 ****	4.23 ± 2.32 ****
**9c**	36.07 ± 4.59	48.54 ± 2.55 ****	12.47 ± 4.31 **
**10a**	38.39 ± 4.74	56.49 ± 2.98 ****	14.08 ± 2.84 **
**10b**	37.11 ± 0.49	42.52 ± 1.35 ****	5.41 ± 0.87 ****
**10c**	35.46 ± 1.10	46.53 ± 2.37 ****	11.07 ± 1.57 ***
**11a**	34.32 ± 1.90	50.34 ± 4.93 ****	16.02 ± 4.49 *
**11b**	32.26 ± 3.66 *	34.77 ± 3.05 ****	2.51 ± 1.07 ****
**11c**	35.17 ± 1.39	44.02 ± 4.19 ****	8.86 ± 2.93 ***

The data are represented as Mean ± SEM, n = 3. *, **, ***, and ****—*p* < 0.05, *p* < 0.01, *p* < 0.001, and *p* < 0.0001 by 1-way ANOVA with Dunnett’s test.

**Table 3:** The binding affinities of the synthesized compounds against hexokinase 2, 6-phosphofructo-2-kinase, and pyruvate kinase M2.

	Binding Energy, kcal/mol		
	Hexokinase 2	6-Phosphofructo-2-Kinase	Pyruvate Kinase M2
**1**	−5.2	−4.1	−5.8
**2**	−4.8	−3.9	−6.0
**3**	−5.5	−4.2	−6.1
**7a**	−7.0	−7.3	−7.4
**7b**	−6.9	−7.0	−7.6
**7c**	−7.3	−7.8	−7.9
**9a**	−6.4	−6.2	−8.7
**9b**	−6.5	−6.5	−9.2
**9c**	−5.9	−6.1	−8.5
**10a**	−6.0	−6.4	−8.4
**10b**	−6.4	−6.2	−9.4
**10c**	−6.3	−5.9	−9.0
**11a**	−6.5	−6.5	−8.7
**11b**	−6.0	−6.3	−9.9
**11c**	−5.8	−6.0	−8.9
Reference compound *	−7.6	-	−8.0

* For each enzyme, positive control was accomplished individually (see detailed description in Section 3).

## Data Availability

All studied compounds can be found from the corresponding author V. K. Brel.

## Supplementary Materials

The following supporting information can be downloaded at: https://www.mdpi.com/article/10.3390/molecules29122765/s1, 1H, 13C NMR and HRMS spectra for all novel compounds **4**, **7c**, **8a**–**c**, **9a**–**c**, **10a**–**c**, **11a**–**c**.
