# Supplementary material for: Conjugates of 3,5-Bis(arylidene)-4-piperidone and Sesquiterpene Lactones Have an Antitumor Effect via Resetting the Metabolic Phenotype of Cancer Cells
Source: Molecules. 2024 Jun 11;29(12):2765. doi: 10.3390/molecules29122765 (PMC11207066; doi:10.3390/molecules29122765)
Supplement: Supplementary file 1 [file molecules-29-02765-s001.zip › molecules-3029308-supplementary.pdf]

Supplementary materials

Conjugates of 3,5-Bis(Arylidene)-4-Piperidone and Sesquiterpene Lactones Have an Antitumor Effect via the Resetting the Metabolic Phenotype of Cancer Cells

M.E. Neganova[1,2,†], Yu.R. Aleksandrova[1,2,†], E.V. Sharova[2], E.V. Smirnova[2], O.I. Artyushin[2], N.S. Nikolaeva[1], A.V. Semakov[1], I.A. Schagina[1], N. Akylbekov[3], R. Kurmanbayev[3], D. Orynbekov[3,*], V.K. Brel[2,*]

List of content:

1. NMR and HRMS spectra of compound **4** S2-S4

2. NMR and HRMS spectra of compound**7c** S5-S7

3. NMR and HRMS spectra of compounds **8a-c**S8-S16

4. NMR and HRMS spectra of compounds **9a-c**S17-S25

5. NMR and HRMS spectra of compounds **10a-c**S26-S34

6. NMR and HRMS spectra of compounds **11a-c**S35-S43

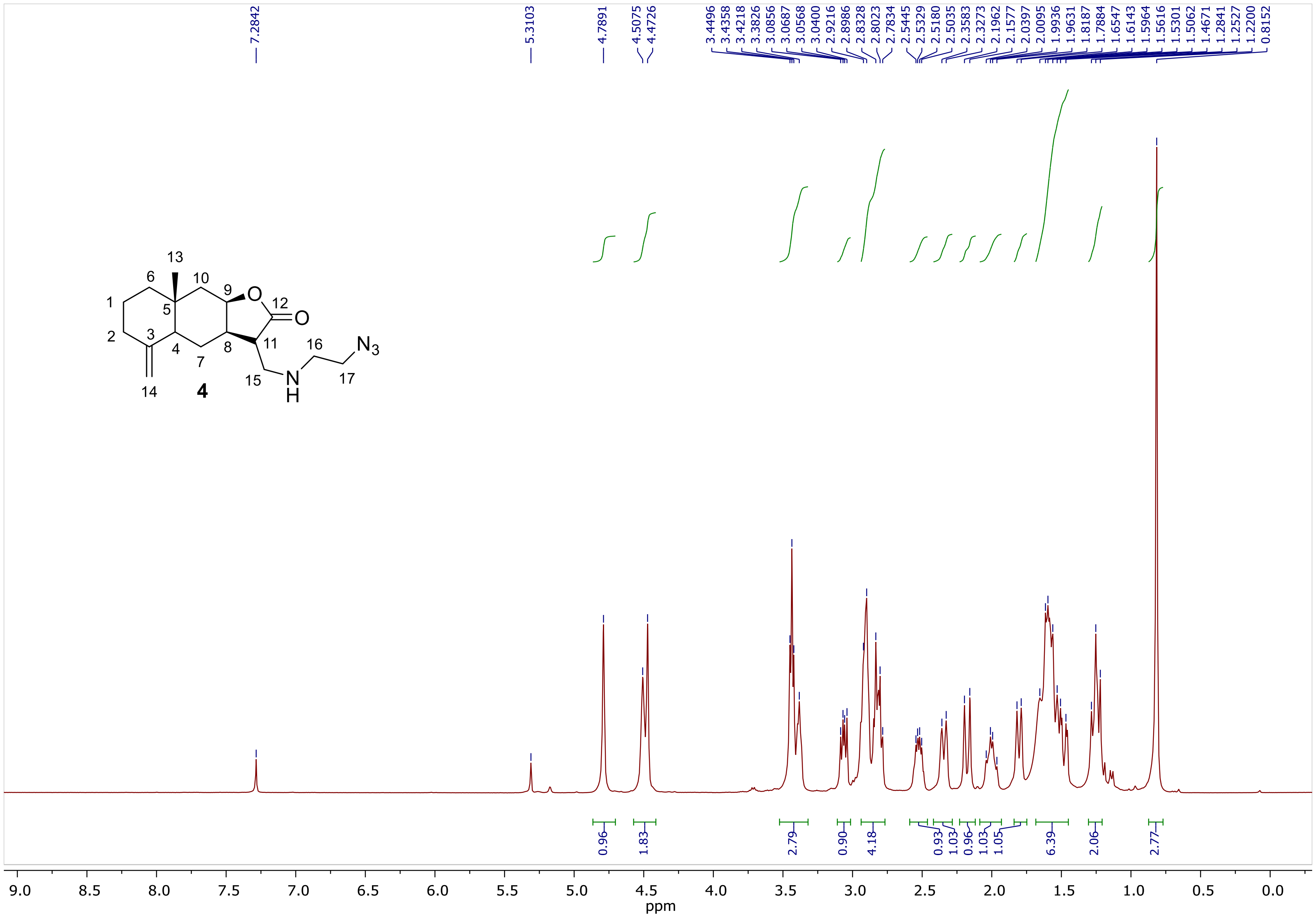

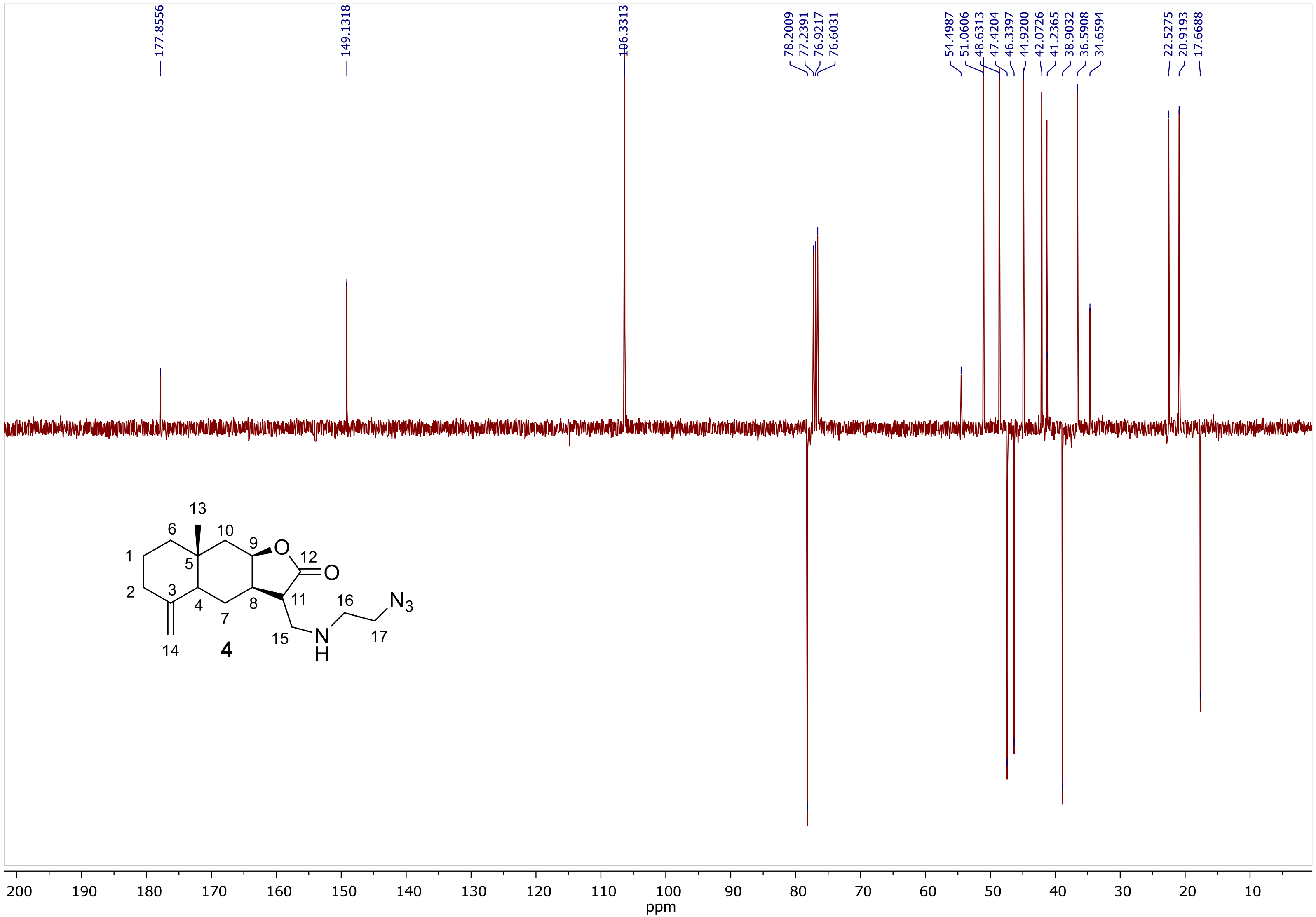

^{1}H and ^{13}C NMR spectra of compound **4** ($CDCl_3$)

4 HRMS (ESI): m/z calcd. for $C_{17}H_{27}N_4O_2$ $[M+H]^+$ 319.2129, found 319.2135.

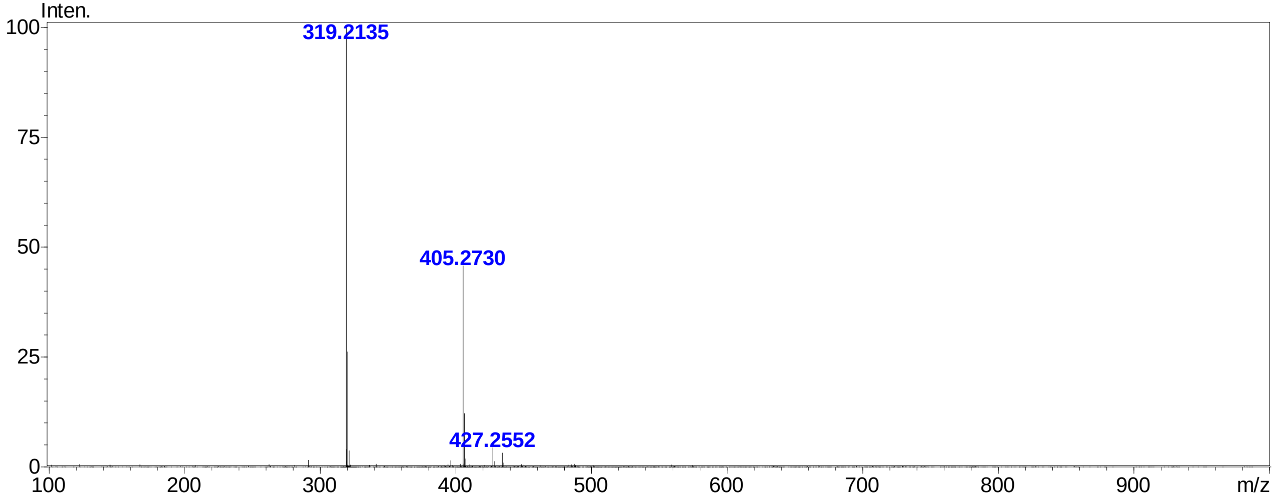

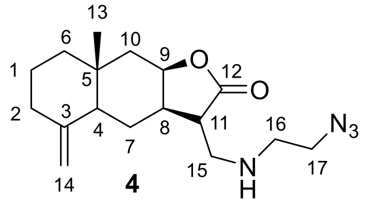

4

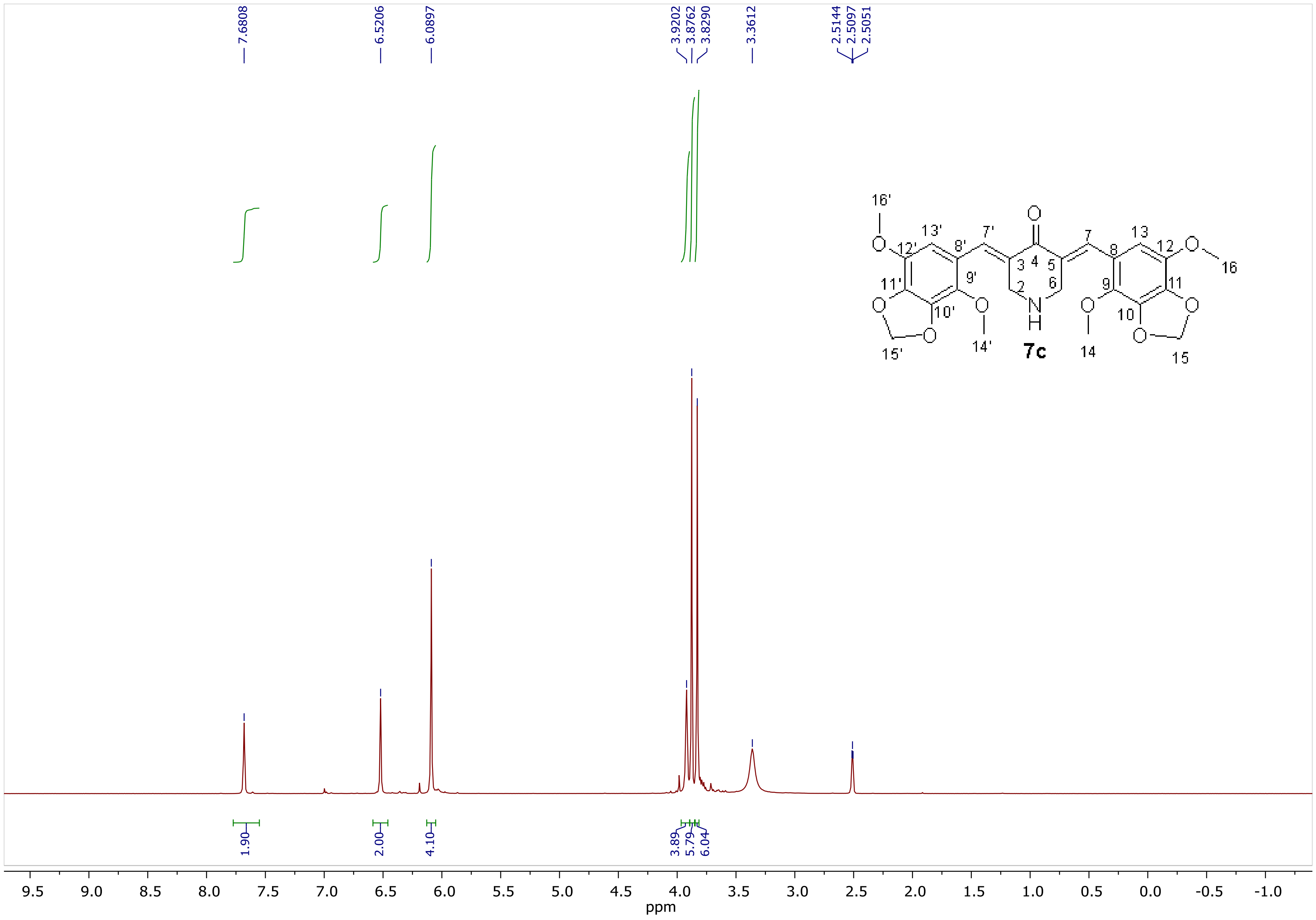
7.6808
6.5206
6.0897
3.9202
3.8762
3.8290
3.3612
2.5144
2.5097
2.5051
1.90
2.00
4.10
3.89
5.79
6.04
9.5
9.0
8.5
8.0
7.5
7.0
6.5
6.0
5.5
5.0
4.5
4.0
3.5
3.0
2.5
2.0
1.5
1.0
0.5
0.0
-0.5
-1.0
ppm
7c

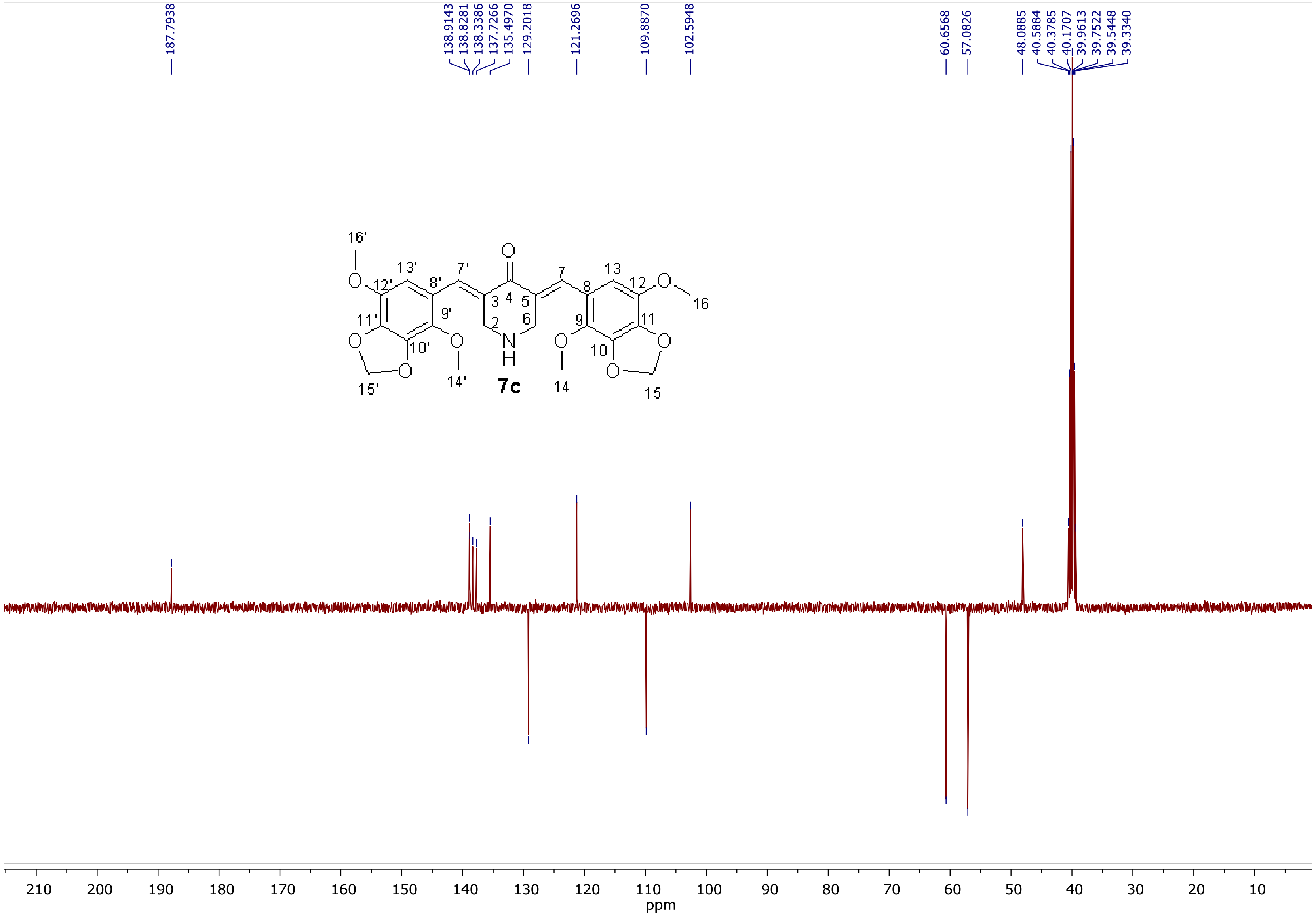

^{1}H and ^{13}C NMR spectra of compound **4** (DMSO-d$_6$)

7c HRMS (ESI): m/z calcd. for $C_{25}H_{26}NO_9$ [M+H]$^+$ 484.1602, found 484.1614

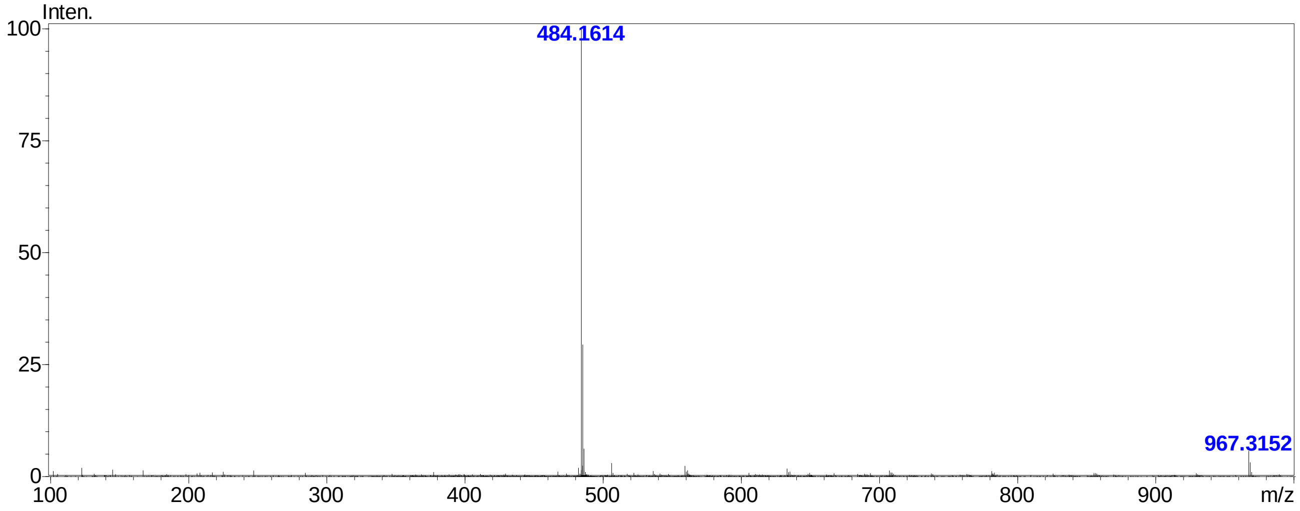

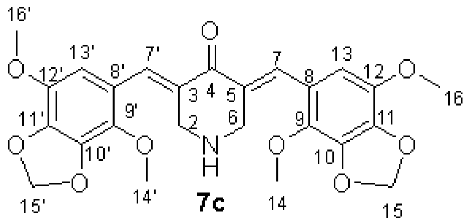

7c

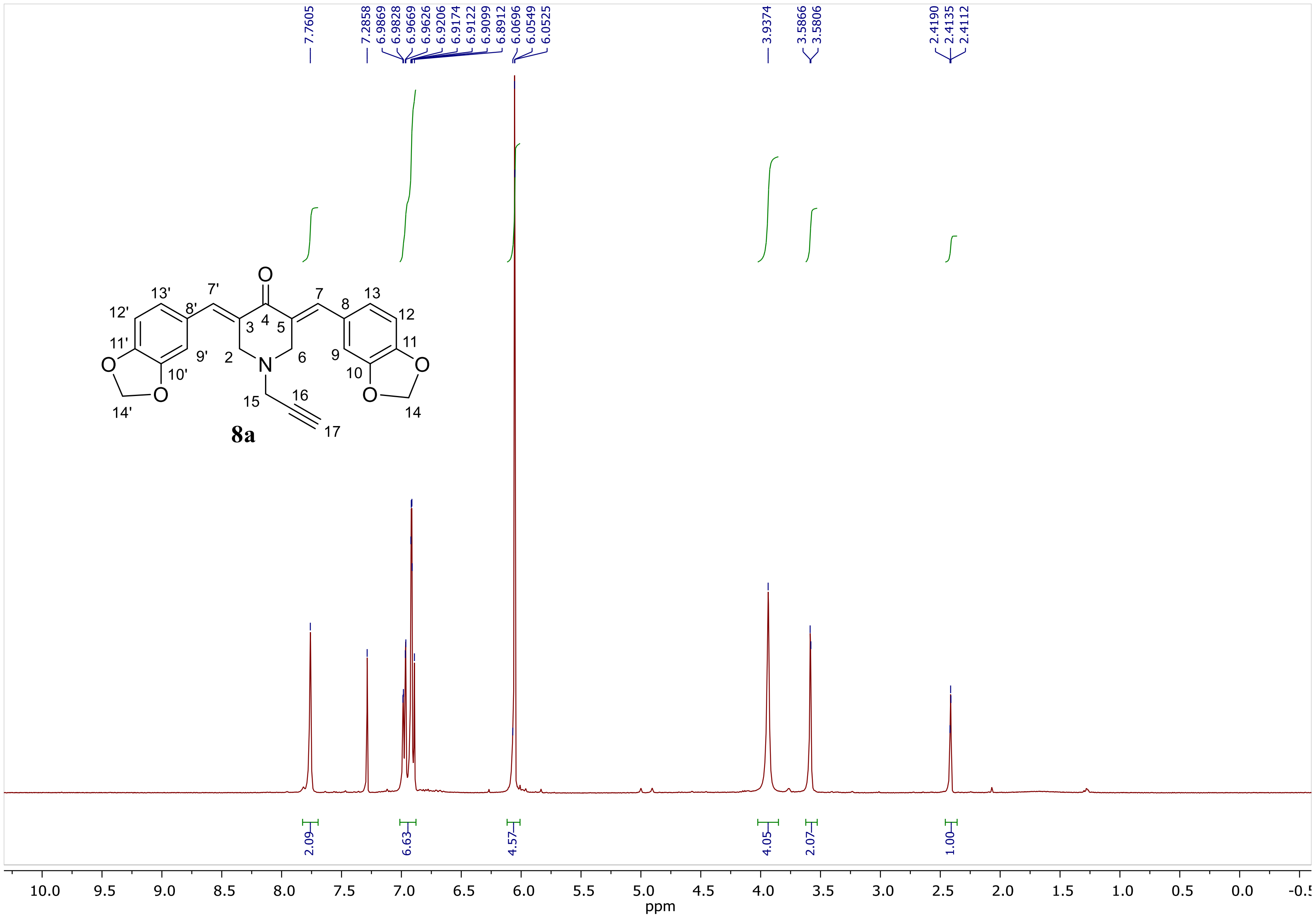

7.7605
7.2858
6.9869
6.9828
6.9669
6.9626
6.9206
6.9174
6.9122
6.9099
6.8912
6.0696
6.0549
6.0525
3.9374
3.5866
3.5806
2.4190
2.4135
2.4112
2.09
6.63
4.57
4.05
2.07
1.00
10.0 9.5 9.0 8.5 8.0 7.5 7.0 6.5 6.0 5.5 5.0 4.5 4.0 3.5 3.0 2.5 2.0 1.5 1.0 0.5 0.0
ppm
8a

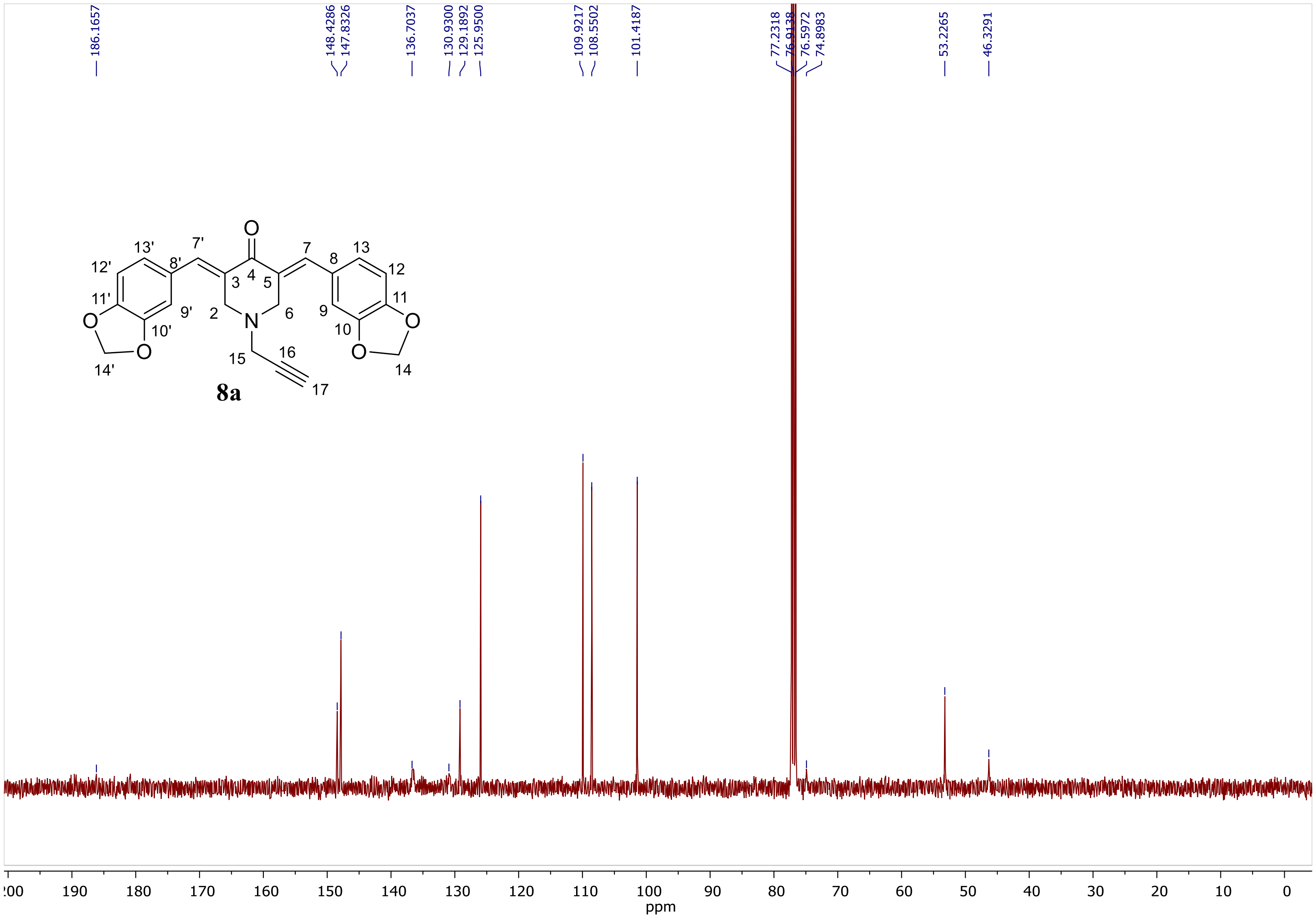

^{1}H and ^{13}C NMR spectra of compound **8a**($CDCl_3$)

8a HRMS (ESI): m/z calcd. for $C_{24}H_{20}NO_5$ $[M+H]^+$ 402.1336, found 402.1346.

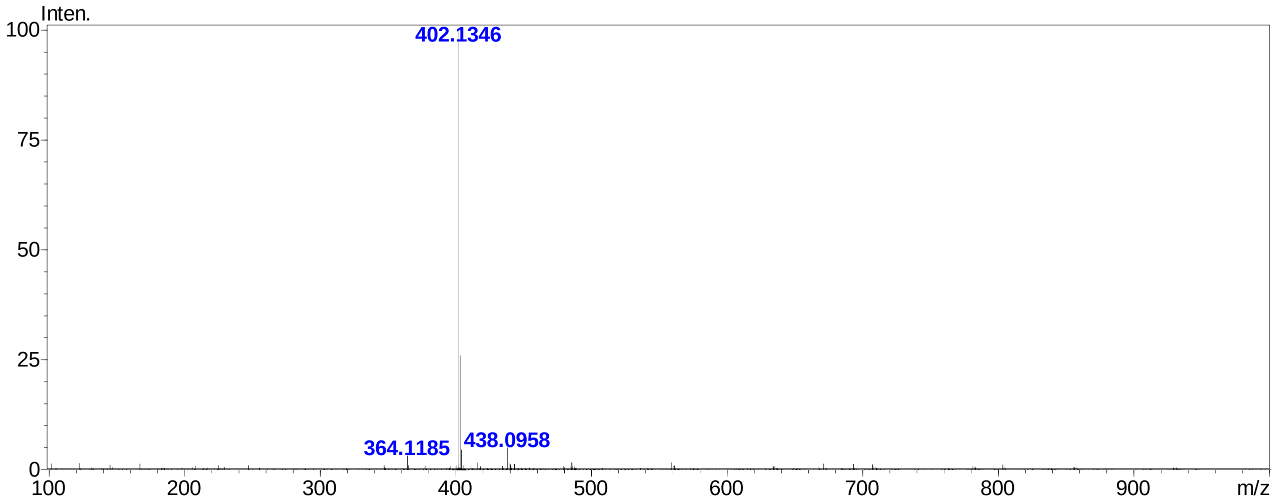

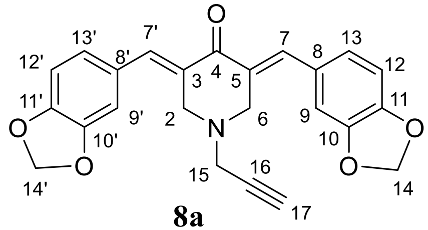

8a

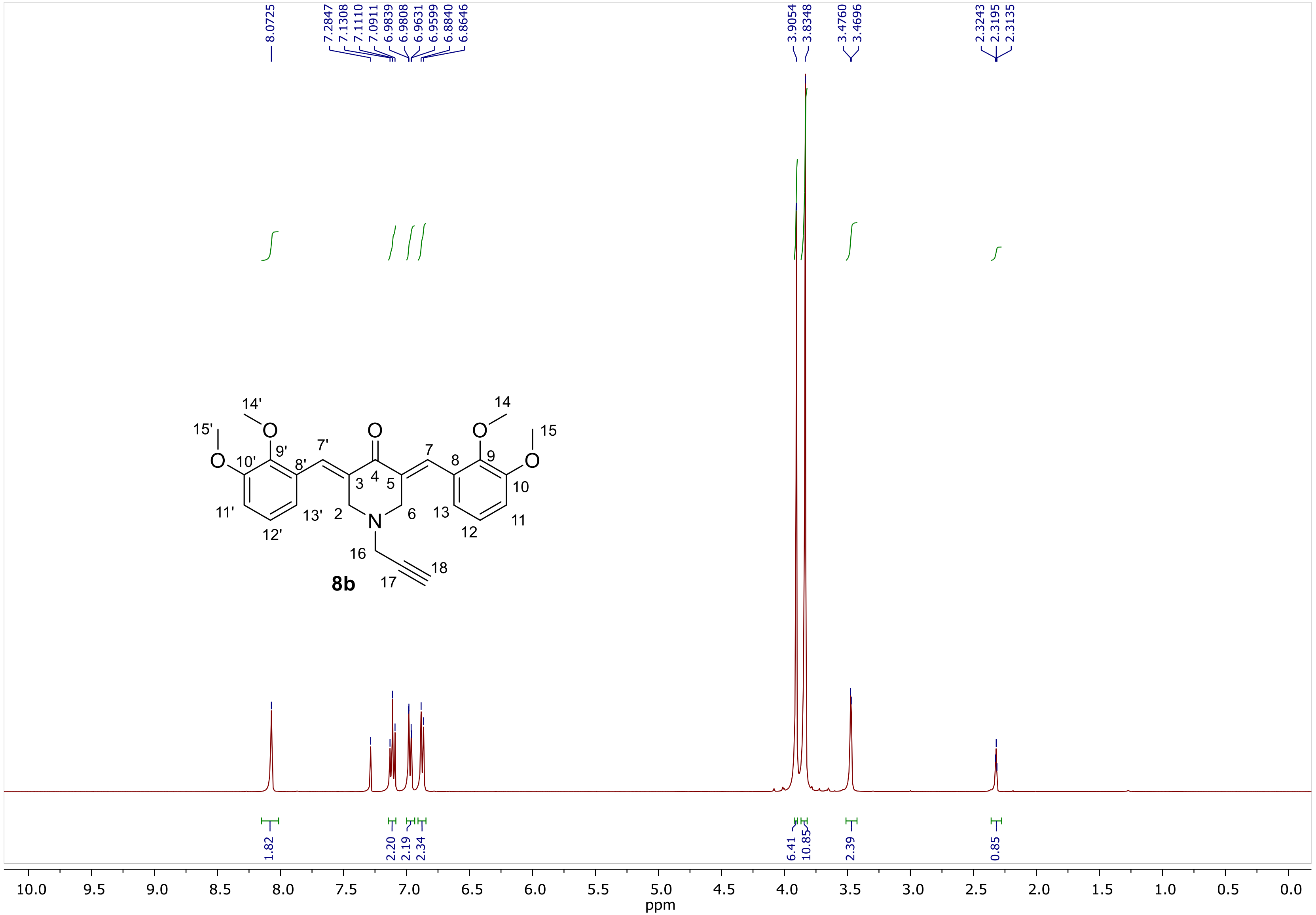
8.0725
7.2847
7.1308
7.1110
7.0911
6.9839
6.9808
6.9631
6.9599
6.8840
6.8646
3.9054
3.8348
3.4760
3.4696
2.3243
2.3195
2.3135
1.82
2.20
2.19
2.34
6.41
10.85
2.39
0.85
ppm
8b

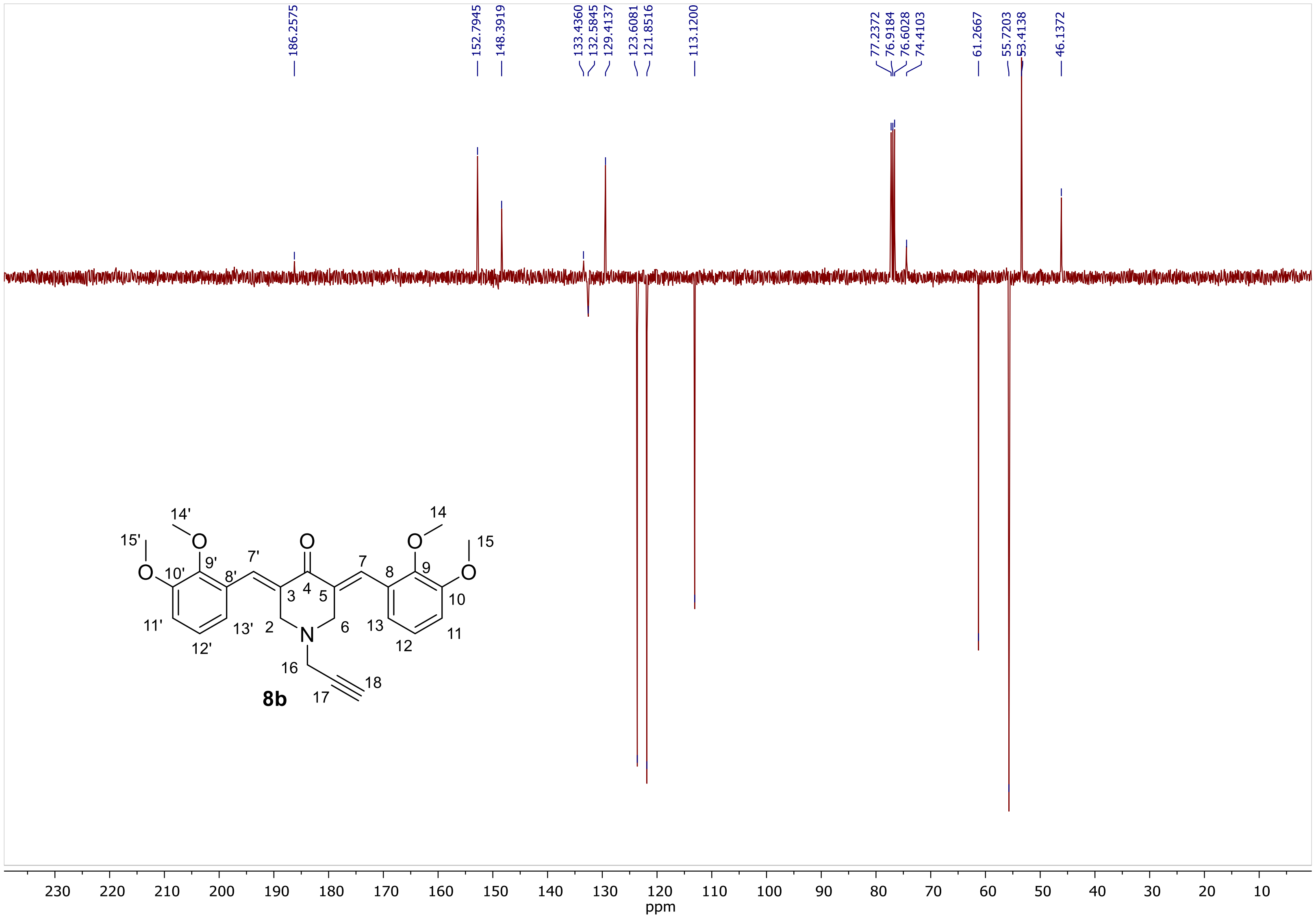

^{1}H and^{13}C NMR spectra of compound **8b**($CDCl_3$)

8b HRMS (ESI): m/z calcd. for $C_{26}H_{28}NO_5$ [M+H]$^+$ 434.1962, found 434.1971.

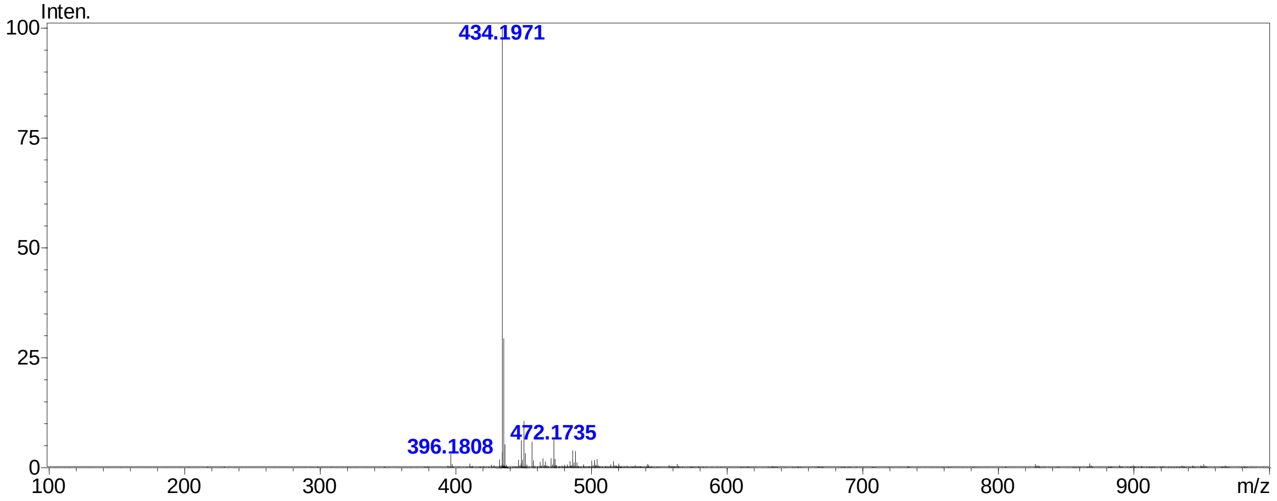

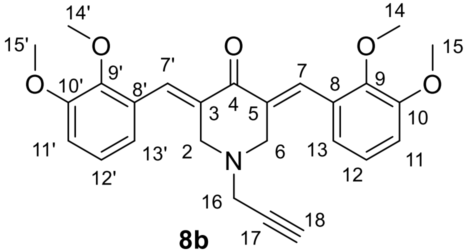

8b

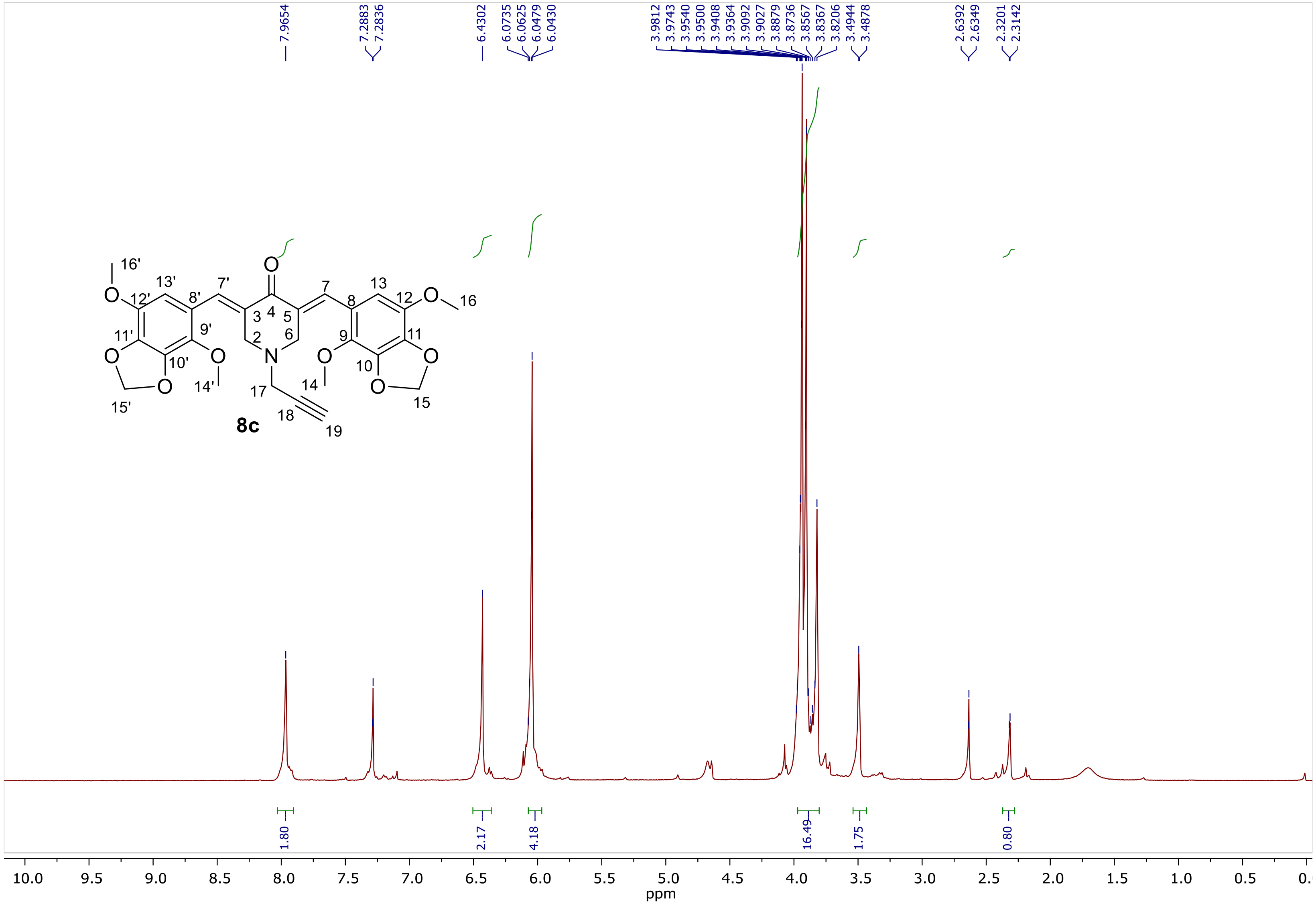
7.9654
7.2883
7.2836
6.4302
6.0735
6.0625
6.0479
6.0430
3.9812
3.9743
3.9540
3.9500
3.9408
3.9364
3.9092
3.9027
3.8879
3.8736
3.8567
3.8367
3.8206
3.4944
3.4878
2.6392
2.6349
2.3201
2.3142
8c
1.80
2.17
4.18
16.49
1.75
0.80
ppm

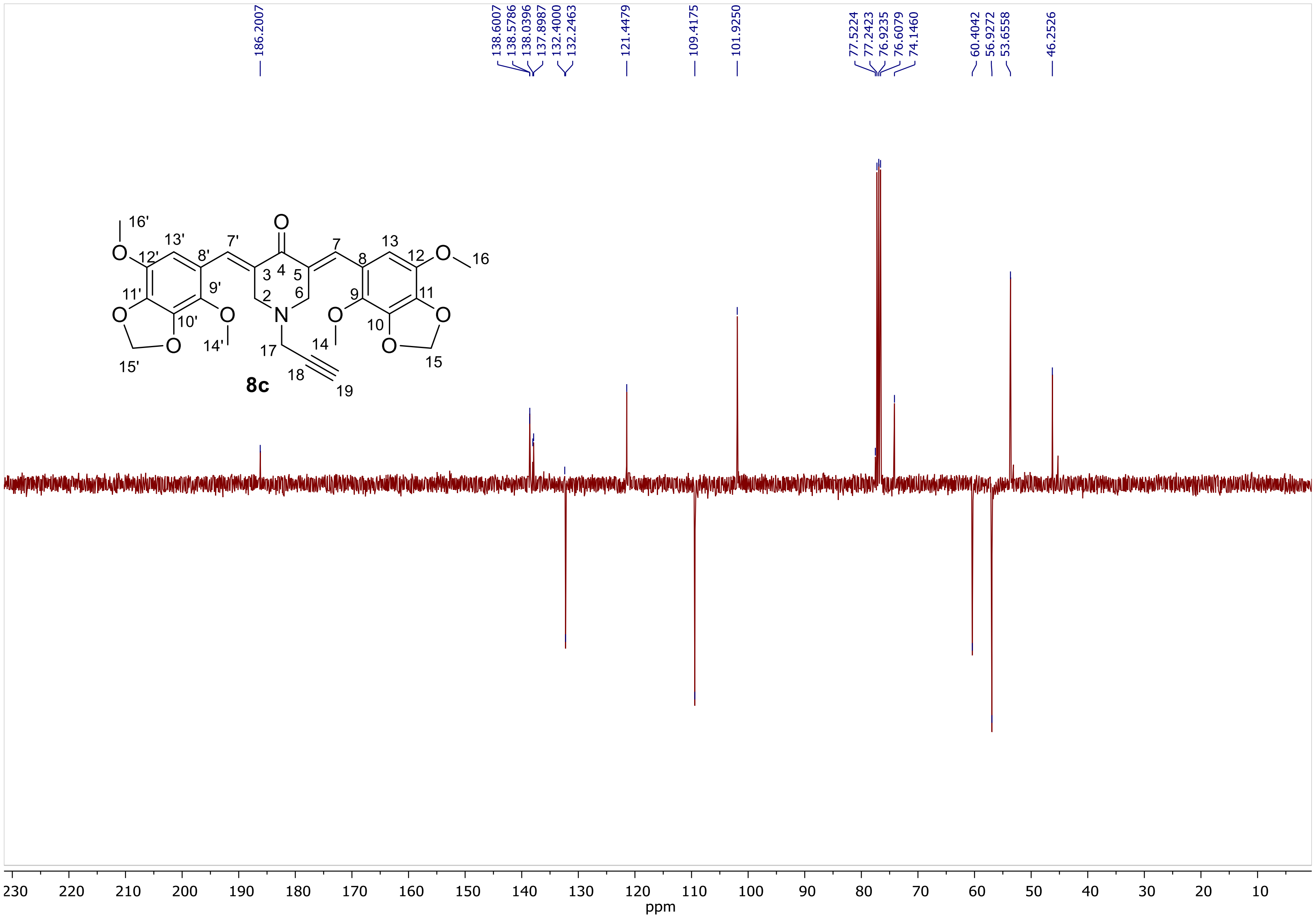

^{1}H and^{13}C NMR spectra of compound **8c**($CDCl_3$)

8c HRMS (ESI): m/z calcd. for $C_{28}H_{28}NO_9$ $[M+H]^+$ 522.1759, found 522.1765.

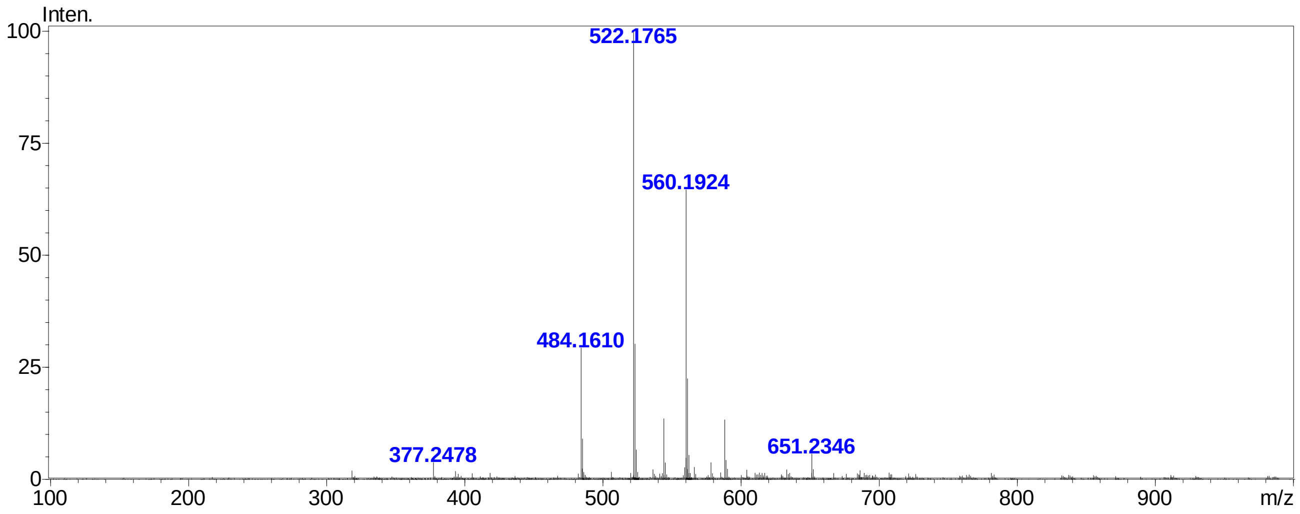

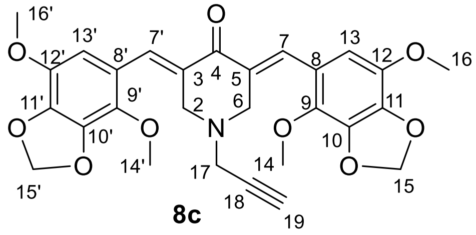

8c

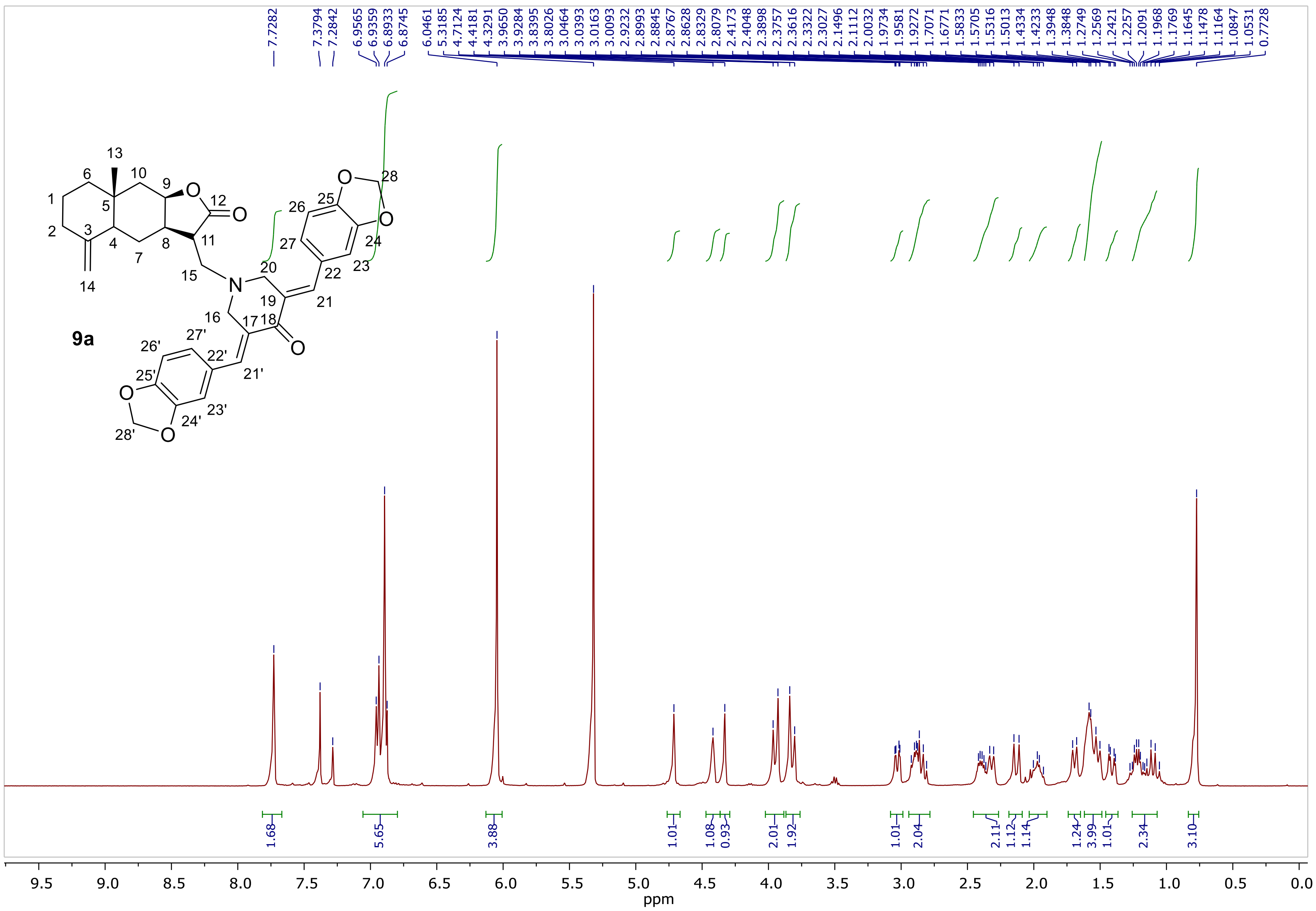

9a
ppm

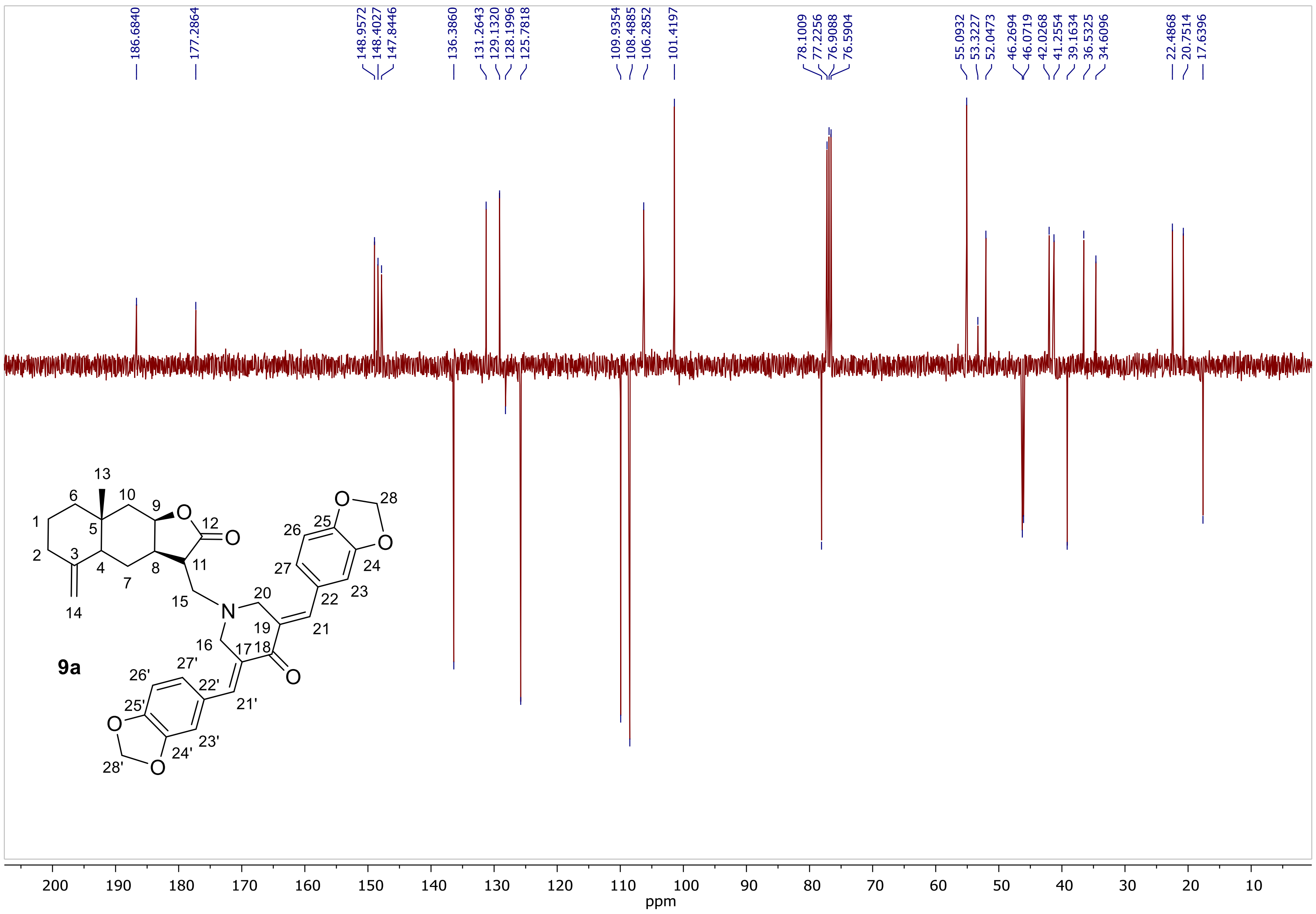

^{1}H and ^{13}C NMR spectraofcompound**9a**($CDCl_3$)

9aHRMS (ESI): m/z calcd. for $C_{36}H_{38}NO_7$ $[M + H^+]^+$: 596.2648, found 596.2633.

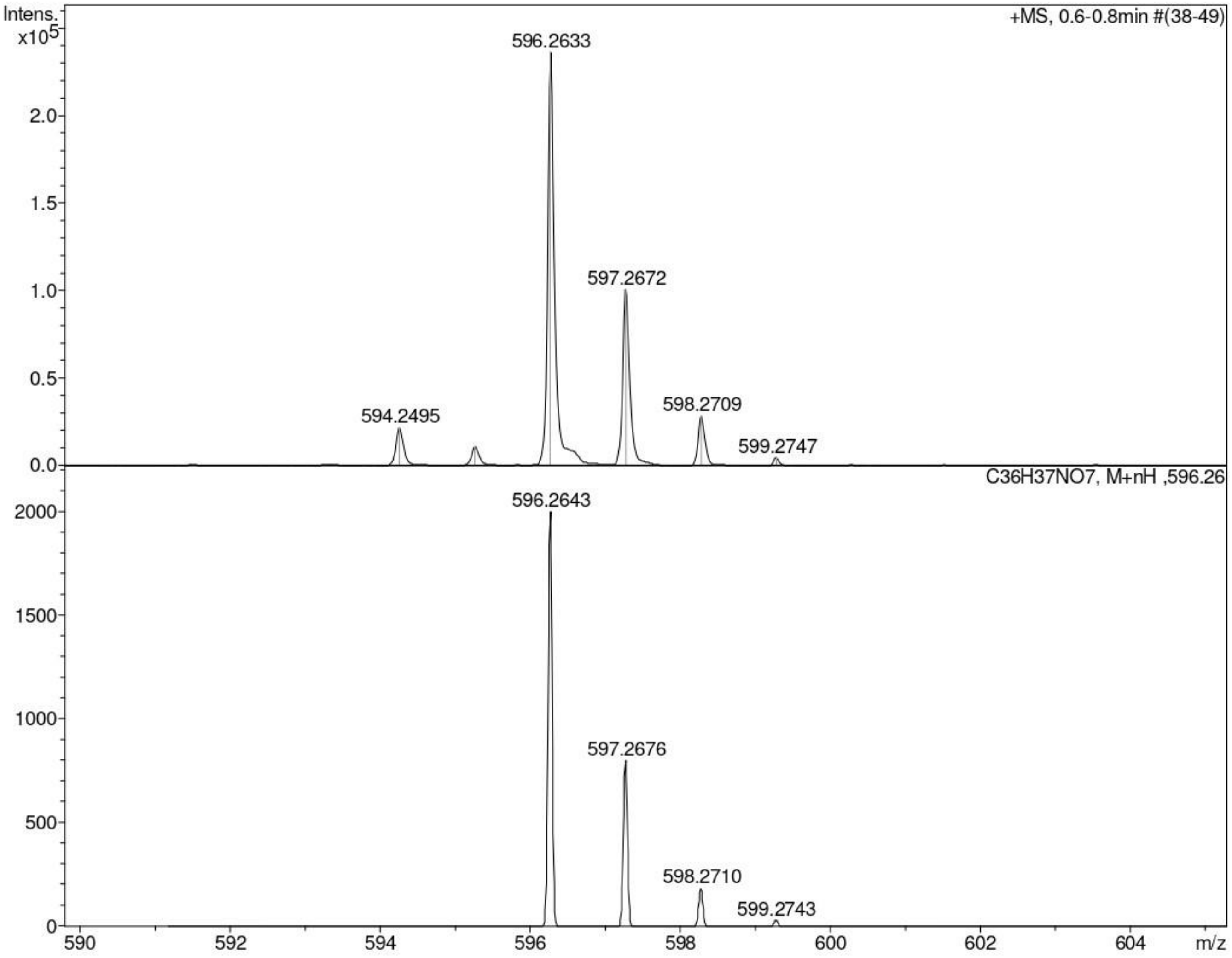

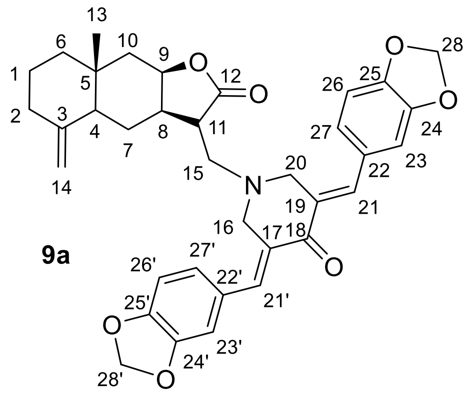

9a

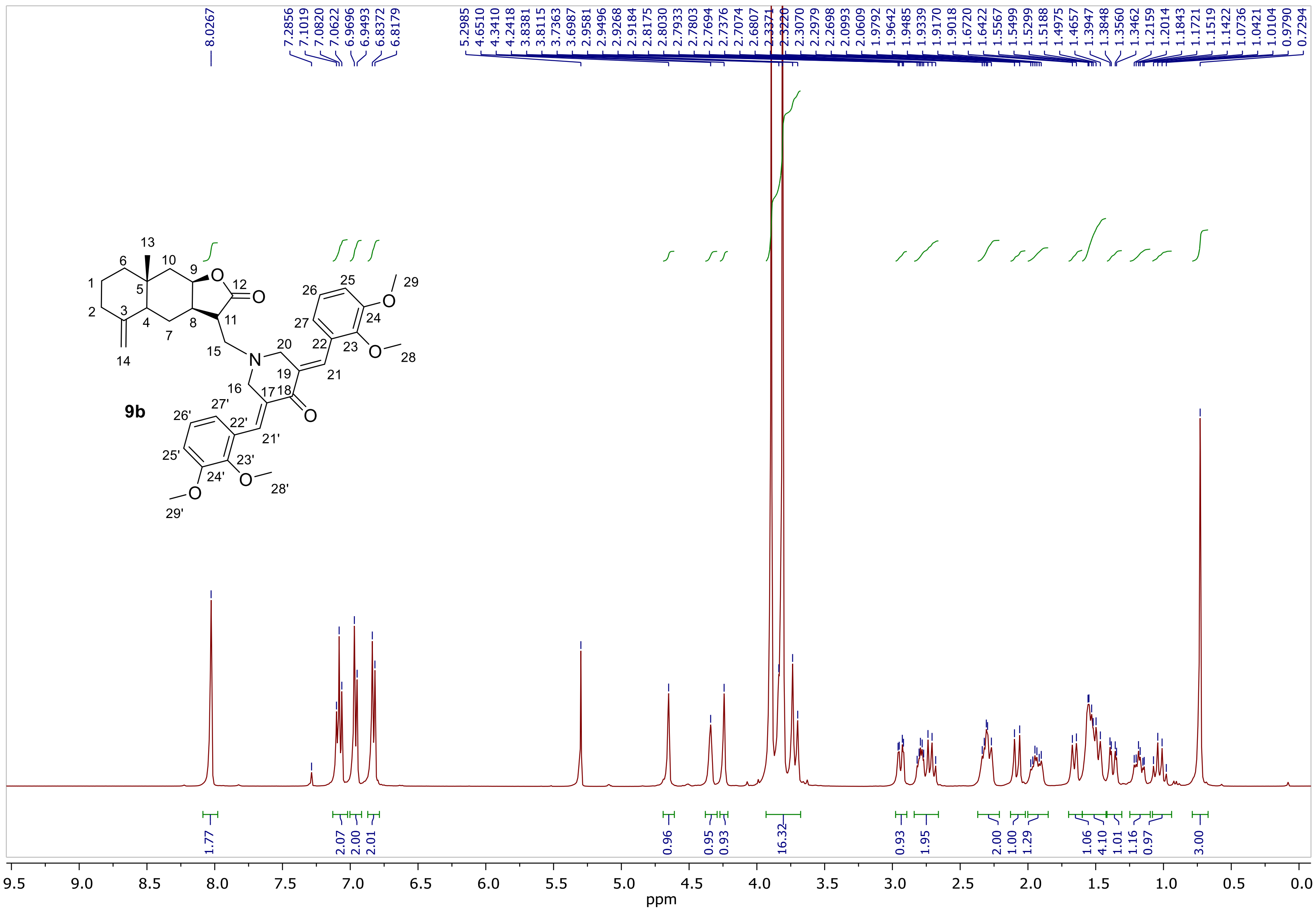
8.0267
7.2856 7.1019 7.0820 7.0622 6.9696 6.9493 6.8372 6.8179
5.2985 4.6510 4.3410 4.2418 3.8381 3.8115 3.7363 3.6987 2.9581 2.9496 2.9268 2.9184 2.8175 2.8030 2.7933 2.7803 2.7694 2.7376 2.7074 2.6807 2.3371 2.3220 2.3070 2.2979 2.2698 2.0993 2.0609 1.9792 1.9642 1.9485 1.9339 1.9170 1.9018 1.6720 1.6422 1.5567 1.5499 1.5299 1.5188 1.4975 1.4657 1.3947 1.3848 1.3560 1.3462 1.2159 1.2014 1.1843 1.1721 1.1519 1.1422 1.0736 1.0421 1.0104 0.9790 0.7294
9b
1.77 2.07 2.00 2.01 0.96 0.95 0.93 16.32 0.93 1.95 2.00 1.00 1.29 1.06 4.10 1.01 1.16 0.97 3.00
9.5 9.0 8.5 8.0 7.5 7.0 6.5 6.0 5.5 5.0 4.5 4.0 3.5 3.0 2.5 2.0 1.5 1.0 0.5 0.0
ppm

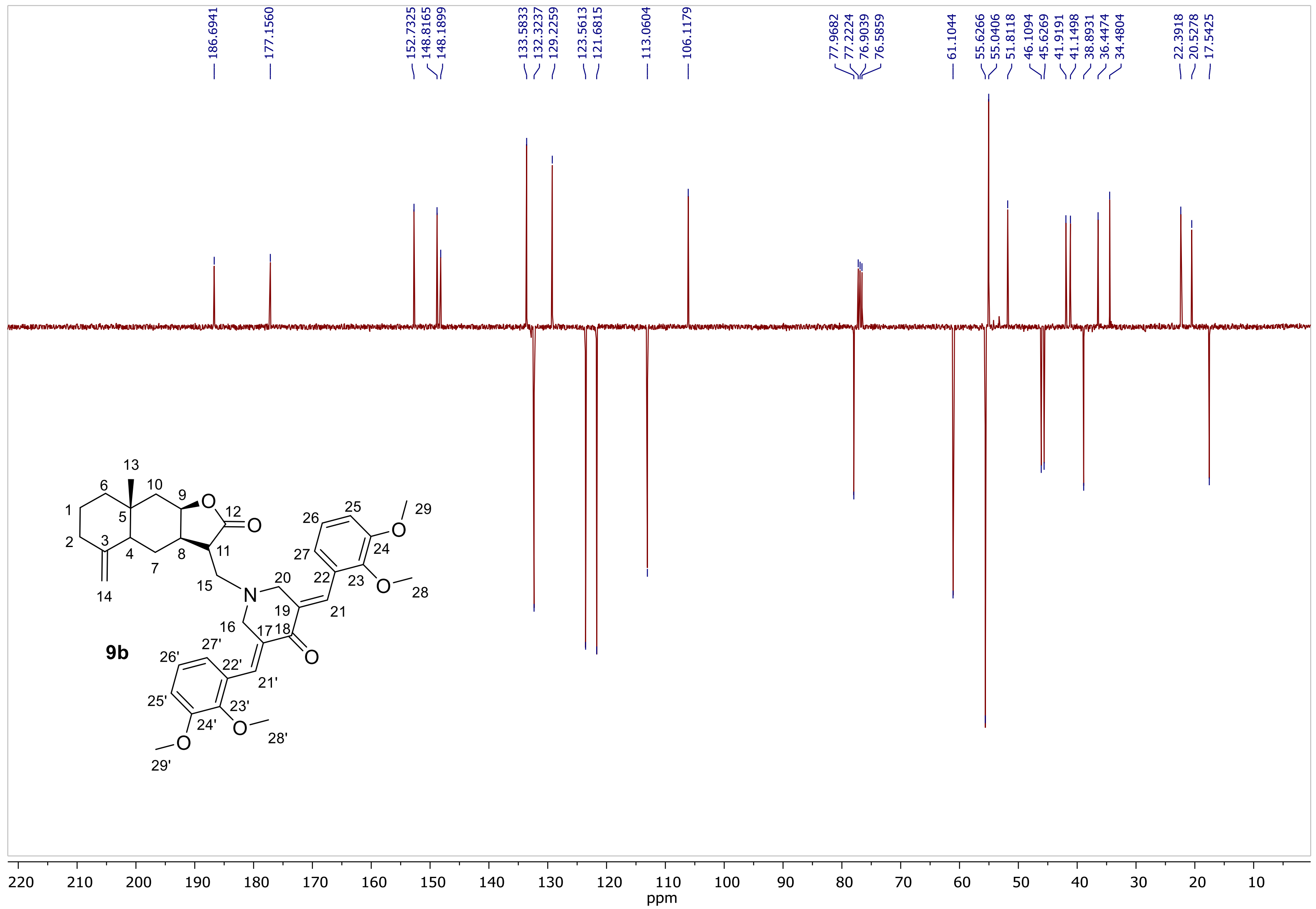

^{1}H and^{13}C NMR spectraofcompound**9b**(CDCl$_3$)

9b HRMS (ESI): m/z calcd. for $C_{38}H_{46}NO_7$ [M + H$^+$]$^+$: 628.3274, found 628.3278.

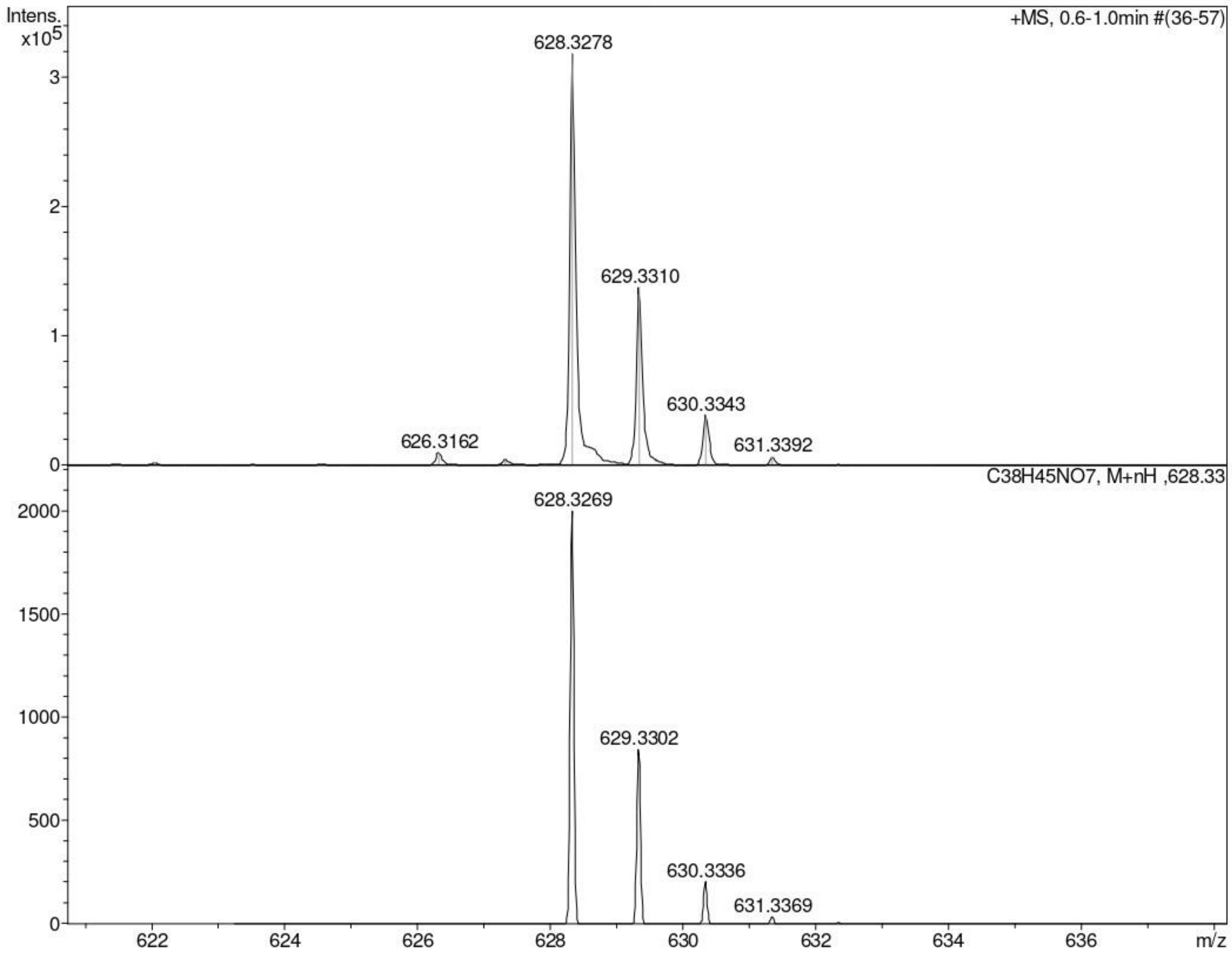

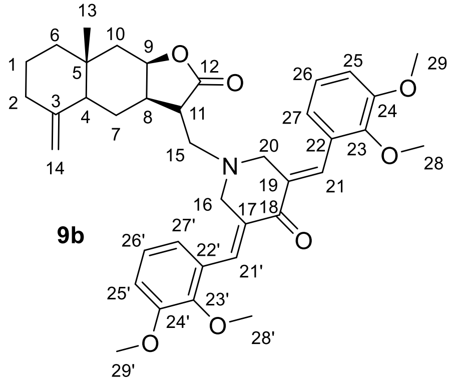

9b

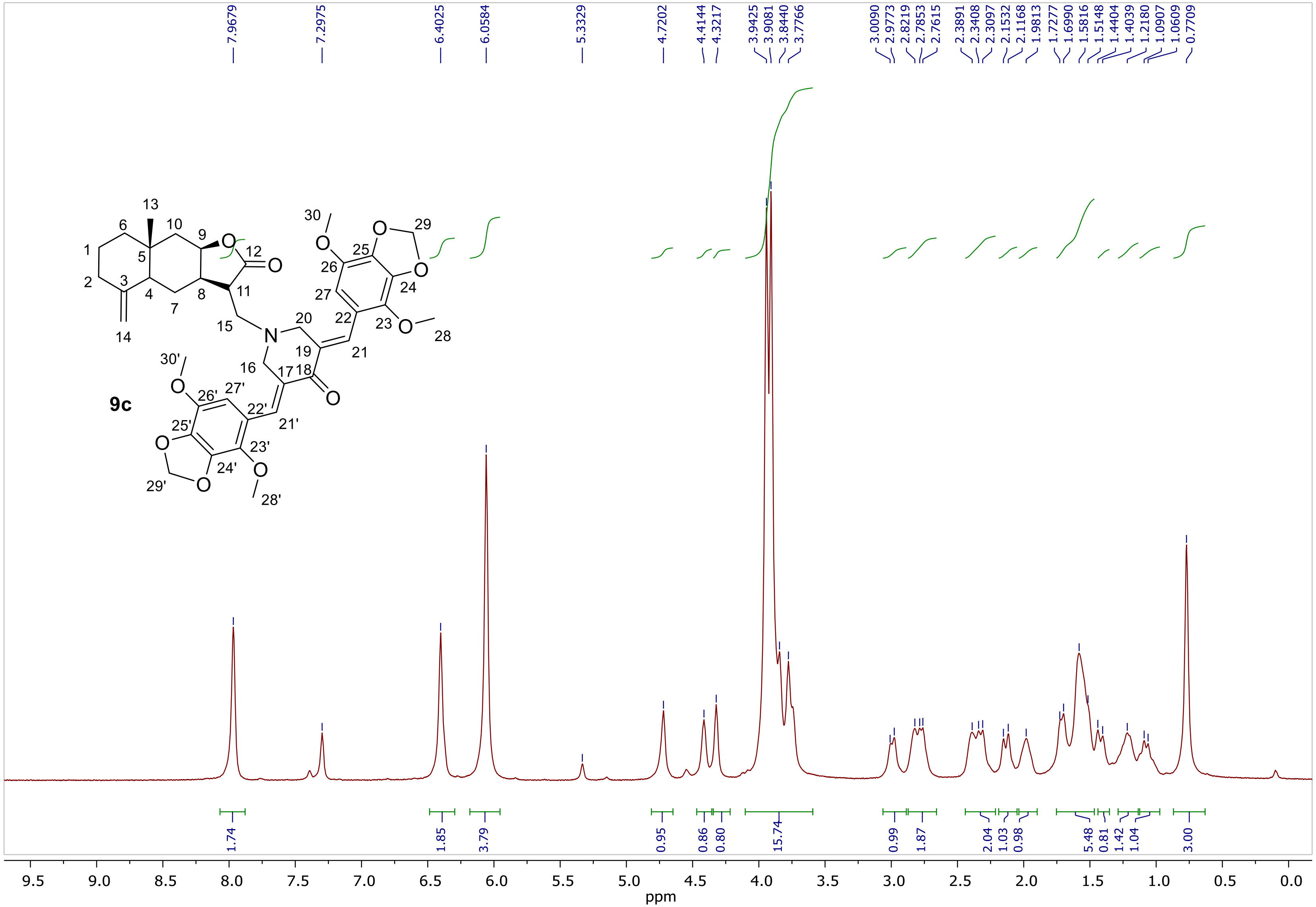
7.9679
7.2975
6.4025
6.0584
5.3329
4.7202
4.4144
4.3217
3.9425
3.9081
3.8440
3.7766
3.0090
2.9773
2.8219
2.7853
2.7615
2.3891
2.3408
2.3097
2.1532
2.1168
1.9813
1.7277
1.6990
1.5816
1.5148
1.4404
1.4039
1.2180
1.0907
1.0609
0.7709
9c
1.74
1.85
3.79
0.95
0.86
0.80
15.74
0.99
1.87
2.04
1.03
0.98
5.48
0.81
1.42
1.04
3.00
9.5 9.0 8.5 8.0 7.5 7.0 6.5 6.0 5.5 5.0 4.5 4.0 3.5 3.0 2.5 2.0 1.5 1.0 0.5 0.0
ppm

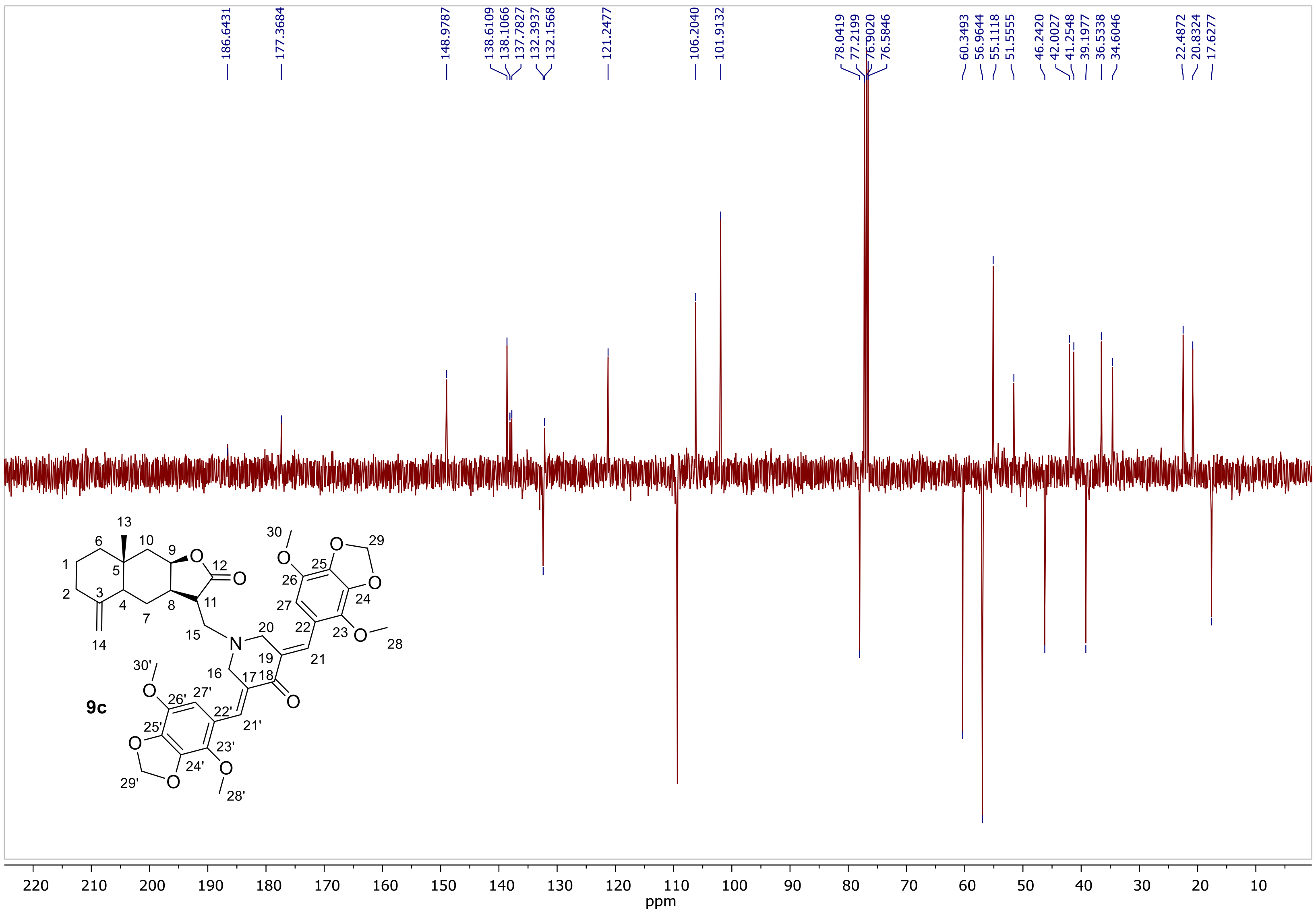

^{1}H and^{13}C NMR spectraofcompound**9c**($CDCl_3$)

9cHRMS (ESI): m/z calcd. for $C_{40}H_{46}NO_{11}$ [M + H$^+$]$^+$: 716.3071, found 716.3051.

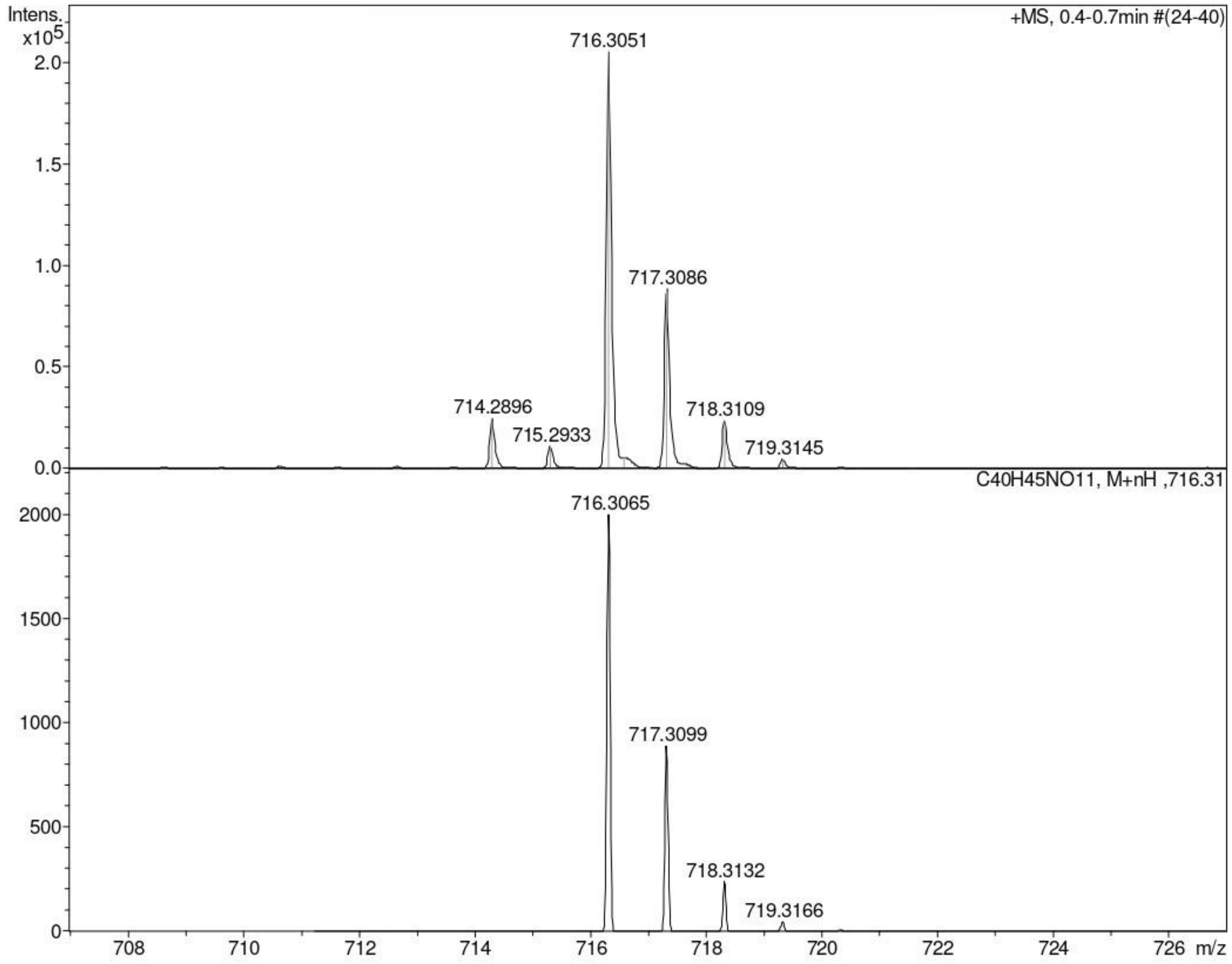

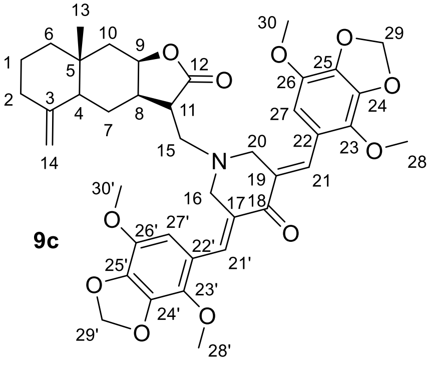

9c

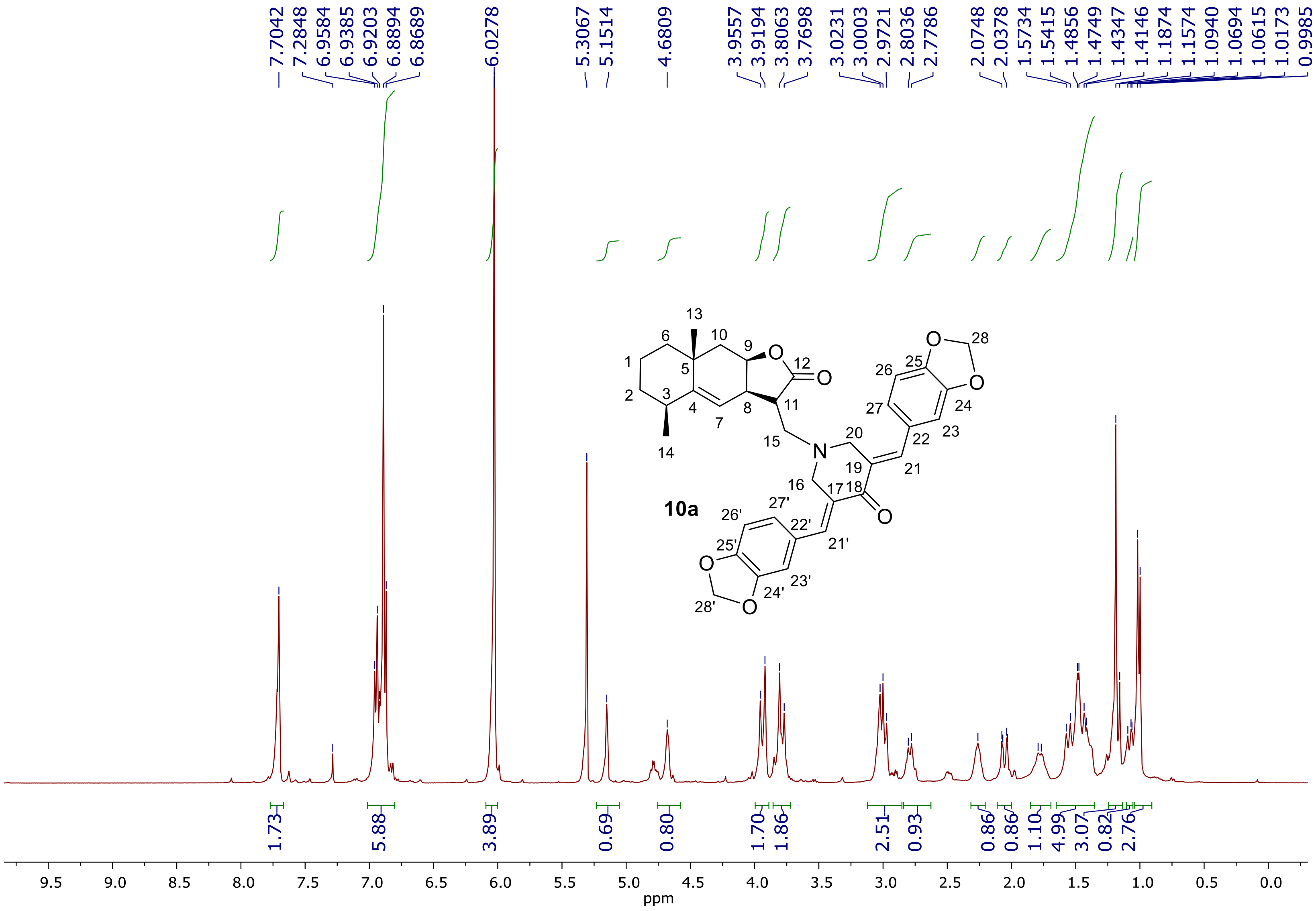
7.7042
7.2848
6.9584
6.9385
6.9203
6.8894
6.8689
6.0278
5.3067
5.1514
4.6809
3.9557
3.9194
3.8063
3.7698
3.0231
3.0003
2.9721
2.8036
2.7786
2.0748
2.0378
1.5734
1.5415
1.4856
1.4749
1.4347
1.4146
1.1874
1.1574
1.0940
1.0694
1.0615
1.0173
0.9985
10a
1.73
5.88
3.89
0.69
0.80
1.70
1.86
2.51
0.93
0.86
0.86
1.10
4.99
3.07
0.82
2.76
9.5
9.0
8.5
8.0
7.5
7.0
6.5
6.0
5.5
5.0
4.5
4.0
3.5
3.0
2.5
2.0
1.5
1.0
0.5
0.0
ppm

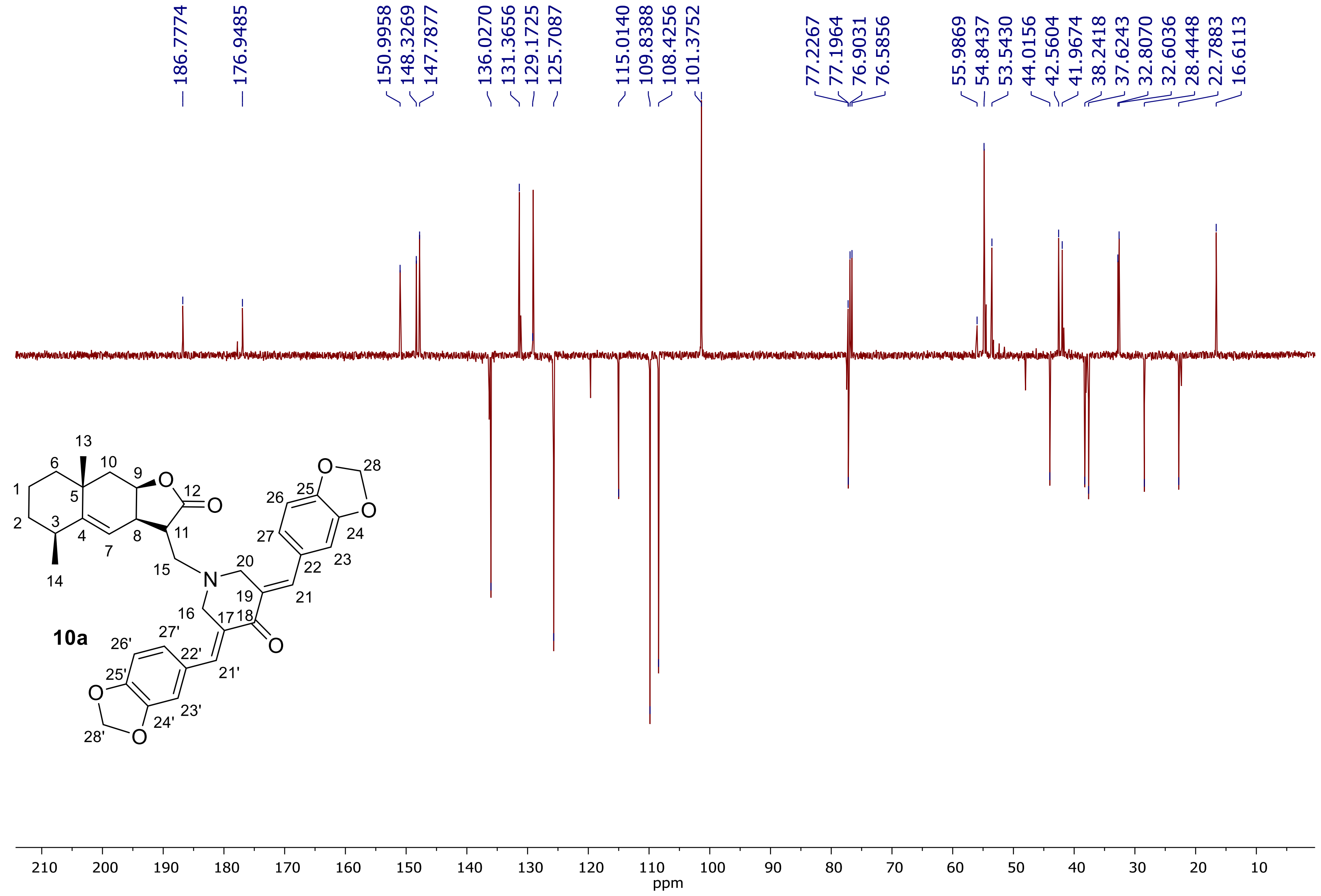

^{1}H and^{13}C NMR spectra of compound **10a** ($CDCl_3$)

10aHRMS (ESI): m/z calcd. for $C_{36}H_{38}NO_7$ [M + H$^+$]$^+$: 596.2648, found 596.2651.

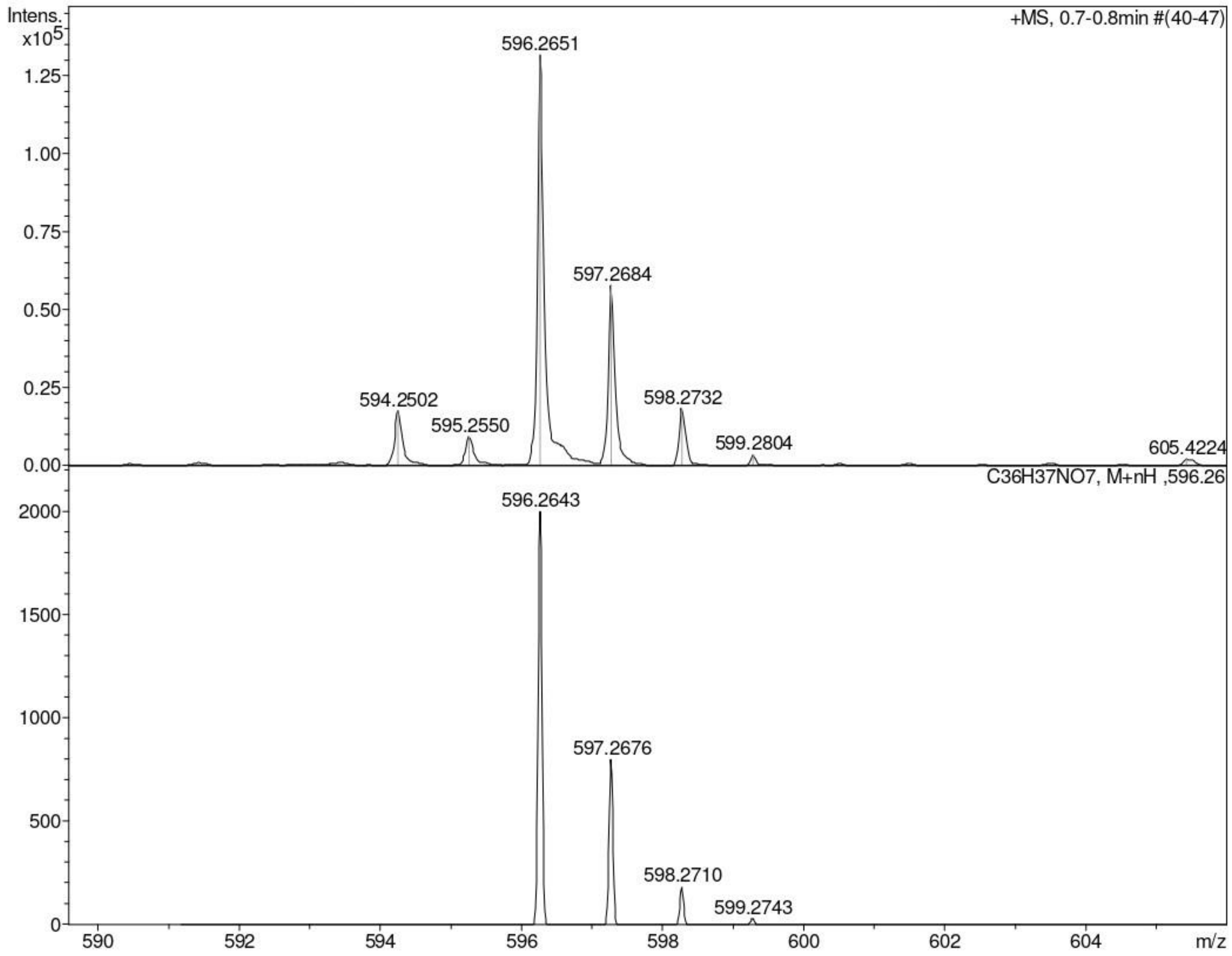

Intens.
x10^5
+MS, 0.7-0.8min #(40-47)
596.2651
597.2684
594.2502
595.2550
598.2732
599.2804
605.4224
C36H37NO7, M+nH ,596.26
596.2643
597.2676
598.2710
599.2743
m/z

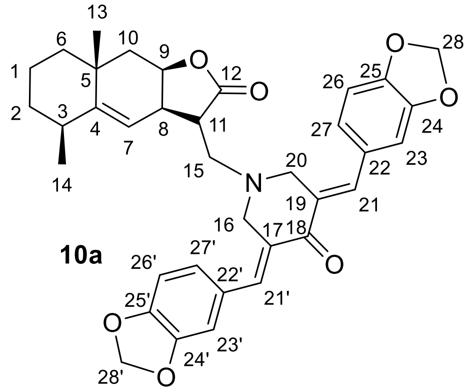

10a

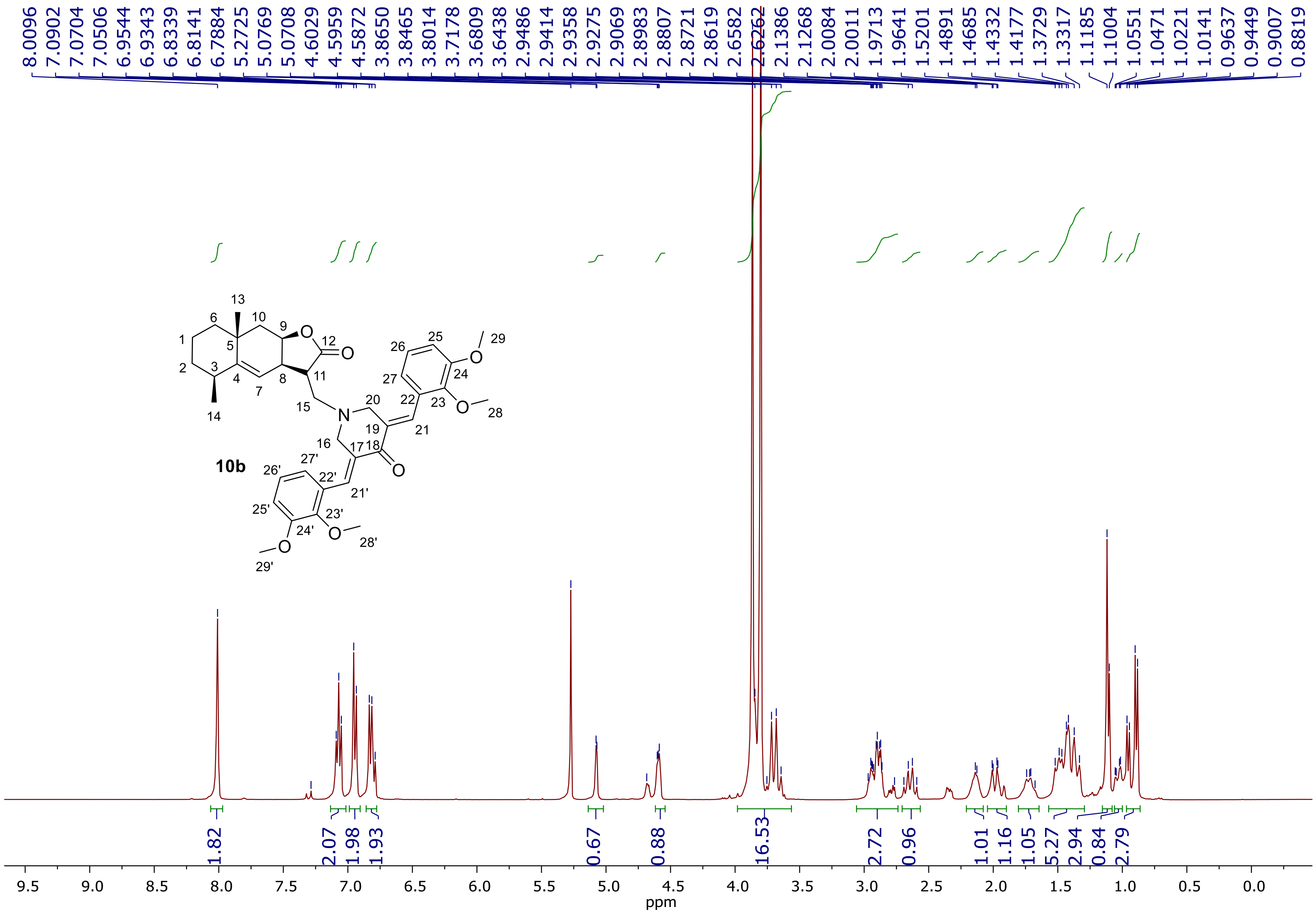
8.0096
7.0902
7.0704
7.0506
6.9544
6.9343
6.8339
6.8141
6.7884
5.2725
5.0769
5.0708
4.6029
4.5959
4.5872
3.8650
3.8465
3.8014
3.7178
3.6809
3.6438
2.9486
2.9414
2.9358
2.9275
2.9069
2.8983
2.8807
2.8721
2.8619
2.6582
2.6262
2.1386
2.1268
2.0084
2.0011
1.9713
1.9641
1.5201
1.4891
1.4685
1.4332
1.4177
1.3729
1.3317
1.1185
1.1004
1.0551
1.0471
1.0221
1.0141
0.9637
0.9449
0.9007
0.8819
10b
1.82
2.07
1.98
1.93
0.67
0.88
16.53
2.72
0.96
1.01
1.16
1.05
5.27
2.94
0.84
2.79
9.5 9.0 8.5 8.0 7.5 7.0 6.5 6.0 5.5 5.0 4.5 4.0 3.5 3.0 2.5 2.0 1.5 1.0 0.5 0.0
ppm

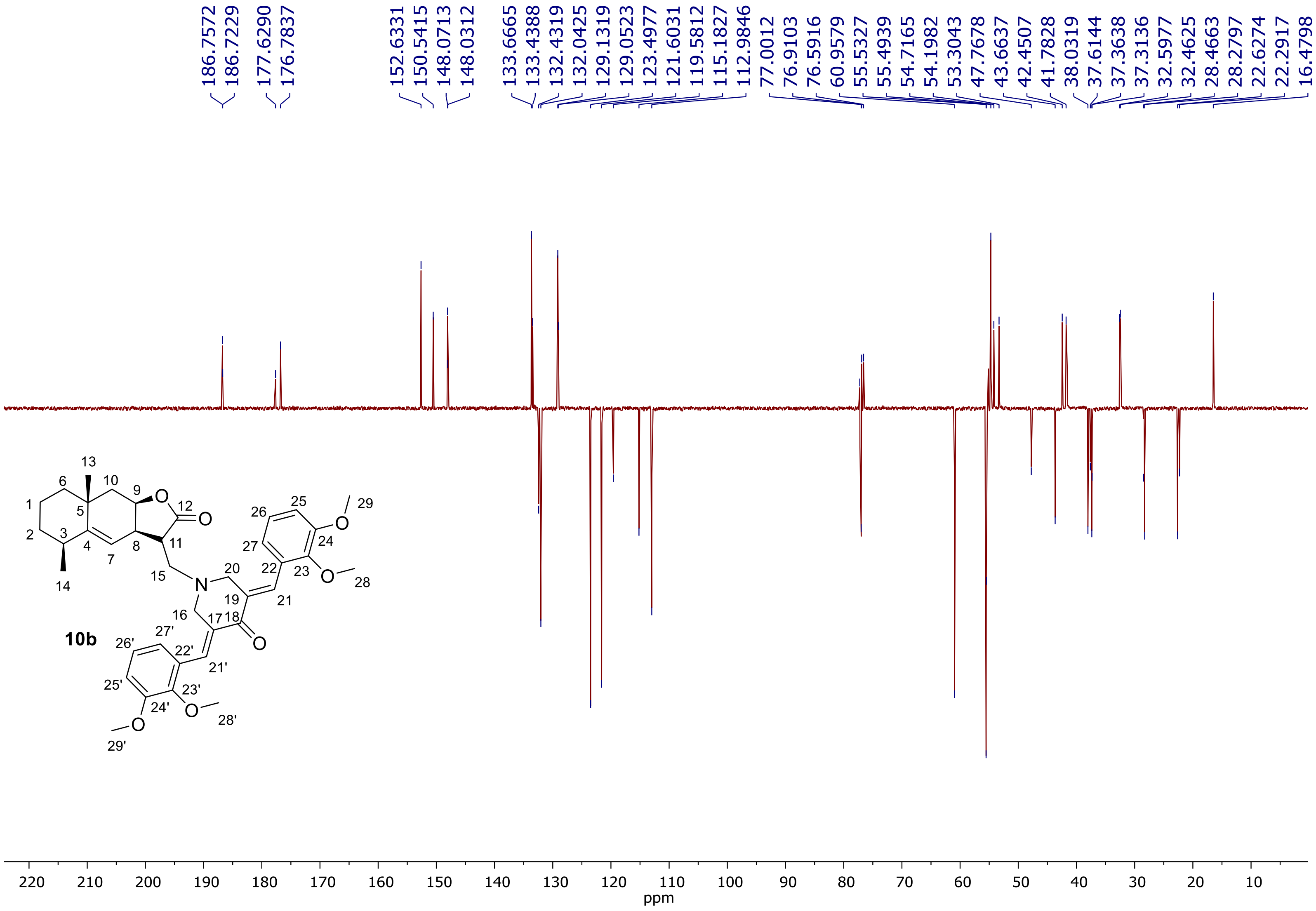

^{1}H and^{13}C NMR spectra of compound **10b** ($CDCl_3$)

10bHRMS (ESI): m/z calcd. for $C_{38}H_{46}NO_7$ [M + H$^+$]$^+$: 628.3274, found 628.3264.

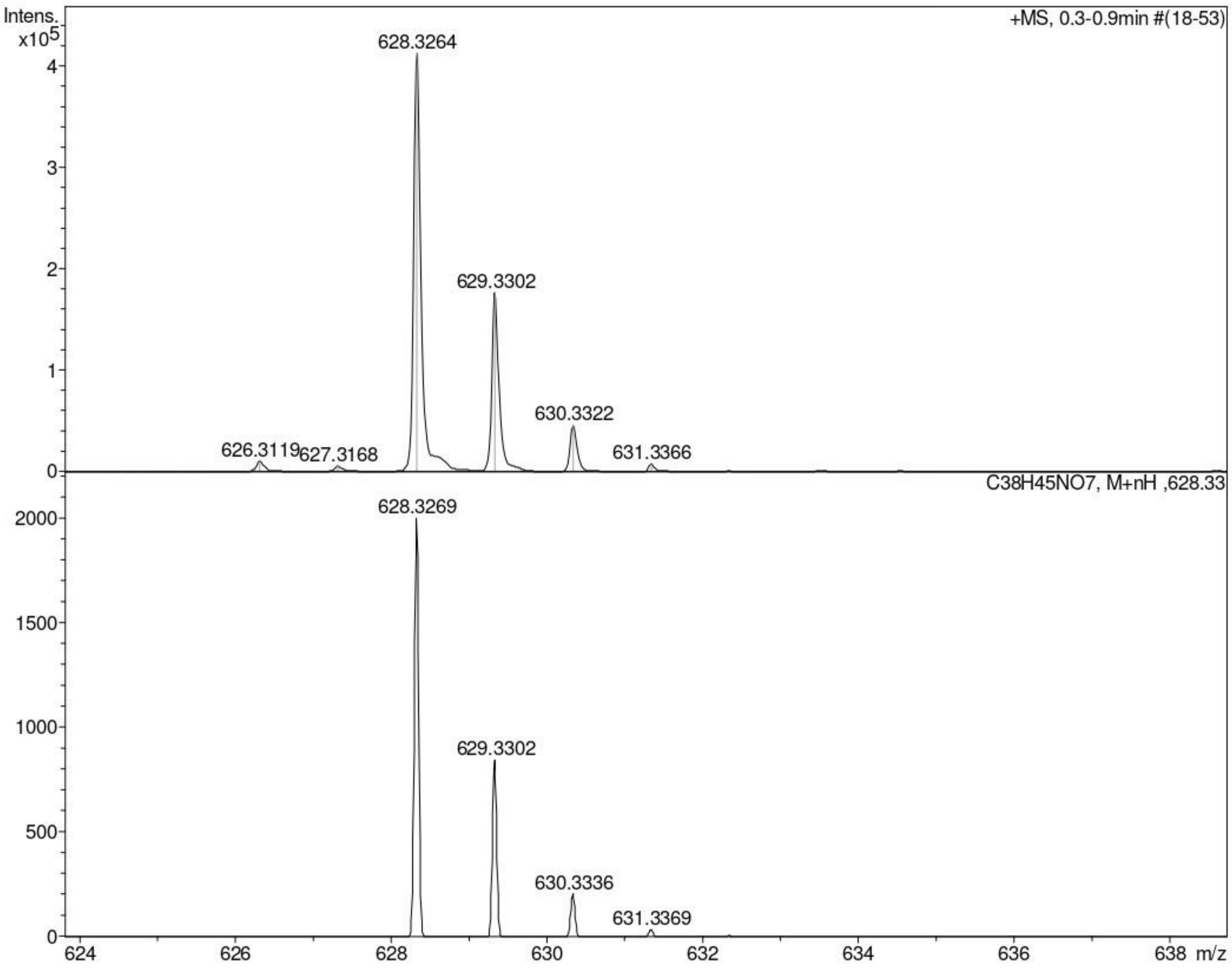
Intens.
x10^5
+MS, 0.3-0.9min #(18-53)
628.3264
629.3302
630.3322
631.3366
626.3119
627.3168
C38H45NO7, M+nH ,628.33
628.3269
629.3302
630.3336
631.3369
m/z

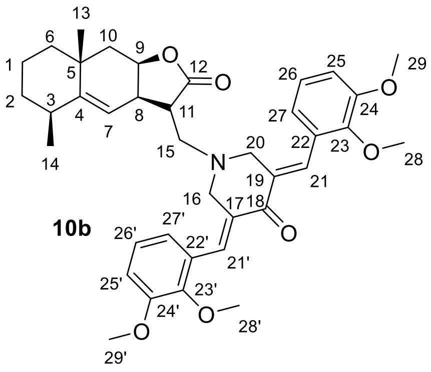

10b

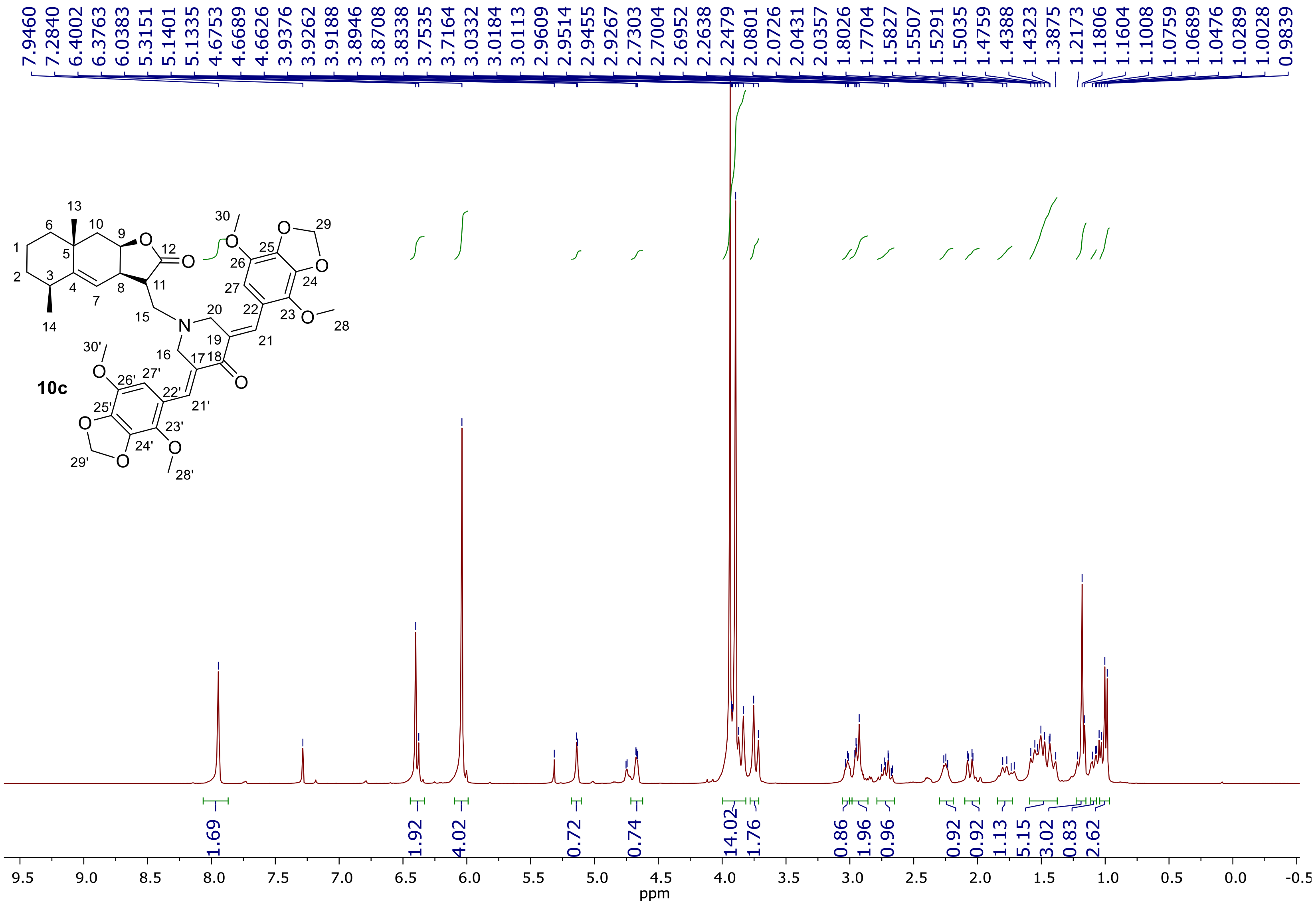
7.9460 7.2840 6.4002 6.3763 6.0383 5.3151 5.1401 5.1335 4.6753 4.6689 4.6626 3.9376 3.9262 3.9188 3.8946 3.8708 3.8338 3.7535 3.7164 3.0332 3.0184 3.0113 2.9609 2.9514 2.9455 2.9267 2.7303 2.7004 2.6952 2.2638 2.2479 2.0801 2.0726 2.0431 2.0357 1.8026 1.7704 1.5827 1.5507 1.5291 1.5035 1.4759 1.4388 1.4323 1.3875 1.2173 1.1806 1.1604 1.1008 1.0759 1.0689 1.0476 1.0289 1.0028 0.9839
10c
1.69 1.92 4.02 0.72 0.74 14.02 1.76 0.86 1.96 0.96 0.92 0.92 1.13 5.15 3.02 0.83 2.62
9.5 9.0 8.5 8.0 7.5 7.0 6.5 6.0 5.5 5.0 4.5 4.0 3.5 3.0 2.5 2.0 1.5 1.0 0.5 0.0 -0.5
ppm

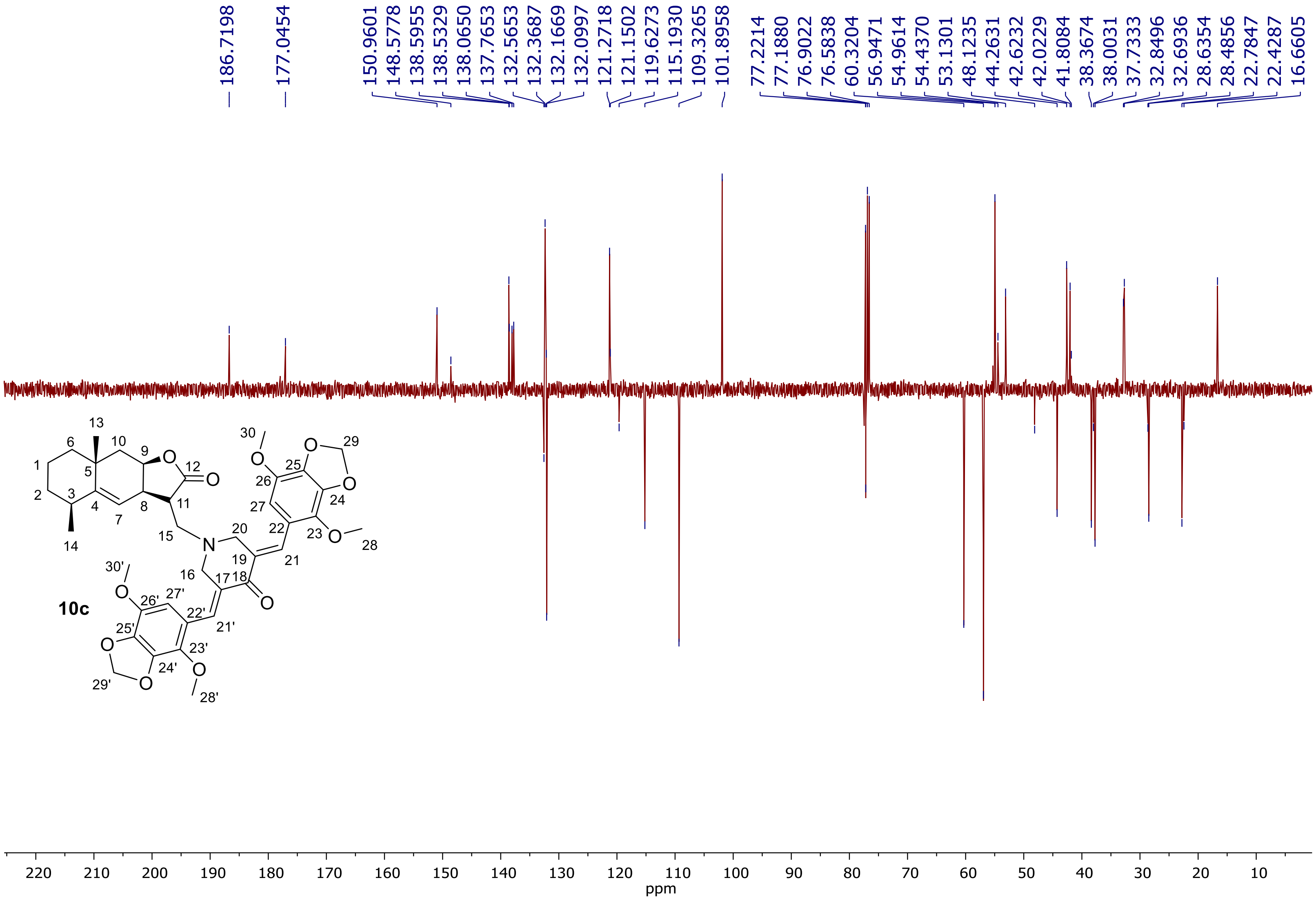

^{1}H and^{13}C NMR spectra of compound **10c** ($CDCl_3$)

10cHRMS (ESI): m/z calcd. for $C_{40}H_{46}NO_{11}$ [M + H$^+$]$^+$: 716.3071, found 716.3059.

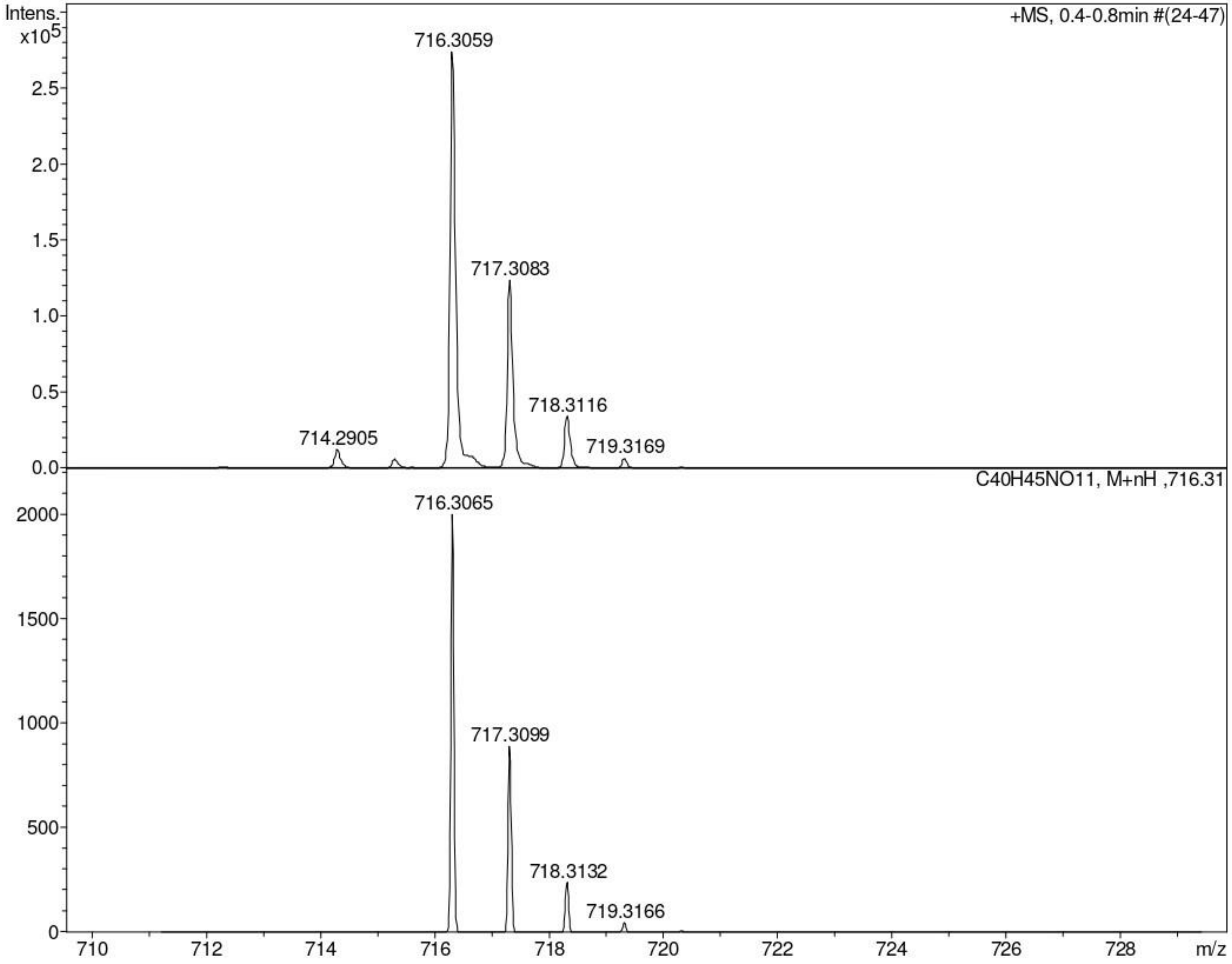
Intens.
x10^5
+MS, 0.4-0.8min #(24-47)
716.3059
717.3083
718.3116
714.2905
719.3169
C40H45NO11, M+nH ,716.31
716.3065
717.3099
718.3132
719.3166
m/z

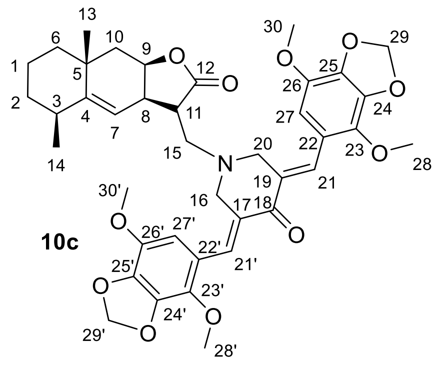

10c

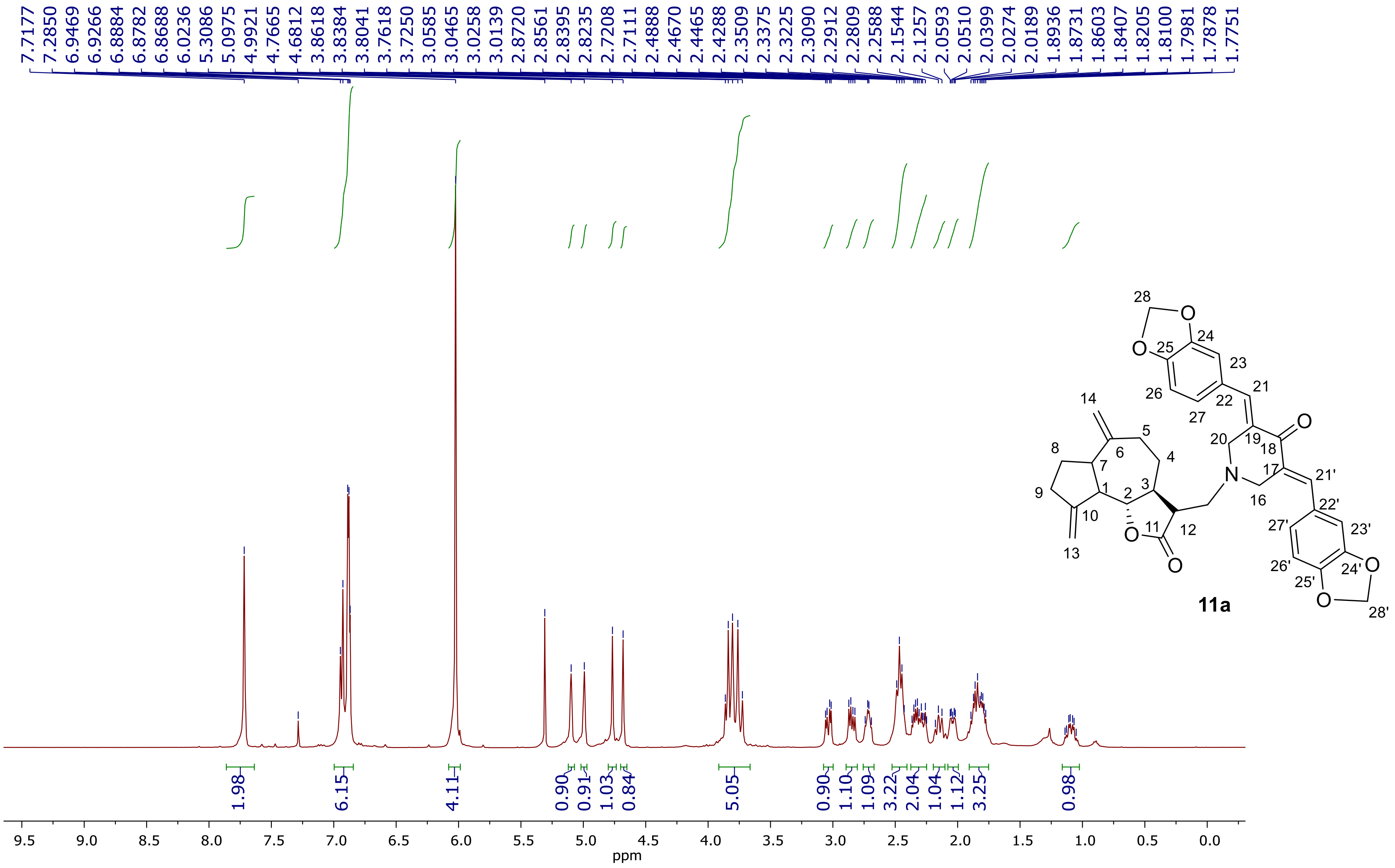
7.7177
7.2850
6.9469
6.9266
6.8884
6.8782
6.8688
6.0236
5.3086
5.0975
4.9921
4.7665
4.6812
3.8618
3.8384
3.8041
3.7618
3.7250
3.0585
3.0465
3.0258
3.0139
2.8720
2.8561
2.8395
2.8235
2.7208
2.7111
2.4888
2.4670
2.4465
2.4288
2.3509
2.3375
2.3225
2.3090
2.2912
2.2809
2.2588
2.1544
2.1257
2.0593
2.0510
2.0399
2.0274
2.0189
1.8936
1.8731
1.8603
1.8407
1.8205
1.8100
1.7981
1.7878
1.7751
1.98
6.15
4.11
0.90
0.91
1.03
0.84
5.05
0.90
1.10
1.09
3.22
2.04
1.04
1.12
3.25
0.98
9.5 9.0 8.5 8.0 7.5 7.0 6.5 6.0 5.5 5.0 4.5 4.0 3.5 3.0 2.5 2.0 1.5 1.0 0.5 0.0
ppm
11a

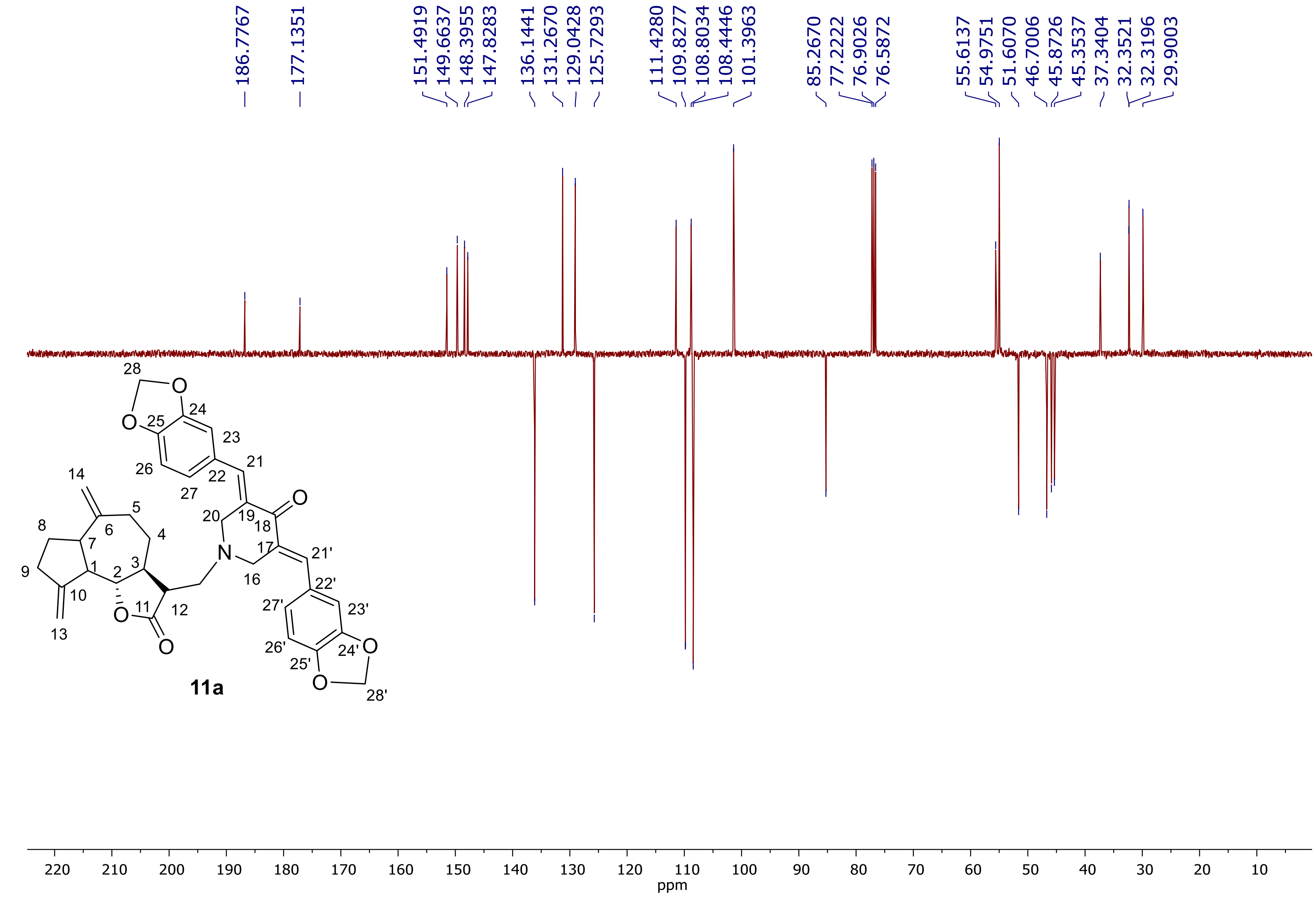

11a

^{1}H and^{13}C NMR spectra of compound **11a** ($CDCl_3$)

11aHRMS (ESI): m/z calcd. for $C_{36}H_{36}NO_7$ $[M + H^+]^+$: 594.2492, found 594.2483.

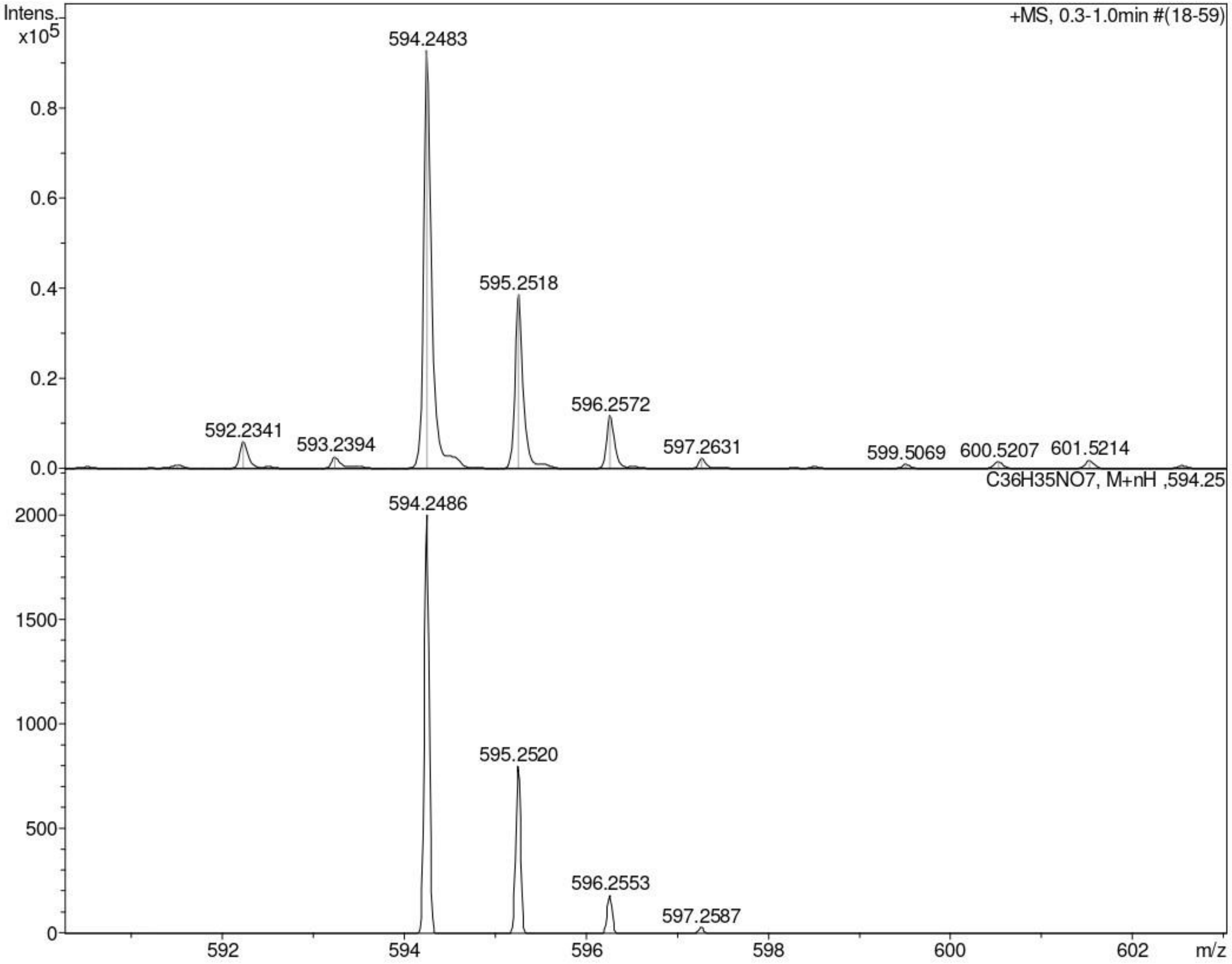

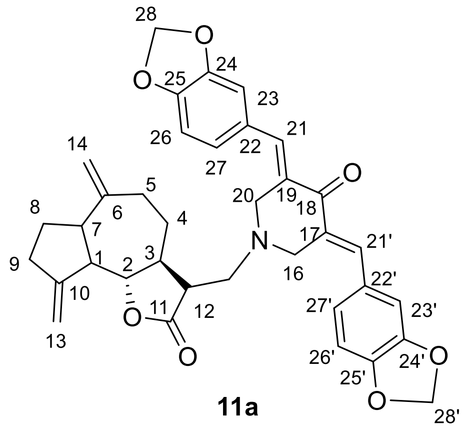

11a

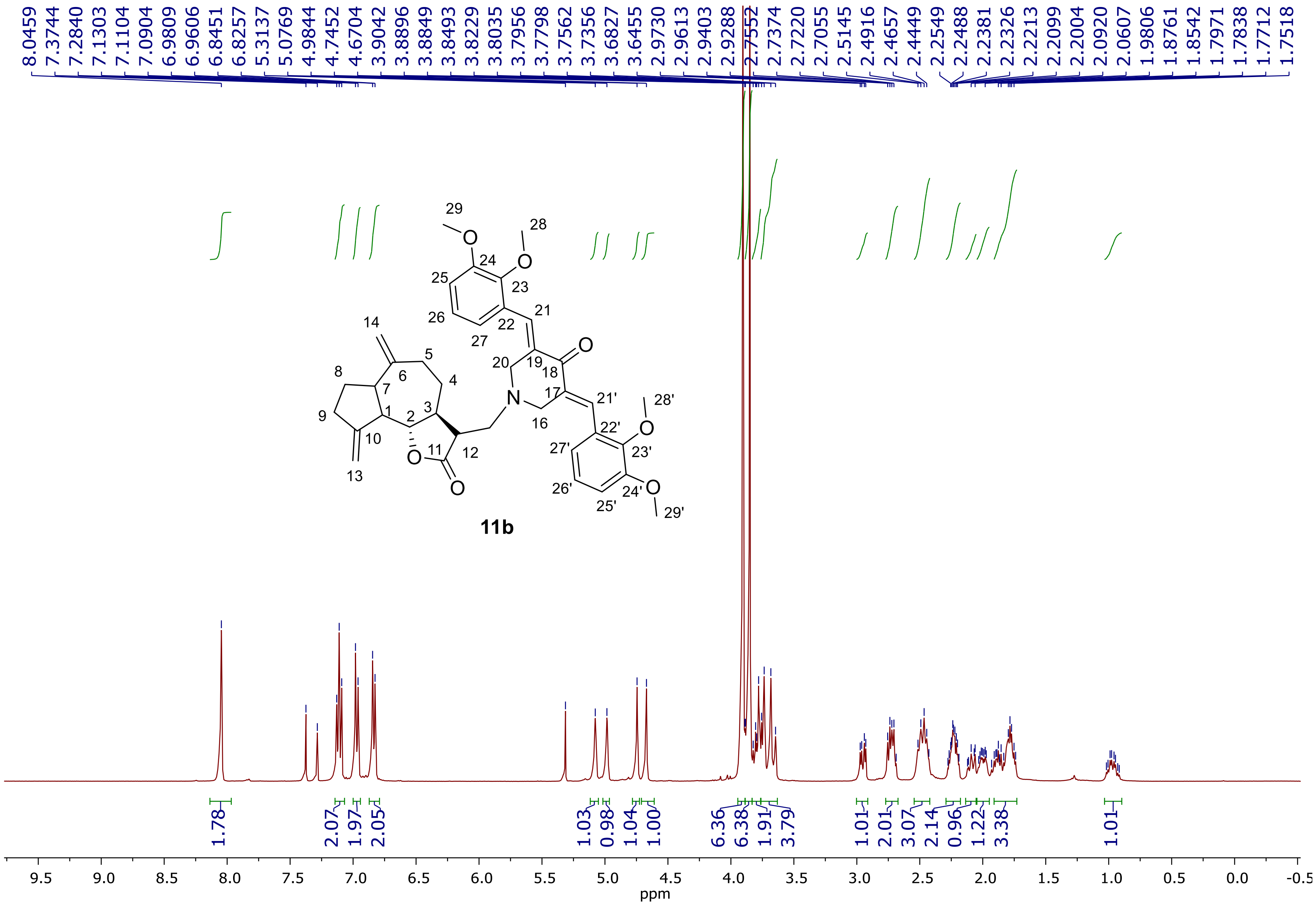
8.0459
7.3744
7.2840
7.1303
7.1104
7.0904
6.9809
6.9606
6.8451
6.8257
5.3137
5.0769
4.9844
4.7452
4.6704
3.9042
3.8896
3.8849
3.8493
3.8229
3.8035
3.7956
3.7798
3.7562
3.7356
3.6827
3.6455
2.9730
2.9613
2.9403
2.9288
2.7552
2.7374
2.7220
2.7055
2.5145
2.4916
2.4657
2.4449
2.2549
2.2488
2.2381
2.2326
2.2213
2.2099
2.2004
2.0920
2.0607
1.9806
1.8761
1.8542
1.7971
1.7838
1.7712
1.7518
1.78
2.07
1.97
2.05
1.03
0.98
1.04
1.00
6.36
6.38
1.91
3.79
1.01
2.01
3.07
2.14
0.96
1.22
3.38
1.01
9.5 9.0 8.5 8.0 7.5 7.0 6.5 6.0 5.5 5.0 4.5 4.0 3.5 3.0 2.5 2.0 1.5 1.0 0.5 0.0 -0.5
ppm
11b

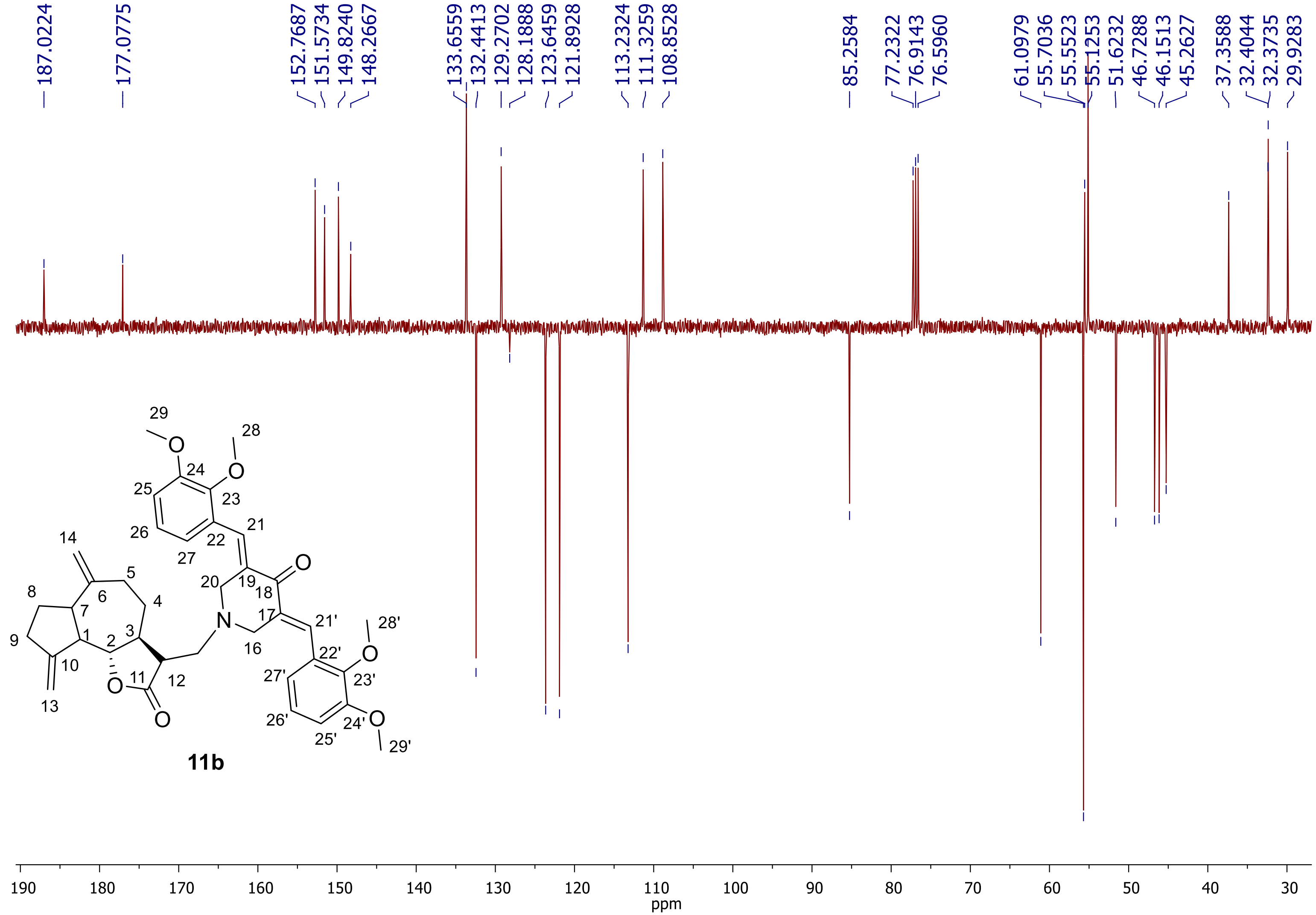

^{1}H and^{13}C NMR spectra of compound **11b** ($CDCl_3$)

11bHRMS (ESI): m/z calcd. for $C_{38}H_{44}NO_7$ [M + H^{+}]$^{+}$: 626.3118, found 626.3115.

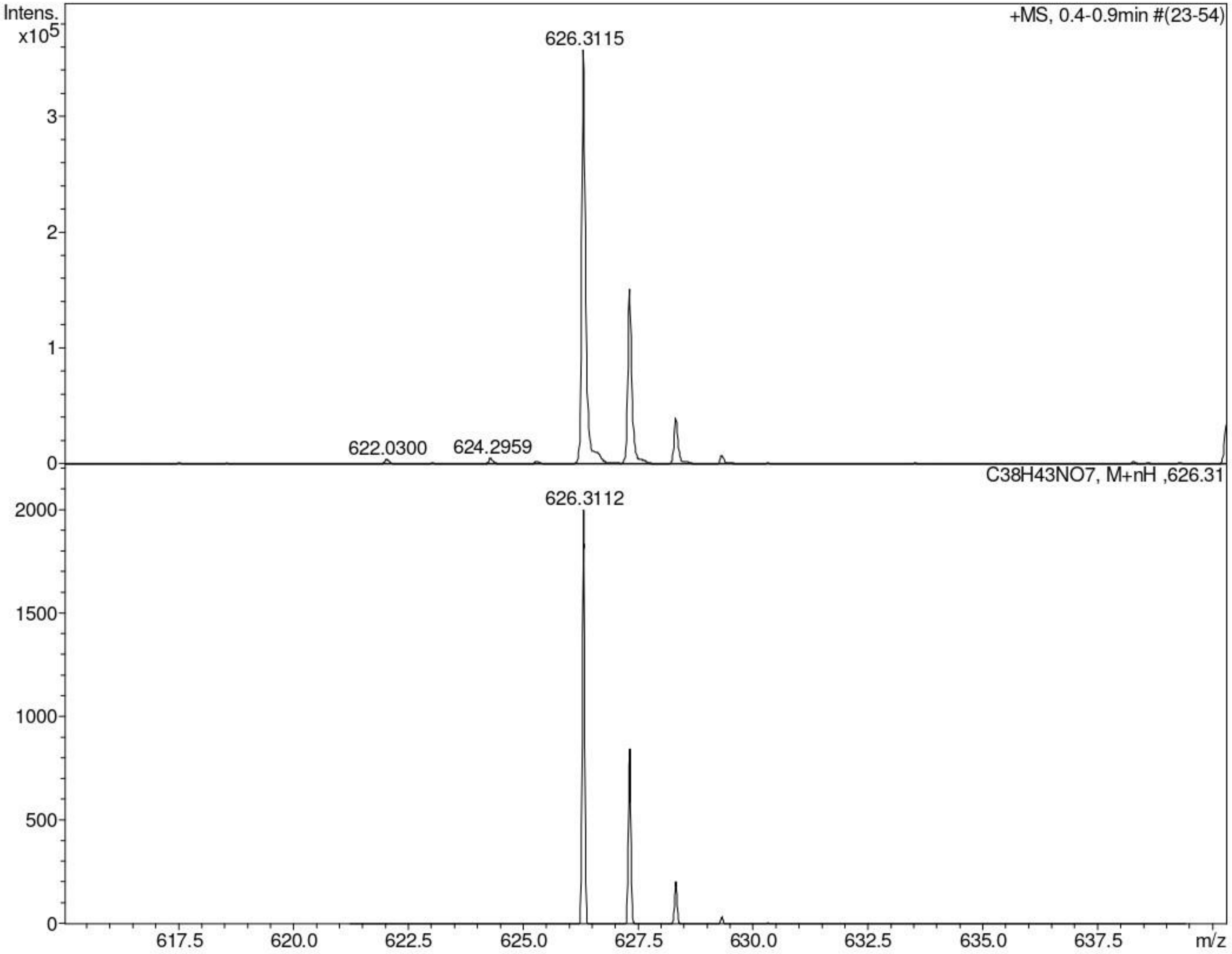

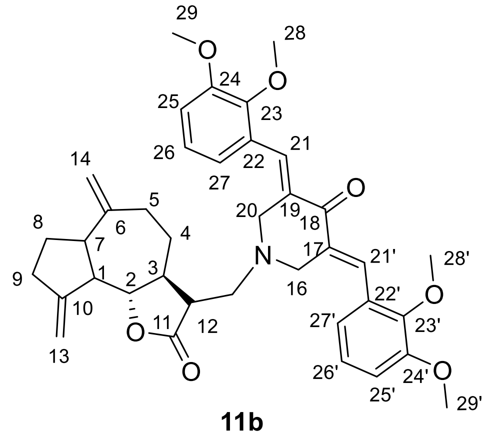

11b

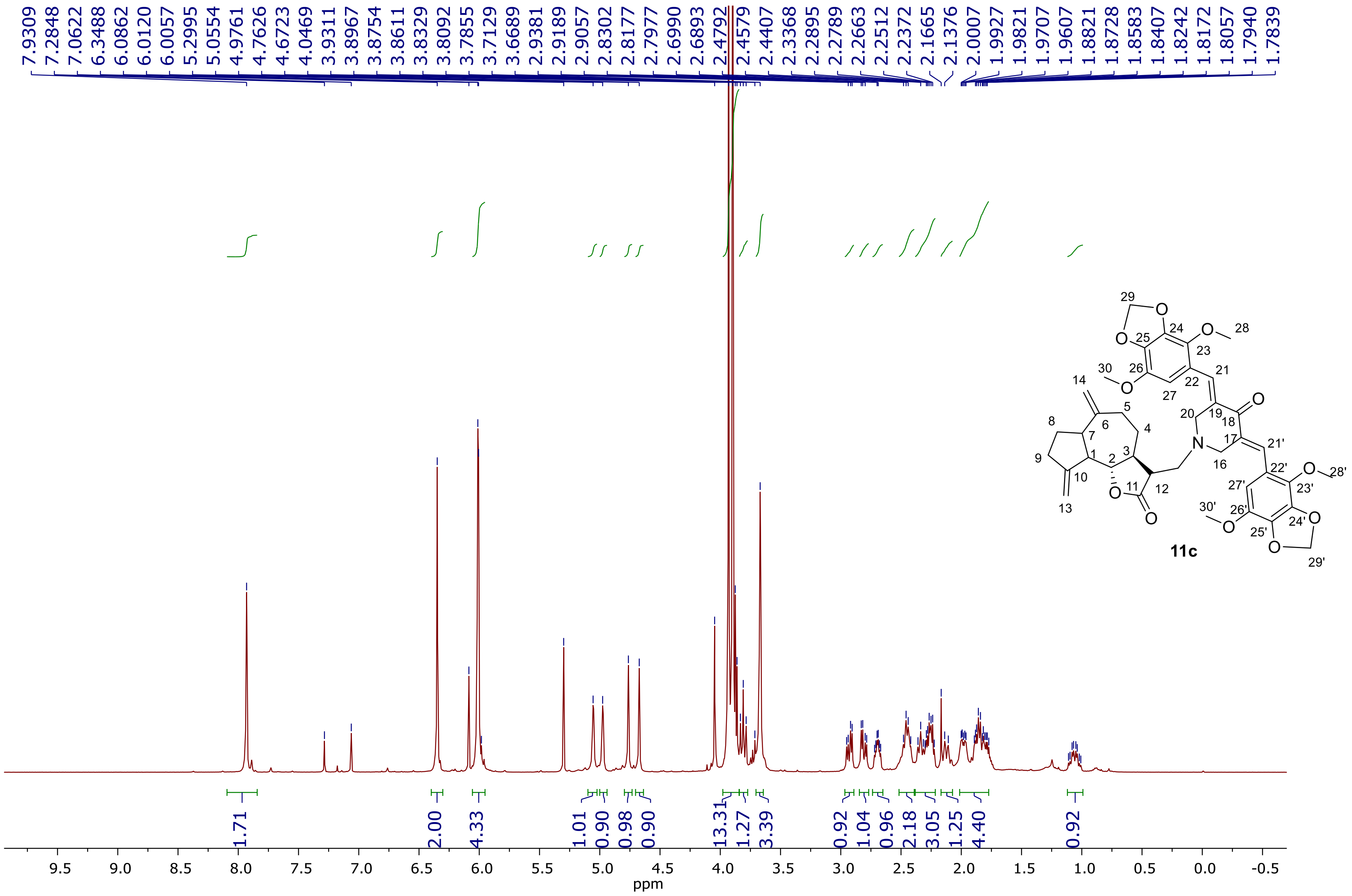

11c

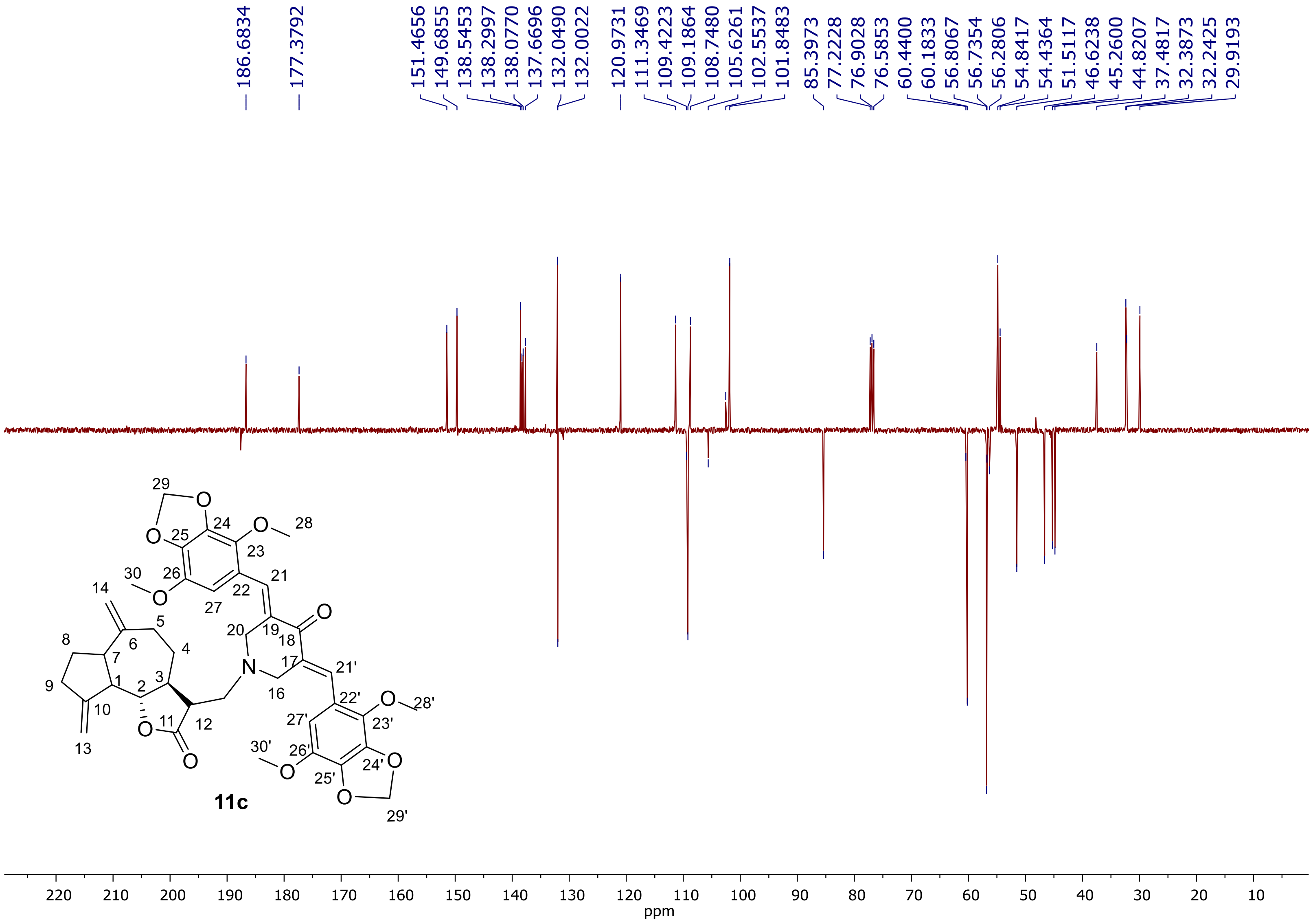

^{1}H and^{13}C NMR spectra of compound **11c** ($CDCl_3$)

11cHRMS (ESI): m/z calcd. for $C_{40}H_{44}NO_{11}$ [M + H$^+$]$^+$: 714.2914, found 714.2915.

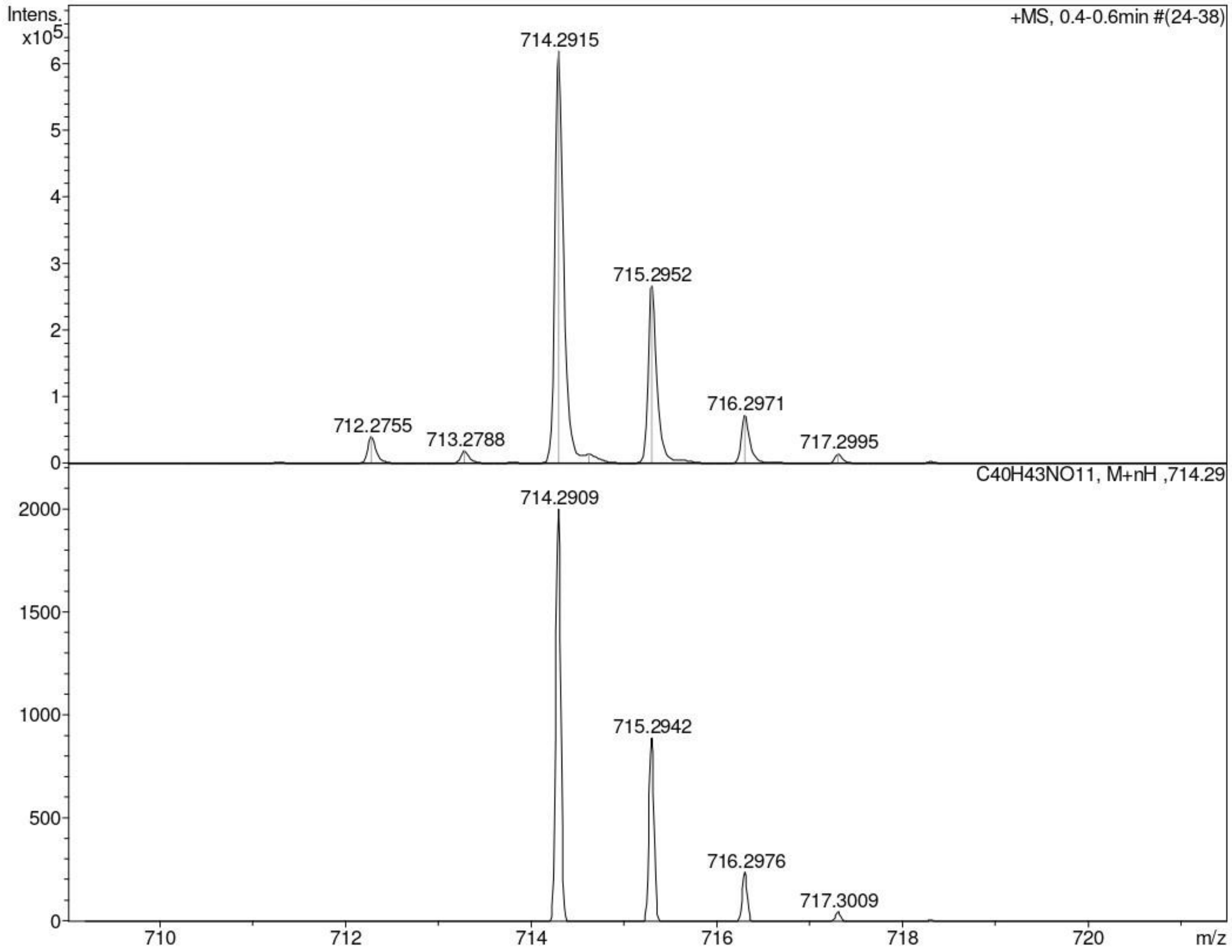

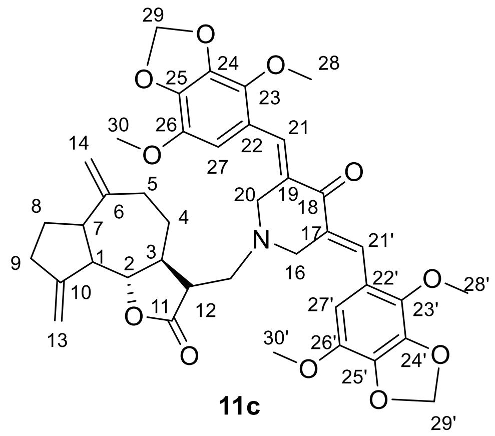

11c
